# Analysis of Boron-Based and Rare-Earth-Based Additive Strategies in Advanced Oxide Materials in Terms of Structural–Morphological Performance and Critical Raw Material Policies

**DOI:** 10.3390/nano16100639

**Published:** 2026-05-21

**Authors:** Berkay Gür, Haluk Yaman, Cevher Kürşat Macit

**Affiliations:** 1 Faculty of Economics and Administrative Sciences, Department of Politics and Social Sciences, Firat University, Elazig 23100, Türkiye; berkaygur01@gmail.com; 2 School of Aviation, Aircraft Airframe-Engine Maintenance, Firat University, Elazig 23119, Türkiye

**Keywords:** ZnO, boron doping, B_4_C, h-BN, CeO_2_, La_2_O_3_, Y_2_O_3_, advanced oxide materials, characterization synthesis, critical raw materials, supply security, public R&D policy

## Abstract

In advanced oxide materials, additive selection is increasingly constrained by the simultaneous requirements of functional response, phase stability, morphology control, processing tolerance, scalability, and critical raw material security. This study develops a ZnO-centered framework to compare boron-based strategies (direct B doping, B_4_C/ZnO composite formation, and h-BN/ZnO interface engineering) with rare-earth strategies (Ce/CeO_2_, La/La_2_O_3_, and Y/Y_2_O_3_). Structural, morphological, chemical-state, and vibrational evidence from XRD, FE-SEM/EDX, XPS, Raman, and FT-IR studies is interpreted through an evidence hierarchy that separates lattice incorporation, surface/grain-boundary segregation, and deliberate secondary-phase or heterointerface formation. The synthesis shows that boron-containing routes usually provide broader phase retention, lower agglomeration tendency, more gradual defect modulation, and greater processing robustness, whereas rare-earth routes offer stronger oxygen-vacancy regulation, redox activity, luminescence tuning, and heterojunction-assisted function but require tighter process control and more rigorous verification of incorporation mode. Reanalysis of seven primary experimental pathways indicates that B_4_C/ZnO and h-BN/ZnO are mechanistically non-equivalent: B_4_C supports rigid composite-interface growth, while h-BN promotes sheet-mediated interface multiplication and Maxwell–Wagner–Sillars polarization. Türkiye is treated as an illustrative boron-rich producer case within a transferable producer/importer decision model. Dopant selection is therefore framed as a multi-criteria decision involving performance thresholds, reproducibility, technology-readiness potential, and supply-security exposure, not peak output alone.

## 1. Introduction

Advanced oxide materials constitute one of the central research domains of contemporary materials science, owing to their broad application spectrum, extending from semiconducting systems and catalysis to gas sensing and optoelectronics. In particular, oxides such as ZnO, SnO_2_, Fe_2_O_3_, and NiO are highly amenable to doping strategies because of their defect chemistry, multiple oxidation state behavior, surface reactivity, and low-cost synthetic accessibility. Nevertheless, discussions of doping in the literature are often confined to an application and performance framework; the structural and morphological consequences of dopant incorporation, together with their broader implications for resource policy, supply security, and strategic dependence, are rarely addressed within the same study. Yet, material selection is no longer governed solely by laboratory scale performance, but also by access to critical raw materials, supply chain concentration, refining capacity, and public support mechanisms [1,2,3,4,5].

This transition has become particularly evident in the case of rare-earth elements. Elements such as cerium, lanthanum, and yttrium have long been used as attractive dopants in ZnO-based systems because they enable the tuning of optical emission, engineering of oxygen vacancies, formation of heterojunctions, and enhancement of redox activity. However, although many of these dopants are often regarded simply as “high performance additives,” they also represent a strategically vulnerable class of materials because of geopolitical concentration, dependence on separation and refining capacities, and market volatility. Although rare-earth elements differ in their relative abundance, crustal abundance and effective supply security are not equivalent concepts. The critical issue is whether refined products can be accessed at stable prices and in a manner resilient to geopolitical disruptions [6,7,8,9].

Boron-based doping strategies introduce a different line of discussion at this point. The incorporation of boron directly into the ZnO lattice at low concentrations is particularly attractive for regulating thin film conductivity, promoting grain refinement, and modifying photocatalytic surface behavior. In addition, B_4_C/ZnO composites and h-BN/ZnO interfaces can generate interface driven functionalities that cannot be adequately explained by conventional lattice doping alone. More importantly, for Türkiye, boron is not merely a chemical element; it represents a strategic domestic resource class in terms of supply security, export capacity, and public industrial coordination. Therefore, boron containing systems should be evaluated not only through technical parameters, but also in conjunction with national resource policy [10,11,12].

Two major gaps are evident in the existing literature. First, boron-based and rare-earth-based dopants have often been investigated in different matrices, under different synthesis temperatures, and with different application targets. As a result, the available comparisons remain indirect and fragmented. Second, studies explaining material performance and the literature on critical raw materials and strategic dependence rarely intersect. Consequently, a researcher may identify that a given dopant is beneficial at the laboratory scale, yet it remains unclear how defensible that dopant is in terms of supply security, which national industrial strategies it aligns with, or which material family should be prioritized by public funding mechanisms [5,13,14].

The originality of this article lies in its approach, which does not reduce dopant superiority to a single performance metric, but instead integrates structural and morphological findings with strategic raw material policy within the same analytical framework. ZnO was selected as the common matrix, and boron-based and rare-earth-based dopants were compared using the same set of evaluation criteria. Accordingly, dopant selection was assessed by considering phase purity, secondary-phase formation, agglomeration tendency, grain-size control, surface homogeneity, pore architecture, processability, reproducibility, and strategic raw material risk together. The central thesis of the study is that differences that may appear technically limited can lead to more decisive policy implications when evaluated in the context of critical raw materials.

This study is structured around three interconnected analytical levels. First, ZnO is used as a common interpretation matrix to separate lattice doping, heterophase composite formation, and interface engineering. Second, boron and rare-earth-element-containing pathways are evaluated through an integrated XRD/FE-SEM/EDX/XPS/Raman/FT-IR evidence chain instead of isolated performance claims. Third, the resulting structure-morphology-function relationships are correlated with critical raw material management, supply chain resilience, technology readiness potential, and public R&D prioritization. This structure allows additive selection to be discussed simultaneously as a defect and interface engineering problem and a strategic resource allocation problem.

## 2. Scope of the Study, Research Questions, and Review Methodology

### 2.1. Literature Search, DOI Verification, and Selection Protocol

In constructing the reference pool, the primary criterion was to establish a core body of literature consisting of bibliographically verifiable and peer-reviewed publications. Accordingly, only records for which author information, article title, journal details, and DOI metadata could be consistently cross-validated were included in the study. For boron-based systems, publication clusters such as “B-doped ZnO,” “B_4_C/ZnO,” and “h-BN/ZnO” were considered. For rare-earth-based systems, studies addressing “Ce-doped ZnO,” “La-doped ZnO,” “Y-doped ZnO,” “ZnO/CeO_2_,” and related material architectures were evaluated. In the policy-oriented dimension, publications and reports focusing on critical raw materials, supply security, rare-earth supply chains, and the boron economy were examined in parallel.

In the second stage, a thematic relevance screening was conducted to ensure that the selected studies directly supported the analytical scope of the study. For the structural and morphological comparison, only studies that explicitly discussed dopant-induced effects on crystal structure, surface morphology, phase evolution, elemental distribution, or defect chemistry were retained. Studies that merely reported performance metrics without clarifying dopant incorporation, chemical state, spatial distribution, or host–dopant interaction were excluded. Likewise, records that conflated different host matrices or discussed multiple material systems without a clear ZnO-centered interpretation were not included in the core comparison set. For the policy dimension, official institutional reports and DOI-indexed academic publications were evaluated together, enabling technical evidence and governance-oriented evidence to be integrated within a unified review framework.

### 2.2. Analytical Unit of Comparison and Interpretive Framework

In this study, the analytical unit of comparison is defined according to the principle of evaluating the same dopant role within the same host matrix. Accordingly, direct lattice doping, controlled secondary-phase or composite formation, and interface/support engineering are treated as distinct material-design strategies rather than being merged under a single generalized “doping” category. This distinction is essential because the absence of such a framework may lead to category errors that are frequently encountered in the literature. For instance, the improved charge separation or interfacial transport enabled by a composite heterojunction may be incorrectly attributed to the success of conventional lattice doping, although the underlying mechanism is fundamentally different.

The present work is not designed as a quantitative meta-analysis, but rather as a structured critical review. This methodological choice is deliberate. Studies on boron- and rare-earth-based dopant systems have been reported using highly diverse synthesis routes, dopant concentrations, thermal treatment conditions, characterization depths, and application targets. Therefore, extracting a single numerical performance parameter from these heterogeneous studies and statistically combining the results would risk producing methodologically misleading conclusions. Instead, this study brings together DOI-verified peer-reviewed publications and official policy reports within a common interpretive framework, allowing the discussion to be organized around comparable structural, morphological, chemical, and policy-related variables. This approach is particularly suitable for developing mechanistic and policy-oriented inferences. However, it is explicitly acknowledged that the conclusions derived from this synthesis should not be interpreted as evidence of absolute material superiority under identical experimental conditions. Such meta-analytical equivalence would require a level of experimental standardization that remains largely absent from the ZnO dopant literature [1,13,15].

For this reason, all cross-study comparisons in the study are treated as conditional and mechanism-oriented rather than as directly normalized numerical rankings. Where the compared studies differ in matrix chemistry, synthesis route, dopant loading unit, annealing schedule, or application test protocol, the text interprets recurring trends only after separating the role of host matrix, additive family, interface geometry, and characterization depth. Direct horizontal performance comparison is therefore restricted to cases in which the same measurement anchor and the same relevant normalization condition can be stated explicitly.

To make this caution operational, the study applies a five-level evidence hierarchy: (i) nominal additive introduction reported by precursor loading; (ii) phase-level or spatial presence supported by XRD and EDX mapping; (iii) chemical-state or bonding evidence from XPS, Raman, and FT-IR; (iv) lattice-, defect-, or interface-level involvement supported by coherent peak shifts, microstrain, vibrational perturbation, or surface-state evolution; and (v) structure–property causality supported by a consistent link between characterization and application or dielectric response. Claims are formulated only at the highest evidential level supported by the available data. Thus, nominal addition is not treated as proof of lattice doping, and high functional output is not treated as proof of a specific mechanism unless the characterization chain supports that mechanism.

Five criteria were used to define the scope of the review. The first criterion was the use of ZnO as the common host matrix, since this enables boron- and rare-earth-based dopant families to be discussed within the same crystal-structure and defect-chemistry framework. The second criterion was the restriction of dopant categories to direct B doping, B_4_C/ZnO, and h-BN/ZnO on the boron side, and to Ce/CeO_2_, La/La_2_O_3_, and Y/Y_2_O_3_ systems on the rare-earth side. The third criterion was the availability of XRD, FE-SEM, EDX, XPS, Raman, and/or FT-IR data to support structural, chemical, and morphological interpretation. The fourth criterion was that the selected studies should provide not only performance-related outcomes, but also an explicit interpretation of how the dopant or secondary phase affects the crystal structure, surface morphology, defect chemistry, or interfacial behavior of the material. The fifth criterion was the inclusion, for the policy dimension, of DOI-indexed academic sources and official institutional reports addressing critical raw materials, supply chains, raw material security, rare-earth dependency, or the boron economy.

Within this framework, the article is organized around four research axes. The first axis examines how boron-based and rare-earth-based dopant strategies differ in terms of phase purity, lattice distortion, and secondary-phase formation within the same ZnO matrix. The second axis evaluates which dopant family provides a broader processing window with respect to agglomeration tendency, grain-size evolution, surface homogeneity, and morphological controllability. The third axis discusses the extent to which characterization techniques such as XRD, EDX, XPS, Raman spectroscopy, and FT-IR can reveal the actual incorporation mode, chemical state, and spatial distribution of dopants, while also identifying the methodological limits beyond which overinterpretation may occur. The fourth axis connects these technical distinctions to critical raw material policy by examining under which conditions public R&D incentives should prioritize a given material family.

In conducting the comparison, the selected studies were not assigned equal interpretive weight. Greater analytical value was given to studies that employed a clearly defined common matrix, provided a comparable characterization set, explicitly discussed the structural or chemical role of the dopant, and related the observed material response to synthesis conditions. By contrast, studies that only reported application-level performance, or attributed performance changes to dopant effects without demonstrating dopant incorporation, chemical state, or spatial distribution, were interpreted more cautiously. This distinction is particularly important for low atomic number elements such as boron, for which EDX analysis alone cannot provide definitive evidence of lattice incorporation. Therefore, results not supported by complementary techniques such as XPS, Raman spectroscopy, or FT-IR were considered within a limited interpretive scope. Similarly, in rare-earth oxide systems, assigning complete lattice solubility solely on the basis of XRD peak shifts was regarded as methodologically insufficient, particularly when secondary-phase formation, surface segregation, or interfacial oxide domains may also contribute to the observed diffraction response.

A central implication of this interpretive strategy is the systematic questioning of linear assumptions such as “material performance increases proportionally with dopant concentration.” In the ZnO-based literature, both boron- and rare-earth-containing systems may exhibit beneficial effects at low or intermediate dopant levels, whereas performance deterioration can occur at higher concentrations due to trap-state formation, phase segregation, surface clustering, excessive defect generation, or carrier scattering. For this reason, dopant strategies are evaluated throughout the study in conditional rather than absolute terms. The same caution is maintained in the policy-oriented discussion. Although rare-earth-based dopants are associated with strategic supply vulnerability, it is also recognized that, for certain advanced functional applications, the specific electronic, optical, catalytic, or defect-modulating roles provided by rare-earth elements may not be immediately replaceable by boron-based domestic alternatives.

Accordingly, this study performs two complementary tasks. On the one hand, it organizes laboratory-scale structural, chemical, and morphological findings within a comparative materials-science framework. On the other hand, it examines how these findings should be interpreted from the perspective of materials governance, supply-chain resilience, and public R&D prioritization. In this way, the article moves beyond a conventional literature review and proposes a preliminary decision-oriented framework that can be translated into future experimental research designs.

In addition to the literature backbone, seven primary experimental studies developed within the same problem domain were considered as a validation-oriented study set: ZnO–TiC [16], ZnO–B_4_C [17], ZnO–h-BN [18], ZnO–CNT/TiB_2_ [19], NiO–Y_2_O_3_ [20], NiO–V_2_O_5_ [21], and NiO–TiN [22]. These studies are not treated as a fully normalized head-to-head experimental campaign because they include different matrices, loading descriptors, additive geometries, thermal histories, and impedance-testing windows. Their role is therefore narrower and methodologically explicit: they provide a shared characterization language for interpreting phase response, lattice strain, particle/interface topology, chemical-state evolution, and low-frequency polarization mechanisms. Accordingly, the primary set is used for mechanism-level triangulation, while strict quantitative ranking is restricted to data points for which the matrix, frequency anchor, loading basis, and measurement conditions are disclosed.

The scope, inclusion criteria, and comparison logic adopted in this review are summarized in Table 1.

## 3. Rationale for Selecting ZnO as the Common Matrix

The selection of ZnO as the common comparison matrix is not arbitrary. ZnO, which possesses a wurtzite crystal structure, has generated an exceptionally broad research literature in both fundamental and applied materials science, owing to its band gap of approximately 3.3 eV and high exciton binding energy. Its ability to be synthesized in various morphologies, including thin films, nanorods, nanoparticles, flower-like microstructures, porous networks, and hybrid heterostructures, provides a suitable platform for revealing the microstructural consequences of dopant strategies. The high sensitivity of ZnO to surface and bulk defects makes it possible to interpret not only the electrical or optical effects of dopants, but also their crystallographic and morphological impacts [1,2,3].

A second important advantage of the ZnO literature is the availability of a sufficiently dense publication base for both boron and rare-earth-based dopant families. B-doped thin films have been extensively investigated for transparent conducting applications and sensors, whereas composite and interfacial structures containing B_4_C and h-BN have gained prominence particularly in the context of photocatalysis and surface engineering. Similarly, Ce-, La-, and Y-doped ZnO systems have been widely reported for gas sensing, photoluminescence, photocatalysis, thin film transistors, and solar energy related applications. This breadth of literature makes the comparison of heterogeneous dopant strategies on the same matrix more defensible for ZnO than for many other oxide systems [15,23,24].

The defect chemistry of ZnO is particularly instructive for distinguishing between different dopant strategies. Oxygen vacancies, zinc interstitials, surface adsorbates, and grain-boundary states directly influence sensor response, photoluminescence, and photocatalytic activity. When a dopant strategy regulates these defect domains in a controlled manner, its effect can often be observed simultaneously across multiple characterization techniques. For example, crystallite size and peak position in XRD, agglomeration behavior and grain-boundary architecture in FE-SEM, O 1s components in XPS, defect-related modes in Raman spectra, and surface bonding features in FT-IR can be interpreted together. Thus, ZnO functions not only as a functional material, but also as an analytical platform for resolving dopant mechanisms [25,26,27].

Moreover, the synthetic versatility of ZnO is consistent with the aims of this article. Similar fundamental structural questions can be addressed using markedly different techniques, including solgel processing, hydrothermal synthesis, chemical bath deposition, aerosol assisted CVD, reactive sputtering, and spray pyrolysis. This means that the effects of dopant strategies are not limited to a single synthesis route. At the same time, however, it should be recognized that the synthesis pathway strongly determines the final material response. For this reason, results obtained from different synthesis routes for the same dopant type were compared at the mechanistic level, whereas absolute performance values were not directly compared. In this way, ZnO was used as a “common ground” that enables comparison while also requiring methodological caution.

Another advantage of ZnO is that the diversity of its application areas can be naturally connected to the materials policy discussion. Fields such as transparent conducting films, environmental sensors, photocatalytic water treatment, UV photonics, biomedical surfaces, and energy devices require different dopant strategies to be evaluated not only within the laboratory context, but also in terms of potential supply chains and industrial scalability. In other words, a comparison built on ZnO establishes a direct bridge between the scientific impact of a dopant strategy and its potential for industrial implementation. This feature makes it possible to discuss the public policy dimension of the article without detaching it from the technical literature. The visibility of ZnO in biomedical and antimicrobial surface applications further indicates that dopant selection should be assessed not only in terms of functionality, but also with respect to safety, processing conditions, and application context [23,24,25,26,27,28].

For these reasons, ZnO is not treated in this study merely as a single “model material,” but rather as the methodological backbone of comparative reasoning. The selection of a common matrix makes it possible to examine the effects of boron- and rare-earth-based dopants while minimizing deviations arising from the intrinsic differences among distinct oxide systems. Thus, the conclusions reached here are relevant not only to ZnO, but also to a broader conceptual proposal regarding how dopant strategies should be classified.

### ZnO Defect Chemistry and Doping Sensitivity

The pronounced sensitivity of ZnO to dopant engineering originates from its intrinsically active and multi-level defect chemistry. Oxygen vacancies, zinc interstitials, surface hydroxyl groups, chemisorbed oxygen species, and grain-boundary-induced potential barriers collectively govern the optical, electrical, dielectric, catalytic, and surface-reactive behavior of ZnO. Therefore, doping should not be interpreted merely as the incorporation of an additional element into the host lattice. Rather, it represents a deliberate modification of the pre-existing defect landscape. One of the fundamental distinctions between boron- and rare-earth-based strategies emerges precisely at this point. Boron-containing dopants generally modulate the defect structure in a more gradual and structurally less disruptive manner, whereas rare-earth elements tend to introduce stronger ionic, electronic, and chemical perturbations, often leading to defect clustering, interfacial heterogeneity, or secondary-phase-assisted functionality [2,25].

The role of oxygen vacancies in ZnO remains a central and debated issue in the literature. In certain applications, oxygen vacancies may function as active sites that enhance gas-sensing response, adsorption capacity, photocatalytic activity, or surface redox reactions. In other cases, however, they may act as deep-level trap centers that promote carrier recombination and deteriorate charge-transport efficiency. Consequently, the linear assumption that a higher oxygen-vacancy concentration necessarily results in improved material performance is not generally valid. Redox-active rare-earth dopants such as Ce can strongly influence oxygen-vacancy formation, migration, and stabilization, thereby offering significant advantages in selected catalytic, sensing, or photoactive systems. By contrast, boron-based dopants may tune the defect balance with comparatively lower lattice distortion, which can provide a more stable and homogeneous response, particularly in thin-film architectures, nanostructured ZnO, or systems requiring uniform defect distribution. This distinction explains why rare-earth dopants may be more rational for applications requiring strong redox activity or defect enrichment, whereas boron-based strategies may be more suitable when structural continuity, morphological uniformity, and controlled defect modulation are prioritized.

Grain-boundary behavior is another critical factor controlling dopant effectiveness in ZnO. In nanostructured ZnO, grain boundaries are not passive geometric interfaces; rather, they function as electrochemically and electronically active regions that regulate charge transport, surface reactions, local band bending, and intergranular potential barriers. Boron incorporation or boron-containing secondary phases often tend to restrict grain-boundary mobility, contributing to refined grains, suppressed abnormal growth, and more uniform microstructural development. Rare-earth elements such as La and Ce, however, may preferentially segregate near grain boundaries or interfacial regions. This can suppress grain growth and increase surface activity, but may simultaneously generate local compositional heterogeneity, enhanced carrier scattering, or agglomeration at higher dopant levels. This dual effect explains why rare-earth doping can appear both beneficial and potentially destabilizing depending on concentration, synthesis conditions, and target functionality. In such cases, functional activity may be enhanced in specific regions of the material, while transport losses or morphological non-uniformity may become more pronounced elsewhere.

From the perspective of surface chemistry, boron- and rare-earth-based dopant families generate functional value through different mechanisms. Boron-based dopants, particularly h-BN-containing interfaces, can regulate surface energy, nucleation behavior, particle arrangement, and film continuity, thereby supporting more homogeneous microstructures or controlled interfacial architectures. Rare-earth oxides such as CeO_2_ and La_2_O_3_, in contrast, can modify surface reactivity, adsorption behavior, oxygen exchange capability, and catalytic cycles more strongly. Thus, the concept of “surface improvement” is applicable to both dopant families, but its mechanistic meaning differs substantially. In boron-based systems, improvement is primarily associated with morphological ordering, interfacial stability, and controlled defect modulation. In rare-earth-containing systems, it is more often governed by chemical activity, redox flexibility, oxygen-vacancy enrichment, and surface-reactive heterogeneity.

This defect-centered interpretation supports one of the central arguments of the present study: the success of a dopant strategy is determined not only by the chemical identity of the dopant, but also by the manner in which it interacts with the initial defect structure of ZnO. The same dopant may produce markedly different outcomes depending on precursor chemistry, synthesis route, calcination temperature, atmosphere, particle-growth regime, and post-treatment history. Therefore, apparent contradictions in the literature should not automatically be interpreted as evidence that a given dopant is intrinsically effective or ineffective. Rather, such inconsistencies often reflect differences in the initial ZnO matrix, defect population, crystallization pathway, and processing history. This point also highlights why future directly comparative experimental studies must be designed using identical precursor chemistry, dopant introduction routes, thermal histories, and characterization protocols.

The main scientific reasons supporting the selection of ZnO as the common comparison matrix are summarized in Table 2.

## 4. Conceptualization of Dopant Strategy: Doping, Composite Formation, and Interface Engineering

In the present study, the term “dopant strategy” is used deliberately, because structurally and mechanistically distinct material interventions are frequently grouped under the generic term “doping” in the ZnO literature. However, incorporating boron into the ZnO lattice, forming a ZnO–B_4_C composite, and growing ZnO on h-BN do not represent the same type of material chemistry. The first case refers primarily to limited atomic incorporation, defect regulation, and lattice-level modification. The second relies on the presence of a secondary phase, interfacial contact, and phase-boundary-mediated functionality. The third generally corresponds to interface or support engineering, often involving a two-dimensional scaffold or van der Waals-type heterointerface. A comparable complexity is also observed in rare-earth-containing ZnO systems. Elements such as Ce, La, and Y may be partially incorporated into the ZnO lattice, segregate at the surface, form oxide clusters, or behave as separate phases that establish heterojunctions with ZnO. Therefore, the first requirement for a scientifically rigorous comparison is to distinguish these mechanisms from one another rather than treating them as equivalent forms of doping [29,30,31,32].

The first level of a dopant strategy is direct lattice doping. In this category, the objective is to alter carrier concentration, defect equilibrium, local lattice strain, or electronic structure by introducing a limited number of foreign species into the crystal lattice of the host matrix. B-doped ZnO thin films are typical examples of this class. However, because boron is a small and light element, its incorporation into ZnO should not be evaluated solely through the assumption of substitutional occupation. Interstitial positioning, defect-associated complexes, boron–oxygen interactions, or local structural distortions may also contribute to the observed material response. For this reason, a simple XRD peak shift is not sufficient to determine the actual atomic position or chemical role of boron. A similar caution is required for Y- or Ce-doped ZnO nanoparticles, where it is necessary to distinguish whether the observed structural modification arises from true lattice substitution, surface segregation, defect compensation, or the formation of nanoscale oxide-rich domains [33,34,35,36].

The second level is the controlled secondary-phase or composite strategy. Material systems such as B_4_C/ZnO, ZnO/CeO_2_, and ZnO/La_2_O_3_ can be evaluated within this category. In these structures, the primary objective is not necessarily atomic dissolution within the ZnO lattice, but the generation of interphase charge transfer, active surface area, redox coupling, photoinduced charge separation, or enhanced interfacial functionality between two distinct phases. Therefore, the presence of a secondary phase should not automatically be interpreted as evidence of failed doping or undesired impurity formation. In many cases, the secondary phase is the core design element of the material’s architecture. The critical questions are instead whether the secondary phase is homogeneously distributed, whether it forms isolated clusters or continuous interfacial domains, what length scale governs phase interaction, and what type of electronic or chemical interface is established with the ZnO matrix. Accordingly, in composite-type systems, “phase purity” should be assessed as a target-dependent criterion rather than as an absolute requirement [37,38,39].

The third level is interface and support engineering. h-BN/ZnO systems constitute one of the clearest examples of this category. h-BN offers a chemically stable two-dimensional structure, high thermal conductivity, strong oxidation resistance, and, under suitable synthesis conditions, a surface-guiding function for nucleation and growth. The growth of ZnO on h-BN, or the formation of intimate ZnO/h-BN interfaces, can influence crystal nucleation, particle arrangement, surface energy, defect distribution, and charge-transport pathways in ways that cannot be reduced to the simple addition of a secondary phase. For this reason, h-BN-containing ZnO systems should not be interpreted as conventional doping systems, but rather as examples of interface design and heterostructure engineering. The same conceptual logic applies to certain rare-earth-oxide-modified ZnO systems, particularly when CeO_2_, La_2_O_3_, or related oxides form surface heterojunctions or interfacial redox-active domains [29,40,41].

When this conceptual distinction is not maintained, at least three recurring interpretive errors emerge in the literature. First, secondary-phase formation is often automatically classified as an undesired impurity, although in many photocatalytic, sensing, and interfacial charge-transfer systems the heterojunction itself may be the primary source of improved performance. Second, low-dose lattice doping and composite incorporation are sometimes evaluated along the same mechanistic axis, leading to overly broad and potentially incorrect generalizations about dopant effectiveness. Third, limited evidence obtained from a single technique, such as EDX or XRD, is occasionally used to support excessive claims regarding the complete mechanism of dopant action. The central approach of this review is therefore to classify dopant strategies according to their dominant material-design mechanism before evaluating each class within its own structural, chemical, and functional expectations.

This conceptual distinction is equally important for policy interpretation. From the perspective of public R&D support and strategic material prioritization, the decisive criterion is not merely whether a dopant improves a laboratory-scale performance metric. It is also necessary to evaluate the synthesis regime under which improvement is achieved, the interface architecture required to generate functionality, the scalability of the process, and the supply risk associated with the dopant or secondary phase. Direct lattice doping with a low-cost, domestically accessible element and the construction of a heterostructure using an imported rare-earth oxide cannot be treated as equivalent strategies in terms of industrial scalability, supply-chain exposure, and technological sovereignty. Therefore, accurate classification of the underlying material chemistry constitutes an essential starting point for both scientific interpretation and strategic prioritization.

**Figure 1:**
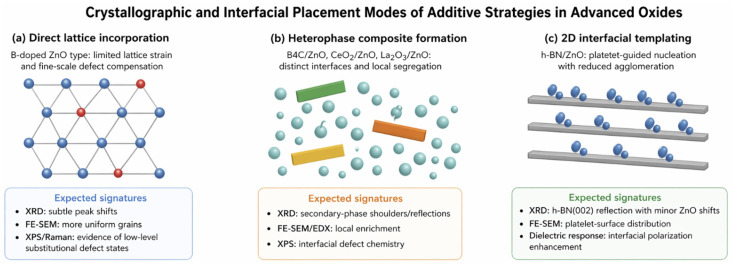
Distinct crystallographic and interfacial incorporation modes generated in advanced oxides by direct lattice doping, heterophase composite formation, and two-dimensional interface guidance.

The crystallographic incorporation modes associated with different dopant strategies, together with their distinguishing characterization signatures, are schematically illustrated in Figure 1 [29,33,34,35,36,37,38,39,40,41,42,43,44,45].

## 5. Boron-Based Dopant Strategies

### 5.1. Direct B Doping and Defect Tuning

The most important characteristic of boron-based ZnO strategies is that they do not operate through a single chemical or structural mode. Although directly B-doped ZnO thin films, B_4_C/ZnO composites, and h-BN/ZnO interfacial architectures can all be placed within the broader boron-based material family, their mechanisms of action are fundamentally different. In direct B doping, the primary expectation is the regulation of crystal defects, carrier concentration, local lattice distortion, and grain-growth behavior through the small atomic radius and valence-related effects of boron. In B_4_C-containing composites, by contrast, the functional response is additionally shaped by the ceramic secondary phase, carbon-related contributions, and the interfacial contact established between B_4_C and ZnO. In h-BN/ZnO systems, the dominant effects are more closely associated with two-dimensional support behavior, growth guidance, interfacial charge transport, and surface stabilization. Therefore, grouping all boron-containing ZnO systems under a single heading such as “boron doping” is methodologically insufficient and may obscure the actual mechanism responsible for the observed material response [26,30,33,35]. Solution-growth studies on ZnO:B microflower structures further demonstrate that boron can influence not only the electronic state of ZnO, but also nucleation behavior, growth directionality, and crystal-growth topology [46].

Studies on directly B-doped ZnO thin films and nanoparticles indicate that a delicate balance exists between crystallinity, defect density, carrier behavior, and functional performance, particularly at low and intermediate dopant concentrations. Hurma’s work on nanostructured ZnO films demonstrated that increasing boron concentration simultaneously modifies crystal structure, optical response, and electrical properties, but that these changes do not follow a simple linear trend. Similarly, the studies of Tsay and Hsu, as well as Ilican and co-workers, showed that pH, precursor chemistry, dopant concentration, and processing conditions strongly affect film morphology, crystallization quality, and optical/electrical behavior in sol–gel-derived B-doped ZnO films. The common conclusion emerging from this literature is that boron acts as a low-concentration but high-impact regulating dopant. When the optimum concentration range is exceeded, structural disorder, increased trap-state density, reduced crystallinity, or optical deterioration may occur [33,34,47].

B-doped ZnO has gained particular importance in thin-film and transparent conducting applications because, under carefully controlled synthesis conditions, boron can tune crystal growth and carrier transport without completely disrupting the ZnO framework. The simultaneous improvement of optical transparency and electrical conductivity in B-doped ZnO films prepared by aerosol-assisted chemical vapor deposition strengthens the technological relevance and transfer potential of this dopant strategy. Similarly, B-doped ZnO films fabricated by reactive sputtering or pulsed DC magnetron sputtering have shown that boron incorporation can regulate carrier concentration, mobility, and optoelectronic performance. However, the study by Chiu and Chiang on amorphous B-doped ZnO films also emphasizes that uncontrolled boron incorporation may complicate trap-level formation and the electronic defect landscape. Thus, boron should not be regarded as a structurally harmless dopant that automatically preserves crystal stability. Rather, it is a powerful defect- and carrier-tuning element whose effectiveness depends strongly on concentration control, synthesis route, and post-treatment conditions [48,49,50,51].

One of the most frequently reported morphological effects of boron doping is the suppression of excessive grain growth and the development of a more uniform microstructure. In several studies based on sol–gel, hydrothermal, and related solution routes, suitable boron contents have been associated with more homogeneous surfaces, reduced agglomeration tendency, and a narrower crystallite-size distribution. These observations should not be interpreted as evidence that boron alone acts as an independent grain refiner, since synthesis temperature, aging time, solvent chemistry, precursor reactivity, and annealing atmosphere also exert decisive influence on microstructural evolution. Nevertheless, the broader literature suggests that boron-based direct doping generally produces less severe phase segregation and confines morphological heterogeneity within a narrower range, at least when compared with many rare-earth-based dopant systems [52,53,54].

### 5.2. B_4_C/ZnO Composites

The second major boron-based pathway is represented by B_4_C/ZnO composites. In these systems, B_4_C should not be interpreted as a species atomically dissolved within the ZnO lattice, but rather as a heterophase component that contributes through interfacial contact, secondary-phase functionality, and composite-level architecture. Studies focusing on photocatalytic applications have reported that B_4_C incorporation can promote charge-carrier separation, enhance surface activity, and, under suitable synthesis conditions, provide a more stable reaction environment depending on the spatial arrangement of the composite phases. However, the evaluation criterion for B_4_C/ZnO systems should not be limited to whether a single ZnO phase is observed in XRD. Instead, the key questions are how homogeneously the B_4_C phase is dispersed, whether it forms isolated agglomerates or effective interfacial domains, what type of contact it establishes with ZnO grains, and how the composite architecture modifies surface topology and charge-transfer pathways. For this reason, the B_4_C/ZnO strategy should be assessed not within the logic of conventional lattice doping, but within the framework of controlled composite engineering [38].

### 5.3. h-BN/ZnO Interfaces and Directed Growth

h-BN/ZnO structures represent one of the most sophisticated examples of boron-based ZnO strategies. The two-dimensional morphology, chemical stability, oxidation resistance, and surface-guiding capability of h-BN can substantially influence the nucleation, alignment, and crystal-development behavior of ZnO. The van der Waals epitaxy approach reported by Oh et al. showed that h-BN is not merely an inert support, but can function as an active platform for architectured ZnO nanostructures. More recent studies have similarly demonstrated that ZnO-based hybrids containing boron nitride quantum dots, h-BN, or p-BN can improve photocatalytic degradation efficiency, luminescence behavior, interfacial bonding, and surface reactivity. In particular, the ability of h-BN-supported architectures to suppress agglomeration, stabilize particle distribution, guide heterogeneous nucleation, and facilitate charge transfer at surface interfaces constitutes a major advantage that distinguishes this strategy from conventional rare-earth lattice doping [29,40,41,42].

### 5.4. Critical Interpretation of the Boron-Based Family

A central issue in the interpretation of boron-based ZnO strategies is that the reliable detection and localization of boron are analytically more challenging than those of rare-earth elements. EDX has inherently limited sensitivity for low-atomic-number elements such as boron. Therefore, claims regarding homogeneous boron incorporation or uniform boron distribution cannot be considered sufficiently supported when they rely solely on EDX mapping. A robust interpretation requires the combined evaluation of the B 1s XPS signal, B–O- or B–N-related vibrational features in Raman or FT-IR spectra, and the corresponding structural evidence obtained from XRD and morphological analyses. This analytical difficulty should not be regarded as a weakness specific to boron-based studies. On the contrary, it represents a methodological warning that boron-containing ZnO systems require a more disciplined and multi-technique characterization strategy. In rare-earth-doped systems, elemental detection is generally more straightforward because of the higher atomic numbers of Ce, La, or Y. However, in these systems, the main interpretive challenge shifts from elemental detection to the determination of incorporation mode. In other words, it becomes necessary to distinguish whether the rare-earth element is incorporated into the ZnO lattice, segregated at the surface, accumulated at grain boundaries, or present as an oxide-rich secondary phase. Thus, the main analytical challenge in boron-based systems is detection sensitivity, whereas the primary challenge in rare-earth-based systems is identifying the structural and chemical mode of incorporation.

Accordingly, the term boron-based strategy is used here as an umbrella category and not as automatic proof of substitutional B incorporation in ZnO. Operationally, boron-related evidence is interpreted through four safeguards. First, EDX is used only as evidence of nominal elemental presence or phase-level distribution because low-Z boron is close to the practical detection limit and is sensitive to matrix effects, detector window conditions, and background subtraction. Second, B 1s XPS should be chemically resolved rather than simply detected, because B–C/B_4_C-like, B–N, and oxidized B–O or borate-like environments occupy different chemical-state regimes and imply different mechanisms. Third, Raman or FT-IR features assigned to B–N, B–O, B–B, or B–C vibrations must be consistent with the proposed material architecture; for example, h-BN/ZnO should be interpreted through B–N-related vibrational evidence and sheet/interface morphology, whereas B_4_C/ZnO should be interpreted through carbide-related bonding and heterophase contact. Fourth, any claim of lattice- or defect-level B interaction must be compatible with XRD peak position, microstrain, crystallite-size evolution, and morphology. Only when these signatures converge is lattice-/defect-level B involvement discussed; otherwise, B_4_C/ZnO and h-BN/ZnO are treated primarily as heterophase or interface-engineered systems.

When considered collectively, boron-based ZnO strategies exhibit three important characteristics. First, direct B doping generally offers a comparatively moderate modification pathway that can preserve the dominant ZnO phase while enabling controlled regulation of defect chemistry, crystallite size, grain growth, and microstructural uniformity. Second, boron-containing secondary phases such as B_4_C and h-BN extend the concept of dopant strategy beyond conventional lattice substitution by generating functional improvements through composite formation, interfacial contact, directed growth, and heterostructure engineering. Third, and most importantly from a policy perspective, boron-based material pathways may provide a shorter, more secure, and strategically favorable route from laboratory-scale development to industrial implementation in countries with domestic boron resource advantages, such as Türkiye. However, this strategic advantage should not be interpreted as evidence of universal technical superiority. In certain optical, catalytic, sensing, or redox-mediated applications, the specific electronic and defect-modulating functions provided by rare-earth elements may not be readily replaceable by boron-based alternatives. Therefore, the value of boron-based ZnO strategies should be assessed through a dual framework that considers both technical performance and resource governance.

Selected representative studies, together with the main structural and morphological inferences associated with boron-based ZnO strategies, are summarized in Table 3.

## 6. Rare-Earth-Based Dopant Strategies

### 6.1. General Mechanism: Ionic Size, Redox Behavior, and Segregation

Rare-earth-based ZnO strategies involve dopants that are generally more readily detectable than boron-based dopants because of their higher atomic numbers and stronger elemental contrast in conventional characterization techniques. However, this analytical visibility is often accompanied by more complex structural and chemical outcomes. The relatively large ionic radii, variable valence states, and strong oxide-forming tendencies of rare-earth elements such as Ce, La, and Y increase the probability of lattice strain, defect clustering, surface segregation, and secondary-phase formation during their interaction with ZnO. At the same time, these features provide a powerful functional toolset for tailoring oxygen-vacancy concentration, enhancing redox activity, extending optical absorption into the visible region, and generating surface-active heterojunctions. Therefore, rare-earth dopants often produce functional intensity rather than structural simplicity. In this sense, they should be regarded as more complex, but potentially more powerful, material interventions within the ZnO dopant landscape [4,31,32].

### 6.2. Ce- and CeO_2_-Based Strategies

Cerium-based dopants represent one of the most functionally significant subgroups within the rare-earth family. The Ce^3+^/Ce^4+^ redox couple can strongly influence oxygen storage and release behavior, surface adsorption, oxygen-vacancy dynamics, and charge-carrier separation in Ce-containing ZnO systems. Studies on Ce-doped ZnO nanorods, nanoparticles, and related architectures frequently report enhanced visible emission, improved photocatalytic degradation, and increased gas-sensing response toward selected analytes. However, these effects are often not attributable to direct lattice doping alone. In many cases, the functional improvement is more accurately associated with Ce-rich surface species, mixed Ce oxidation states, oxygen-defect modulation, or the formation of ZnO/CeO_2_ heterojunctions. Therefore, the effectiveness of Ce-based modification depends not only on nominal Ce concentration, but also on Ce oxidation state, phase distribution, local chemical environment, and spatial positioning within or on the surface of the ZnO matrix [35,43,55,56].

CeO_2_-containing ZnO composite and heterophase systems have been widely investigated, particularly in photocatalysis and gas-sensing studies. In ZnO/CeO_2_ architectures, heterojunction formation may enhance charge separation, while the oxygen storage capacity and surface redox-cycling ability of CeO_2_ can facilitate the generation of reactive oxygen species. Studies focusing on H_2_S sensing, dye degradation, and bio-related cellular interactions indicate that CeO_2_ incorporation can provide ZnO with a more chemically active and reactive surface environment. However, this technical advantage is accompanied by an increased risk of phase complexity. CeO_2_ clustering, excessive surface heterogeneity, and uncontrolled phase segregation may reduce interfacial effectiveness and even reverse the desired performance gain, particularly at high dopant or secondary-phase loadings. Consequently, Ce-based strategies offer strong functional potential, but their synthesis and composition windows are generally narrower than those of more structurally moderate boron-based dopant systems [38,57,58,59].

### 6.3. La- and La_2_O_3_-Based Strategies

Lanthanum-based dopants are generally less redox-active than cerium, yet they can exert pronounced effects on grain-boundary behavior, crystallite evolution, surface chemistry, and interfacial reactivity in ZnO. The literature on La-doped ZnO covers a broad range of applications, including photocatalysis, antibacterial activity, optical absorption, and microwave-related properties. Because of the relatively large ionic radius of La^3+^ and its limited solubility in the ZnO lattice, La incorporation increases the likelihood of lattice strain, grain-boundary segregation, and secondary-phase formation. In some studies, this behavior is reflected positively through reduced crystallite size, modified surface area, or enhanced surface activity. In other cases, however, it appears as surface clustering, phase heterogeneity, and reduced morphological uniformity. Therefore, La doping should not be framed as a one-directional improvement strategy, but rather as a conditional optimization problem governed strongly by synthesis route, pH, annealing temperature, precursor chemistry, and morphology control [44,60,61].

In ZnO composites formed with La_2_O_3_, surface basicity, interfacial contact, and heterojunction formation become more dominant than direct lattice substitution. Studies focused on the degradation of organic pollutants have reported that La_2_O_3_-containing ZnO composites can enhance active surface area, improve charge separation, and modify adsorption behavior. However, two aspects are critical for the technical evaluation of such systems. The first is the distribution scale of the La_2_O_3_ phase; finely dispersed interfacial domains are more desirable than large heterogeneous clusters because they provide greater effective contact area and more controlled charge-transfer pathways. The second is the reproducibility of surface morphology, since many La-containing ZnO systems are highly sensitive to pH, hydrolysis kinetics, calcination conditions, and annealing atmosphere. This sensitivity requires particular caution when translating laboratory-scale performance into scalable material processing or industrial implementation [39,62,63].

### 6.4. Y- and Y_2_O_3_-Based Strategies

Yttrium-based dopants exhibit a comparatively more balanced structural profile within the rare-earth family in selected ZnO studies. Investigations on Y-doped ZnO nanoparticles and thin films have reported enhanced UV emission, improved gas-sensing response, better thin-film transistor behavior, and increased carrier mobility in solar-energy-related architectures. Although Y^3+^ interaction with ZnO can generate more controlled textural and electronic modifications than those frequently observed in Ce- or La-based systems, the possibility of secondary-phase formation, lattice distortion, and microstructural heterogeneity at higher loadings cannot be overlooked. In particular, spray pyrolysis, sol–gel, and sputtering-based studies show that Y doping can substantially modify film orientation, crystallite size, surface morphology, and carrier behavior [36,45,64,65].

A notable feature of Y-based dopant strategies is that their effects are not limited to optical or sensing-related responses. Reports of increased carrier concentration and mobility in Y-doped ZnO nanorod arrays suggest that Y incorporation can, under suitable structural configurations, improve carrier transport rather than merely generate additional defect states. This indicates that rare-earth dopants should not be automatically associated with detrimental phase complexity or agglomeration in every case. Nevertheless, Y-based modification still requires more sensitive process control than the relatively moderate microstructural changes often observed in B-doped ZnO systems. The balance between beneficial electronic/structural modification and phase-related deterioration is established within a narrow composition, thermal-treatment, and morphology window [66,67].

### 6.5. General Structural Profile of the Rare-Earth Family

At a general level, rare-earth dopants can be described as offering functional strength and morphological risk within the same material-design pathway. Ce-based modification generates value primarily through oxygen-vacancy regulation, redox activity, and surface oxygen exchange. La-based modification is more closely associated with surface chemistry, grain-boundary control, and interfacial reactivity. Y-based modification contributes mainly through optical, electronic, and carrier-transport tuning. Despite these different functional roles, all three dopant families share a common structural feature: they impose a relatively strong chemical and crystallographic intervention on a comparatively simple oxide matrix such as ZnO. Consequently, even minor changes in dopant concentration, precursor chemistry, solution pH, annealing atmosphere, calcination temperature, or growth substrate can substantially alter phase composition, defect structure, and morphology. Therefore, the high-performance outcomes reported for rare-earth-modified ZnO systems should be evaluated together with the uncertainties they introduce in terms of reproducibility, processing tolerance, and scalability.

Another critical characteristic of rare-earth-based strategies is that elemental detection is comparatively straightforward, whereas mechanistic interpretation is considerably more complex. The presence of Ce, La, or Y can generally be confirmed more easily by EDX, XPS, or related elemental/chemical analyses than the presence of boron. However, confirming elemental presence does not by itself reveal whether these species are substitutionally incorporated into the ZnO lattice, segregated at the surface, accumulated at grain boundaries, or present as discrete oxide nanodomains. For this reason, small XRD peak shifts, peak broadening, or modest changes in crystallite size should not automatically be interpreted as definitive evidence of true lattice doping. This is particularly important when CeO_2_, La_2_O_3_, Y_2_O_3_, or related oxide phases exist at low concentration or in nanoscale/dispersed forms, where their diffraction signatures may be weak or partially masked by the dominant ZnO phase. Under such conditions, interfacial effects, surface segregation, or heterojunction formation may govern the functional response more strongly than lattice incorporation. Therefore, rare-earth-based ZnO systems also require cautious interpretation based on complementary structural, spectroscopic, morphological, and chemical-state analyses.

For rare-earth-containing ZnO systems, mechanism assignment is made through a decision sequence rather than by elemental detection alone. Partial lattice incorporation or solid-solution-like behavior is inferred only when lattice-parameter evolution is compositionally coherent, no discrete CeO_2_-, La_2_O_3_-, or Y_2_O_3_-related signature is resolved by XRD/Raman within the detection limits, and EDX/XPS do not indicate localized rare-earth enrichment. Surface or grain-boundary segregation is inferred when EDX maps, line scans, or surface-sensitive XPS show local enrichment without a clearly crystallized bulk secondary phase. Intentional or unintentional heterophase formation is inferred when rare-earth-oxide diffraction, Raman, chemical-state, or interfacial signatures are present. The functional mechanism is then assigned accordingly: Ce-containing systems are discussed mainly in relation to Ce^3+^/Ce^4+^ redox cycling and oxygen-vacancy exchange; La-containing systems in relation to surface basicity, grain-boundary modification, and limited solubility; and Y-containing systems in relation to strain, energy-level adjustment, and interface-controlled transport. This prevents the generic label “rare-earth doping” from masking the distinction between lattice doping, segregation, and heterostructure-driven performance.

Overall, rare-earth-based ZnO strategies generally represent more aggressive and function-oriented interventions than boron-based systems. This feature can provide substantial advantages in selected applications, particularly where redox cycling, visible-light response, surface adsorption, oxygen-vacancy modulation, or enhanced charge separation is required. However, these benefits are often accompanied by higher phase complexity, stronger agglomeration tendency, narrower processing windows, more demanding synthesis control, and a more challenging supply profile. Therefore, rare-earth dopants should not be discussed through a generalized discourse of “high performance.” Instead, they should be evaluated through the combined axes of functional indispensability, structural controllability, process manageability, and supply-chain vulnerability.

Selected representative studies, together with the main structural and morphological inferences associated with rare-earth-based ZnO strategies, are summarized in Table 4.

## 7. Integrated Interpretation of Characterization Techniques

One of the most common methodological errors in comparing dopant strategies is the overinterpretation or underinterpretation of what each characterization technique can actually demonstrate. XRD, FE-SEM, EDX, XPS, Raman spectroscopy, and FT-IR spectroscopy are complementary analytical tools, but they are not interchangeable. Each technique provides a different level of information regarding phase structure, morphology, elemental distribution, chemical state, bonding environment, or defect-related structural response. In the present study, each dopant mechanism is therefore interpreted, as far as possible, through multiple lines of evidence in order to improve the reliability of the comparison. A substantial part of the distinction between boron- and rare-earth-based ZnO systems can be understood not from individual techniques alone, but from the way these techniques are integrated within a coherent evidence framework.

XRD is a fundamental technique for identifying crystalline phases, diffraction peak shifts, peak broadening, preferred orientation, and crystallite-size trends. However, XRD alone cannot prove that a dopant has been incorporated into the ZnO lattice. In low-content boron-doped systems, only limited peak shifts may be observed, and the absence of pronounced diffraction changes should not automatically be interpreted as the absence of boron-related modification. Conversely, in Ce-, La-, or Y-modified ZnO systems, observable peak shifts may originate not only from true substitutional incorporation, but also from residual strain, crystallite-size variation, defect-induced lattice distortion, preferred orientation changes, or nanoscale secondary phases. Therefore, XRD findings, particularly in borderline cases, should not be overinterpreted unless they are supported by complementary chemical-state, elemental-distribution, and morphological evidence [1,2,35,64].

FE-SEM provides the most direct visual information on agglomeration behavior, surface homogeneity, grain morphology, particle-size distribution, porosity, and microstructural continuity. However, SEM images alone do not constitute definitive structural proof. The representativeness of the selected imaging area, sample preparation procedure, accelerating voltage, magnification level, and image selection criteria can all influence interpretation. Nevertheless, many of the practical morphological differences between boron- and rare-earth-based ZnO strategies are first recognized through FE-SEM observations. In h-BN-supported or boron-containing interfacial systems, more regular particle distribution, reduced clustering, and improved surface continuity are frequently reported. In contrast, rare-earth-oxide-modified ZnO systems may exhibit more pronounced surface heterogeneity, grain-boundary enrichment, or secondary-phase island formation, especially at higher dopant or oxide loadings. These observations become scientifically meaningful only when they are interpreted together with XRD, EDX, and chemical-state analyses.

EDX is a rapid and useful tool for elemental identification and spatial mapping, particularly for medium- and high-atomic-number elements. However, its reliability is limited for low-atomic-number elements such as boron. This limitation is especially important because a considerable part of the boron-based ZnO literature may overstate the evidential value of EDX data. The absence of a detectable boron signal at low dopant levels does not necessarily prove that boron is absent from the material. Conversely, a weak or localized signal does not confirm homogeneous lattice incorporation. In rare-earth-modified systems, EDX is generally more informative, since Ce, La, and Y can be more readily detected and their clustering, grain-boundary enrichment, or heterogeneous surface distribution can often be visualized. Nevertheless, EDX does not provide information on oxidation state, bonding environment, or lattice incorporation. It should therefore be regarded as an indicator of elemental presence and spatial distribution rather than as definitive evidence of dopant chemistry.

XPS is one of the most critical techniques for distinguishing dopant mechanisms in ZnO-based systems. For boron, the B 1s region is often weak and difficult to interpret at low loading levels. However, when a reliable signal is obtained, it can provide direct information on the chemical environment of boron, including B–O- or B–N-related bonding states. In Ce-containing systems, the Ce^3+^/Ce^4+^ ratio is particularly important because it is closely linked to oxygen-vacancy dynamics, surface redox behavior, and oxygen storage/release capability. In La- and Y-modified ZnO systems, the analysis of surface oxidation states and the deconvolution of O 1s components become especially relevant. Nevertheless, XPS also involves serious interpretive risks. Assignments within the O 1s region, especially distinctions among lattice oxygen, oxygen-deficient components, surface hydroxyl groups, adsorbed oxygen, or vacancy-related species, are not always unambiguous. For this reason, O 1s interpretation should be supported by Raman, FT-IR, microstructural observations, and, where available, functional data. The major strength of XPS lies in its ability to reveal the surface chemical state and bonding environment of the dopant; in this respect, it provides a necessary complement to EDX.

Raman and FT-IR spectroscopy are often treated as secondary characterization techniques, yet they can be decisive for distinguishing the actual dopant mechanism. Shifts, broadening, intensity changes, or suppression of ZnO phonon modes can provide indirect evidence of defect density, local strain, crystallite disorder, and bonding perturbation. In h-BN-containing ZnO structures, characteristic B–N-related vibrational features and the E_2_g mode are important indicators that the boron nitride phase or interface has actually been established. In directly boron-doped systems, FT-IR can support the identification of B–O-related bonding environments, particularly when residual organic species have been removed and the spectral region is interpreted cautiously. In systems containing secondary phases such as CeO_2_, La_2_O_3_, or Y_2_O_3_, Raman spectroscopy may, in some cases, be more sensitive than XRD for detecting minor oxide contributions, local disorder, or nanoscale phase formation. Thus, Raman and FT-IR play a strategic role in identifying species that may be structurally relevant but weakly visible in diffraction data.

This multi-technique interpretation leads to an important methodological conclusion: ZnO-based dopant studies should be classified not only according to their performance metrics, but also according to the quality and coherence of the evidence they provide. For example, if a boron-doped ZnO study reports only EDX and UV–Vis data, it is not possible to make a strong claim regarding lattice incorporation or chemical bonding. Similarly, concluding “successful doping” in a Ce-modified ZnO system solely on the basis of XRD peak shifts would be excessive without supporting evidence from XPS, Raman, EDX mapping, or morphology analysis. A robust conclusion can only be reached when phase analysis, microstructural observations, elemental distribution, chemical-state information, and functional performance mutually support one another. The reliability of the comparisons developed in this article rests precisely on this integrated evidence architecture.

The interpretive strengths and limitations of XRD, FE-SEM, EDX, XPS, Raman, and FT-IR data are summarized in Table 5.

## 8. Comparative Analysis of Structural and Morphological Performance

### 8.1. Phase Purity and Crystal Structure Preservation

When structural and morphological performance are evaluated together, a one-dimensional superiority ranking between boron-based and rare-earth-based dopant strategies cannot be established. Nevertheless, several clear comparative tendencies can be identified. The first major distinction concerns phase purity and crystal-structure preservation. In directly B-doped ZnO systems, retention of the dominant wurtzite ZnO phase is generally more feasible, particularly at low and intermediate dopant concentrations. Minor peak shifts, peak broadening, or changes in crystallite size may occur, but the literature generally indicates a more limited degree of phase fragmentation and a lower tendency toward distinct secondary oxide formation compared with rare-earth-modified systems at higher dopant levels. In contrast, rare-earth-based ZnO systems more frequently show the emergence of CeO_2_, La_2_O_3_, Y-containing oxide domains, or surface-segregated rare-earth-rich regions. This behavior should not automatically be interpreted as detrimental, since secondary phases can be functionally beneficial in heterojunction-based designs. However, when the target is the preservation of a predominantly single-phase ZnO structure, boron-based strategies appear, on average, to provide a more structurally conservative and phase-stable modification route [33,34,35,60].

### 8.2. Secondary Phase Formation and Its Functional Significance

A more nuanced interpretation is required for secondary-phase formation. B_4_C/ZnO and h-BN/ZnO systems are intrinsically multiphase architectures; therefore, their success is not determined by the complete elimination of the secondary phase, but by the controlled formation of functional interfaces between ZnO and the boron-containing component. Similarly, in ZnO/CeO_2_ and ZnO/La_2_O_3_ systems, the secondary phase may constitute the primary source of enhanced photocatalytic, sensing, redox, or interfacial charge-transfer behavior. The critical difference lies in the degree of intentionality and controllability. In boron-based hybrids, the secondary phase is generally introduced as a deliberate design element and is interpreted through an interface-engineering framework. In many rare-earth-modified ZnO systems, however, secondary-phase formation may also emerge as a consequence of limited lattice solubility, surface segregation, or incomplete incorporation, and is therefore more difficult to control. This distinction suggests that boron-based hybrids often exhibit more predictable phase behavior, whereas rare-earth-based systems may display more variable phase evolution depending on synthesis route, dopant concentration, and thermal history [37,38,39,42].

### 8.3. Agglomeration Behavior

Agglomeration behavior represents one of the most distinctive criteria for comparing boron- and rare-earth-based ZnO strategies. In h-BN-supported ZnO structures, the two-dimensional surface of h-BN can guide heterogeneous nucleation, restrict uncontrolled particle growth, and reduce clustering, which appears as a consistent advantage in the literature. Similarly, directly B-doped ZnO systems frequently exhibit suppressed grain growth and narrower size distributions when the dopant concentration remains within an appropriate range. By contrast, rare-earth oxide modifiers, particularly CeO_2_ and La_2_O_3_, show a stronger tendency toward surface clustering, local enrichment, and heterogeneous island formation at elevated loadings. Although Y-based dopants may provide relatively more controlled morphological outcomes in selected thin-film and nanostructured systems, boron-based strategies can generally be considered to exhibit a lower agglomeration tendency. This difference is highly relevant because agglomeration directly affects surface homogeneity, active-site accessibility, charge-transport continuity, and the reproducibility of functional performance [40,52,64,65].

### 8.4. Grain Size and Distribution

A similar comparative pattern can be observed in terms of grain-size control. In B-doped ZnO studies, smaller and more uniform crystallites are frequently reported, particularly when the dopant level is optimized and synthesis parameters are carefully controlled. In rare-earth-doped ZnO systems, grain refinement and grain-boundary heterogenization may occur simultaneously. In other words, even when the average crystallite or grain size decreases, the overall distribution may broaden, and rare-earth-rich oxide clusters may form on the surface or at grain boundaries. This can disrupt effective morphological homogeneity despite apparent grain refinement. This behavior is especially evident in La-modified systems, where large ionic size and limited solubility can promote boundary segregation and surface heterogeneity. In Ce-modified systems, oxygen-vacancy enrichment and redox activity are often accompanied by a more complex microstructural arrangement. Although Y-based strategies can produce relatively controlled texture modification in certain thin-film systems, their structural response is still more sensitive to processing variables than that of many directly B-doped ZnO films. Consequently, grain size should not be evaluated as an isolated parameter; it must be interpreted together with distribution width, surface continuity, clustering tendency, and phase uniformity.

### 8.5. Surface Homogeneity

Surface homogeneity is often more decisive than phase purity for practical application transfer. In gas sensors, photocatalytic coatings, transparent conductive films, and thin-film devices, local surface heterogeneity directly affects active-site distribution, carrier transport, adsorption behavior, and device-to-device reproducibility. Boron-based systems, particularly in film-type and supported hybrid architectures, show strong potential for producing smoother surface coverage, more uniform particle arrangements, and more stable morphology. Rare-earth-modified systems, despite their specific functional advantages, may generate irregular island-like morphologies, local oxide-rich domains, or compositionally enriched surface regions. Such heterogeneity can be beneficial in catalytic or sensing contexts when controlled, but it may become problematic when uniform coatings or standardized device elements are required. Therefore, for applications requiring continuous films, homogeneous coatings, or reproducible sensor surfaces, boron-based strategies may provide a more secure and process-tolerant engineering starting point [45,48,50].

### 8.6. Pore Architecture and Surface Accessibility

The existing literature on pore architecture and surface accessibility should be interpreted with particular caution. XRD and FE-SEM provide only indirect or limited information on pore structure; reliable porosity assessment ideally requires complementary techniques such as gas adsorption analysis, pore-size distribution measurements, or surface-area determination. Nevertheless, published microstructural images suggest that h-BN-supported ZnO architectures and certain B-doped structures can generate more open, accessible, and less agglomeration-blocked surfaces. In contrast, CeO_2_- and La_2_O_3_-containing heterostructures may produce surfaces that are chemically more active but also more irregular, locally dense, or partially blocked by oxide-rich clusters. Therefore, in the present study, porosity-related discussion is limited to observable morphological trends rather than being presented as a definitive quantitative conclusion. This methodological caution is essential for avoiding overinterpretation and for defining the requirements of future directly comparable experimental studies.

### 8.7. Processing Window and Reproducibility

From the viewpoint of processing window and reproducibility, the most critical distinction is that boron-based strategies generally exhibit a more moderate and gradual structural evolution, whereas rare-earth-based strategies require more sensitive process control in exchange for potentially stronger functional gains. In Ce- and La-modified ZnO systems, even small variations in dopant concentration, pH, precursor hydrolysis behavior, annealing temperature, atmosphere, or calcination duration can lead to different degrees of surface segregation, secondary-phase formation, and morphological heterogeneity. B-doped films and boron-based hybrids are also affected by pH, precursor chemistry, annealing conditions, and solvent environment; however, their structural response is often more gradual and less prone to abrupt phase complexity. Therefore, when transition from laboratory-scale synthesis to pilot-scale or industrial-scale production is considered, boron-based routes may be regarded as lower-risk options from a process-engineering perspective. Rare-earth-based routes, by contrast, may require tighter control protocols, narrower synthesis tolerances, and more detailed quality assurance to ensure reproducible material behavior.

### 8.8. Integrated Evaluation

The integrated conclusion of this section is that the preferred dopant family depends strongly on how “performance” is defined. If performance is evaluated only by the highest photocatalytic conversion, strongest gas-sensing response, or most intense functional output under optimized laboratory conditions, rare-earth dopants may sometimes appear more attractive. Their redox activity, oxygen-vacancy modulation capability, and heterojunction-forming tendency can generate strong functional responses in selected systems. However, when structural integrity, phase controllability, agglomeration resistance, surface homogeneity, processing tolerance, and reproducibility are evaluated together, boron-based strategies provide a more balanced and more manageable material profile. Thus, boron-based dopants and boron-containing hybrid architectures reveal a strong domain of advantage when performance is understood not merely as a peak output value, but as a combined measure of functional response, structural stability, and scalable process reliability.

This interpretation should not be read as a binary replacement argument in which rare-earth-containing systems function merely as a negative comparator for boron-based strategies. Rare-earth additives remain technically rational, and in some application windows indispensable, when the target function depends on Ce^3+^/Ce^4+^ redox cycling, oxygen-vacancy buffering, La-related surface basicity and grain-boundary modification, Y-assisted energy-level adjustment, luminescence tuning, or deliberately constructed rare-earth-oxide heterointerfaces. The relevant decision is therefore conditional: boron-based systems provide a broader and more scalable default platform when morphology, phase retention, processing tolerance, and supply security dominate the design space, whereas rare-earth-based systems remain justified when their functional role is experimentally verified, reproducible, and not readily substitutable by boron-based or lower-risk alternatives.

**Figure 2:**
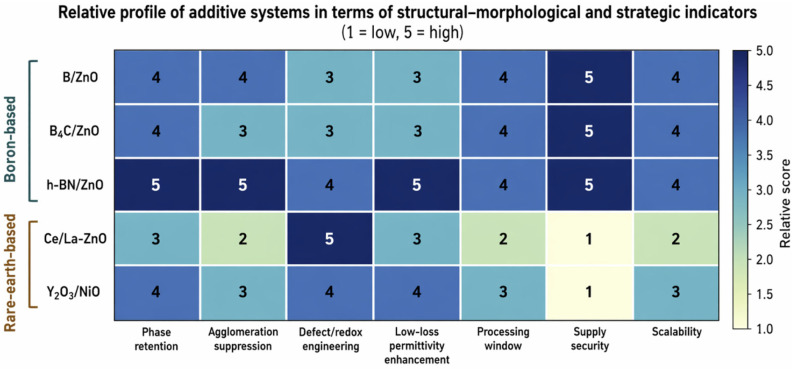
Relative qualitative profile of selected boron-based and rare-earth-based dopant systems in terms of structural, morphological, and strategic indicators. The figure summarizes directional trends from the cited studies and is not a statistically normalized ranking.

The joint interpretation of structural, morphological, and strategic indicators reveals the relative profiles of the dopant families. This comparative synthesis is presented in Figure 2 [5,10,11,12,13,14,15,16,17,18,19,20,21,22,33,34,35,36,37,38,39,40,41,42,43,44,45].

The structural and morphological comparison matrix of boron-based and rare-earth-based strategies is summarized in Table 6.

## 9. Implications for Application Areas

The structural and morphological effects of dopant strategies generate functional value in different ways depending on the target application. Gas sensing, photocatalysis, transparent conductivity, photoluminescence, and energy conversion do not share the same structural requirements or performance priorities. For instance, low optical scattering, high transmittance, and a smooth homogeneous film surface are critical for transparent conducting films, whereas controlled heterojunctions, defect-rich surfaces, and active adsorption sites may be more beneficial for photocatalytic and sensing applications. Therefore, dopant selection should not be framed through an application-independent discourse of superiority. Instead, it should be determined by the structural, chemical, and interfacial priorities required by the target function [1,26,27].

Transparent conducting and optoelectronic applications constitute one of the areas in which B-doped ZnO exhibits a particularly strong profile. Studies on B-doped ZnO thin films show that electrical conductivity can be improved while maintaining high optical transmittance in the visible region, and that film morphology can be controlled in a manner compatible with scalable processing. The ability of boron to generate meaningful performance improvements at relatively low dopant levels through aerosol-assisted chemical vapor deposition, sputtering, and sol–gel-based methods also makes this strategy relevant as a potential alternative or complementary route to indium-containing transparent conducting systems. Rare-earth-modified ZnO, by contrast, is more prominent in optoelectronic applications involving luminescence tuning, UV emission, or defect-mediated optical functionality. However, it does not offer as systematic an advantage as boron in terms of transparent conductivity, low-scattering film architecture, and morphology-controlled coating formation [36,45,48,49,50].

For gas-sensing applications, the comparison is more complex. The gas-sensing behavior of ZnO is closely governed by chemisorbed oxygen species, oxygen-vacancy concentration, grain size, electron depletion layers, surface adsorption capacity, and intergranular potential barriers. In this context, rare-earth dopants such as Ce and Y can provide highly effective results because they can enhance surface reaction kinetics, oxygen-vacancy dynamics, and adsorption activity. Significant improvements have been reported in sensors targeting H_2_S, NH_3_, and various volatile organic compounds. Nevertheless, these performance gains must be balanced against possible morphological heterogeneity, phase segregation, and device-to-device reproducibility issues. B-doped ZnO thin films have also shown promising sensing behavior, particularly for humidity and selected gas species, while offering a more consistent platform owing to their comparatively homogeneous film morphology. Thus, in gas-sensing applications, rare-earth dopants may represent a route toward maximum response intensity, whereas boron-based strategies may provide a more stable and reproducible sensor platform [26,66,69].

Photocatalysis represents one of the most important intersection areas between the two dopant families. On the boron-based side, direct B doping, B_4_C/ZnO composites, and h-BN/ZnO interfaces can all contribute to photocatalytic enhancement through different mechanisms. On the rare-earth side, Ce/CeO_2_- and La/La_2_O_3_-based ZnO systems are particularly effective because of their redox activity, oxygen-vacancy modulation, and heterojunction-forming capability. The key distinction lies in the dominant enhancement mechanism. Redox-active phases such as CeO_2_ can promote reactive oxygen species generation and facilitate charge separation through oxygen storage/release behavior. In contrast, h-BN- or B_4_C-containing ZnO systems tend to operate through improved dispersion, interfacial charge transfer, adsorption–photocatalysis synergy, and suppression of particle agglomeration. Therefore, photocatalytic “success” cannot be reduced to a single conversion value. In certain dye-degradation experiments, rare-earth-modified ZnO may achieve higher degradation efficiency, whereas boron-based hybrids may become more competitive when long-term stability, particle dispersibility, interfacial controllability, and synthesis reproducibility are considered together [37,42,53,57,60,68].

In photoluminescence and luminescence-oriented applications, the specific advantage of rare-earth dopants becomes more pronounced. Ce- and Y-modified ZnO systems can provide considerable potential for tuning visible emission, controlling defect-related luminescence, and enabling nanophosphor behavior relevant to solid-state lighting and optoelectronic devices. These functions depend on complex electronic states, localized energy levels, and defect-mediated emission processes that cannot be explained solely by surface homogeneity or grain-size control. Therefore, in this application area, direct replacement of rare-earth dopants is technically more difficult. Boron doping may influence optical behavior indirectly by modifying carrier concentration, film quality, crystallinity, or defect density, but it does not directly reproduce the specific emission centers and electronic transitions associated with rare-earth elements. This distinction demonstrates why policy recommendations should not be framed as the complete exclusion of rare-earth elements, but rather as their selective use where functional indispensability is clearly demonstrated [31,55,56].

In energy conversion and photoelectrochemical applications, a similar pattern of conditional superiority can be observed. B-doped ZnO films and boron-based interfacial architectures are attractive because of their transparent conductivity, improved film continuity, relatively low-cost processing, and compatibility with scalable deposition methods. Rare-earth-based structures, such as Y-doped ZnO nanorod arrays or Ce-modified heterostructures, may provide specific advantages through increased carrier mobility, enhanced charge density, improved defect-mediated transport, or stronger photoresponse. Therefore, dopant selection for energy devices should not be based solely on the highest laboratory-scale efficiency value. Device architecture, film continuity, precursor chemistry, interfacial stability, processing reproducibility, and supply-chain exposure must also be considered. The use of a dopant dependent on a vulnerable or long supply chain is not always rational for a marginal efficiency improvement; however, if that dopant is required to exceed a critical functional threshold, rare-earth modification may be technically justified [48,67].

From an application-oriented perspective, the overall conclusion is that boron-based strategies offer broader engineering applicability, stronger process tolerance, and a more balanced combination of structural stability, surface homogeneity, and microstructural controllability. Rare-earth-based strategies, in contrast, provide more specialized but sometimes indispensable functional gains, particularly where redox activity, luminescence control, oxygen-vacancy modulation, or high surface reactivity is required. Therefore, publicly supported research programs should not evaluate dopants only through laboratory performance metrics. Instead, application areas should be differentiated according to whether they require scalable structural reliability or high-intensity functional intervention, and dopant selection should be guided by both technical benefit and strategic material cost.

## 10. Critical Raw Material Policies and Supply Security

The critical raw material debate increasingly establishes a direct analytical bridge between materials science and political economy. Whether a raw material is classified as “critical” depends not only on geological scarcity, but also on economic importance, supply risk, substitutability, trade-network concentration, refining capacity, and the geographical distribution of processing infrastructure. Recent critical raw material assessments by the European Commission adopt this multidimensional framework when evaluating raw material dependencies associated with the green transition, digital technologies, and advanced manufacturing sectors. The same perspective is necessary for ZnO-based dopant strategies, because the security, scalability, and sustainability of a dopant used at the laboratory scale become integral components of material selection when the technology is considered for industrial transition [5,13,14].

Although Türkiye is used later as a detailed producer-country example because of its boron-resource position, the decision logic developed in this study is not country-exclusive. The same framework can be transferred to any jurisdiction by re-weighting four variables: domestic resource access, dependence on external refining capacity, technological substitutability, and application-specific performance threshold. Two threshold rules are therefore used. A material route must first satisfy a technical threshold, defined by phase stability, reproducible morphology, defensible dopant/phase assignment, and application-relevant function. Only after this threshold is met does the strategic threshold become decisive, including supply concentration, import dependence, price volatility, refining bottlenecks, circularity potential, and domestic value-chain alignment. Thus, the policy dimension should be read as a transferable producer/importer-context model rather than as a Türkiye-only argument.

Rare-earth elements represent a typical high-risk category within this framework. Although elements such as Ce, La, and Y differ in crustal abundance, the decisive factor in the global value chain is not merely ore availability, but the concentration of separation, solvent extraction, purification, oxide production, and refined product manufacturing capacity. The literature has extensively documented the dominant role of China in several stages of the rare-earth value chain, as well as the systemic market fluctuations generated by past export restrictions and supply disruptions. Therefore, for any country dependent on external supply, the use of rare-earth elements is not only a materials-selection issue, but also a matter of geopolitical sensitivity, price volatility, industrial resilience, and technological strategy [4,6,8,9,70]. In the European context, discussions on critical raw material production from primary sources further demonstrate that the presence of reserves alone does not automatically translate into secure supply. Processability, refining infrastructure, permitting capacity, environmental constraints, and industrial know-how are equally decisive factors [71].

Although the recycling of rare-earth elements and their integration into circular economy models constitute an important research direction, the current literature indicates that recycling alone is unlikely to eliminate primary supply risk in the short term. Rare-earth recycling may remain constrained by low concentrations in end-use products, complex product architectures, inefficient collection systems, separation difficulties, and high processing costs. This does not imply that advanced materials containing rare-earth elements should be avoided in scientific research. Rather, it means that supply vulnerability should be treated as a direct design variable in large-scale material platforms, particularly when they are supported by public funding. In other words, dopants that provide high functionality but carry high geopolitical or supply-chain risk should be selected when their functional contribution is clearly necessary and not readily substitutable [7,72,73,74].

On the boron side, a more asymmetric policy picture emerges. Boron may be evaluated within the critical raw material framework for certain importing economies; however, for a country possessing substantial reserves, production capacity, and industrial experience, the same material can become a source of strategic advantage rather than strategic vulnerability. In the context of Türkiye, the significance of boron is not limited to the existence of domestic reserves. It also lies in the opportunity to establish vertical integration across a value chain extending from mining and chemical processing to ceramics, coatings, composites, energy materials, and advanced functional components. Therefore, boron-based dopant strategies cannot be placed in the same risk category as rare-earth dopants that depend on externally controlled supply chains. In this sense, the status of a “critical material” does not generate the same policy implication for every country: producer countries and importing countries evaluate material criticality from fundamentally different positions [10,11,12].

This asymmetry explains why material selection must be interpreted according to national context. The critical raw material strategy developed by the European Union around supply diversification, domestic production, recycling, strategic reserves, and reduced dependency is also instructive for Türkiye. However, Türkiye’s producer position in the boron sector makes boron-based materials more than an ordinary scientific alternative. If two dopant strategies operate within a comparable technical performance window, prioritizing the strategy based on a domestically accessible resource becomes rational within public R&D mechanisms. The justification is not limited to cost reduction. It also includes the accumulation of domestic know-how, development of an industrial ecosystem, strengthening of export potential, reduction in supply-chain exposure, and advancement of technological sovereignty [5,75].

A key normative implication follows from this perspective: material performance alone is not a sufficient criterion for public support. A dopant system may deliver high efficiency or strong functionality under laboratory conditions; however, if it depends on an imported input characterized by volatile pricing, concentrated refined-product supply, limited substitutability, and geopolitical exposure, it should occupy a more cautious position within public funding priorities. Conversely, systems based on domestically available and strategically accessible inputs should be prioritized when the performance gap is limited or when the target application does not require a function uniquely provided by the higher-risk material. This approach does not constrain scientific freedom; rather, it aligns public research investment with longer-term industrial resilience, supply security, and strategic value creation.

In this context, the policy comparison between boron-based and rare-earth-based dopants should be made according to the application window in which technical superiority becomes strategically meaningful. Rare-earth elements combine the possibility of high functional leaps with substantial supply vulnerability, whereas boron represents, in many applications, a combination of sufficient or high technical performance with lower strategic risk and stronger domestic value-chain potential. From a policy perspective, the decisive criterion is whether the function provided by a rare-earth dopant can be clearly demonstrated to be non-substitutable by boron-based or more accessible alternatives. Without this distinction, material-policy decisions risk being reduced to a short-term competition over peak performance metrics rather than being evaluated through the broader framework of functionality, scalability, supply security, and national technological capacity.

The decision matrix that jointly interprets technical performance and critical raw material policy is summarized in Table 7.

## 11. Public R&D Priorities: Türkiye as an Illustrative Producer-Country Case

The Türkiye-focused discussion below is used as an applied case of the broader decision model rather than as a limitation of the study’s relevance. In countries without a domestic boron advantage, the same framework would produce different weights, for example by prioritizing recycling capacity, secure import contracts, local processing infrastructure, or substitute dopant families with lower supply risk.

In the context of Türkiye, the determination of public R&D priorities represents the concrete policy counterpart of the technical and strategic findings developed in this study. The approach proposed here neither idealizes boron-based materials nor excludes rare-earth elements from advanced materials research. Rather, the central principle is to position materials associated with domestic resource advantages as default research platforms, while reserving imported and strategically vulnerable dopants for targeted applications in which clear functional necessity can be demonstrated. Such an approach does not restrict scientific diversity. Instead, it aligns the direction of public research investment more closely with national materials sovereignty, industrial deepening, and long-term technological resilience.

Within this framework, the first policy recommendation is the development of boron-prioritized basic platform programs. Research projects focusing on B doping, B_4_C-based composites, and h-BN-supported interfaces in ZnO, SnO_2_, Fe_2_O_3_, and related oxide systems should receive broad-based support in fields such as sensors, photocatalysis, transparent conducting films, energy interfaces, and functional coatings. The purpose of such programs should not be limited to the production of a single device or material output. More importantly, they should generate synthesis knowledge, characterization competence, prototype-development capability, and scale-up know-how, thereby enabling domestic boron resources to be transformed into high-value-added advanced materials. In this sense, boron-centered R&D programs could establish a direct knowledge bridge between mining, chemical processing, materials engineering, and industrial technology development [10,11,12].

The second policy recommendation is the adoption of a selective and well-justified support model for rare-earth-based dopants. Ce-, La-, or Y-modified ZnO systems should be supported when they provide clearly indispensable functionality in areas such as luminescence, highly selective gas sensing, advanced heterojunction photocatalysis, or specialized photoelectrochemical applications. However, such support should not be granted automatically merely because a higher laboratory-scale efficiency or stronger functional response has been reported. Project proposals involving rare-earth dopants should explicitly justify why the proposed function cannot be achieved using boron-based or other more accessible alternatives. They should also explain how supply-security risks will be managed and, where possible, whether recycling, secondary sourcing, substitution, or reduced-loading strategies can be incorporated. In this way, materials policy can accommodate both technical ambition and strategic rationality.

The third recommendation is to encourage the comparative logic of “same matrix, same process, different dopant” in publicly funded research calls. A major limitation of the existing literature is that different dopants are often tested under different synthesis routes, precursor systems, thermal histories, and characterization protocols. This makes it difficult to derive reliable scientific and policy conclusions from isolated performance comparisons. In publicly supported comparative projects, the direct evaluation of B, B_4_C, h-BN, CeO_2_, La_2_O_3_, and Y_2_O_3_ systems under the same ZnO precursor chemistry, synthesis route, dopant-loading range, and thermal-treatment regime could be required. Such a design would improve the quality of academic knowledge while allowing a more objective determination of which advantages are genuinely dopant-derived and which arise from processing differences. This approach would also help transform a fragmented literature base into a national-scale comparative knowledge infrastructure.

The fourth recommendation concerns centralized programs that connect characterization infrastructure with materials policy. In boron-doped and boron-containing systems, XPS, Raman spectroscopy, FT-IR, and advanced surface analyses are essential for the reliable detection and interpretation of low-atomic-number elements. In rare-earth-modified systems, multi-technique characterization is necessary to distinguish true lattice incorporation from surface segregation, oxide clustering, grain-boundary enrichment, or heterojunction formation. Therefore, public support should not be directed only toward synthesis-oriented projects. It should also support comparative characterization standards, shared analytical protocols, and national reference datasets. Such an approach would strengthen the advanced materials literature both quantitatively and qualitatively, while reducing the gap between laboratory-scale scientific output and industrially relevant material validation.

The fifth and longer-term recommendation is the establishment of a value-chain-oriented clustering policy for boron-based advanced materials. If boron-modified oxides developed in university laboratories are supported through public procurement, pilot production lines, standardization programs, prototype validation, and technology-readiness-level advancement mechanisms, it becomes possible to create a materials ecosystem that extends beyond publication generation. In this ecosystem, rare-earth-based research would not be excluded, but would be positioned more selectively within a high-risk/high-reward category. Such differentiation would align with Türkiye’s existing boron-resource advantages while also creating a more resilient research portfolio against the uncertainties of global critical raw material politics.

As a result, the proposed model for public R&D prioritization is three-layered. First, boron-based strategies should be prioritized in broad platform areas where they provide sufficient or strong technical performance together with domestic value-chain potential. Second, rare-earth elements should be used selectively where specific functionality is technically indispensable and cannot be readily substituted. Third, comparative data standards should be required for both dopant families in order to ensure that public funding decisions are based on robust evidence rather than isolated performance claims. This threefold approach appears to be the most rational route for increasing strategic autonomy without compromising scientific quality.

Another important issue in public prioritization is that evaluation criteria should not be limited to the number of publications, patent applications, or short-term performance outputs. For boron-based advanced oxide projects, additional indicators should be defined, including the use of domestic precursors, process reproducibility, characterization depth, industrial partnership, technology-readiness-level progression, prototype validation, and substitution potential. For rare-earth-based projects, these criteria should be complemented by a supply-risk mitigation plan, a recovery or secondary-source scenario, and a functional necessity analysis that justifies dependence on imported or strategically vulnerable inputs. In this way, funding mechanisms can reward research teams that not only generate scientific novelty, but also pursue strategically informed material design.

Moreover, national material roadmaps should be updated periodically to strengthen the interaction among universities, public institutions, and industry. Priority areas for boron-based sensors, photocatalytic coatings, transparent conducting films, boron nitride-based hybrid interfaces, and boron-containing energy materials could be reassessed at three-year intervals. During the same period, rare-earth-based research could be concentrated in selected applications where high strategic returns are expected despite the risk of import dependence. Such roadmaps should not be understood as a reduction in scientific research to immediate market demand. On the contrary, they would enable long-term public investment to evolve into a learning-oriented, data-generating, and strategically coordinated research portfolio rather than remaining fragmented across unrelated projects.

### Proposed Selection Principles for Public Funding Decisions

To enable more objective public funding decisions, dopant strategies can be evaluated along four main axes: technical necessity, structural stability profile, supply security, and contribution to the domestic value chain. The technical necessity axis examines whether the dopant provides a genuinely non-substitutable function. The structural stability axis evaluates the reliability of the processing window in terms of phase purity, agglomeration tendency, surface homogeneity, reproducibility, and morphology control. The supply-security axis measures sensitivity to import dependence, refined-product concentration, price volatility, and geopolitical disruption. The domestic value-chain axis assesses how readily the dopant strategy can be integrated with domestic precursors, local processing capacity, industrial partnerships, and scale-up infrastructure. When these four criteria are considered together, it becomes clearer why boron-based systems should be prioritized in broad platform programs, whereas rare-earth-based systems should be directed toward more selective and function-specific application areas.

This decision framework can be directly integrated into project evaluation processes. For example, if a project proposes a CeO_2_-modified ZnO photocatalyst, the assessment should not focus only on laboratory degradation efficiency or short-term conversion performance. It should also ask why a comparable function cannot be achieved using h-BN/ZnO, B-doped ZnO, B_4_C/ZnO, or another more accessible material route. It should further examine how Ce-related supply risk will be managed and whether the material introduces scalability challenges due to phase segregation, synthesis sensitivity, or dependence on imported refined inputs. Similarly, a project proposing a B-doped ZnO film should not treat domestic resource advantage as an automatic justification. It must demonstrate whether boron genuinely provides structural, optical, electrical, or processing advantages, and whether the characterization evidence is sufficient to support the proposed mechanism. In this way, the decision model becomes not a boron-biased framework, but a balanced evaluation mechanism that prioritizes material systems according to both evidential strength and strategic dependence.

A similar update is required in the academic incentive system. Evaluation based solely on publication quantity may direct research groups toward short-term, easily publishable topics with limited strategic value. In boron-based advanced materials research, however, outputs such as process standardization, comparative characterization depth, pilot-scale trials, reproducible synthesis protocols, and open materials datasets may generate fewer publications but higher national value. Therefore, evaluation metrics should also include dataset quality, standardized comparative design, technology-readiness progression, interlaboratory reproducibility, open database production, and indicators of industrial collaboration. Such a framework would constitute the institutional counterpart of the materials-governance approach proposed in this study.

Ultimately, public funding decisions should be regarded not merely as instruments for supporting scientific novelty, but also as tools for directed strategic capacity building. If the objective is not only to contribute additional publications to the global literature, but also to establish a more independent, resilient, and value-generating production infrastructure in critical technologies, then material selection must also be aligned with this goal. Boron-based dopant strategies therefore deserve high priority across a broad range of platform applications, whereas rare-earth-based strategies should proceed under a strong but selective support regime in niche areas where their functional contribution is demonstrably indispensable.

## 12. Reasons for Contradictions in the Literature and the Scalability Debate

### 12.1. Sources of Contradictory Results in the Literature

The apparently contradictory findings reported in the literature on boron- and rare-earth-modified ZnO systems do not necessarily indicate that the field is scientifically immature. Rather, they reveal that the experimental context has not yet been sufficiently standardized. The same dopant may reduce crystallite size in one study while increasing it in another, or it may improve surface homogeneity under one synthesis condition while promoting agglomeration under another. Such discrepancies often arise not from the intrinsic “nature” of the dopant itself, but from differences in precursor purity, solvent chemistry, solution pH, hydrolysis kinetics, hydroxylation degree, annealing atmosphere, heating rate, substrate effects, and post-treatment conditions. In a defect-sensitive oxide such as ZnO, even relatively small differences in the initial structural or chemical state can be sufficient to redirect the effect of the dopant [1,2].

In boron-doped systems, one of the most important sources of contradictory interpretation is the difficulty of directly identifying the actual chemical incorporation mode of boron. At low dopant contents, boron may be partially incorporated into the ZnO lattice, form surface B–O-related complexes, interact with oxygen-deficient regions, or become enriched near grain boundaries. Each of these possibilities can produce a different structural, optical, or electrical outcome. Therefore, if a study draws mechanistic conclusions only from electrical, optical, or general morphological data, it is not surprising that different research groups may report different trends for nominally similar B loadings. For this reason, the most convincing studies in the boron-based ZnO literature are generally those that combine controlled synthesis conditions with surface-sensitive and bonding-sensitive characterization methods [33,48,51].

In rare-earth-modified systems, the main source of contradiction is the frequent conflation of lattice doping with secondary-phase formation or surface segregation. Elements such as Ce, La, and Y may show different degrees of compatibility with the ZnO lattice; however, beyond low concentration ranges, rare-earth enrichment at surfaces, grain boundaries, or oxide-rich domains can frequently occur. In some cases, this behavior improves functionality by producing beneficial heterojunctions, redox-active surface domains, or enhanced adsorption sites. In other cases, it deteriorates performance by disrupting homogeneity, increasing carrier scattering, or promoting agglomeration. While part of the literature explicitly discusses this dual behavior, some studies present dopant incorporation automatically as “successful doping.” Yet similar XRD patterns may correspond to fundamentally different nanoscale distributions and incorporation modes. Therefore, contradictions in the rare-earth-based ZnO literature are often directly related to the depth and coherence of characterization [38,44,64].

Another important source of contradiction is the diversity of application metrics used to define “success.” For example, a high defect density may initially appear beneficial for photocatalysis by increasing adsorption and reactive-site density; however, the same defect density may become disadvantageous in optoelectronic thin films by increasing carrier trapping or scattering. Similarly, a heterogeneous surface may enhance gas-sensing response by increasing reactive sites, whereas the same heterogeneity may impair the stability and reproducibility of a thin-film transistor. Therefore, a dopant strategy cannot be classified as universally “good” or “bad” independently of application context. Many studies appear contradictory because they are, in fact, comparing different definitions of performance. The present study attempts to reduce this ambiguity by foregrounding structural, morphological, chemical, and process-related criteria before discussing application-specific outcomes.

Consequently, divergent findings in the literature point less to inconsistency within the field than to the absence of a coherent comparative framework. As systematic studies conducted under the same host matrix, same synthesis route, same dopant-loading series, same thermal history, and same characterization protocol become more common, many results that currently appear contradictory will become mechanistically understandable. Therefore, the experimental roadmap proposed in this study is necessary not only for generating new data, but also for reducing the epistemic fragmentation that currently limits the comparability of ZnO dopant studies.

### 12.2. Scalability, Process Economics, and Environmental Dimension

The relationship between material selection and public policy is not limited to raw material supply security; scalability, process economics, and environmental manageability are equally important. A material architecture that performs well at laboratory scale may remain industrially limited if it cannot be reproduced at larger scales, depends on an excessively narrow processing window, or requires highly sensitive synthesis control. From this perspective, boron-based ZnO strategies generally present a more scalable profile, particularly in thin-film and powder-based syntheses. The ability of boron doping to generate functional effects at relatively low dosages in sol–gel, sputtering, aerosol-assisted CVD, and related deposition routes keeps process complexity comparatively manageable. Rare-earth dopants, by contrast, often require stricter stoichiometric control, more careful dispersion management, and tighter thermal-processing conditions, which may create difficulties in maintaining phase and morphological homogeneity during scale-up [45,48,49].

From a technoeconomic perspective, the unit price of the dopant alone is not a sufficient indicator of process feasibility. The required purity level of the dopant, refining pathway, precursor availability, supply reliability, additional processing steps, quality-assurance requirements, yield losses, and waste-management costs also shape the overall economic profile. In rare-earth oxide dopants, the effective cost of implementation often includes not only the market price of Ce-, La-, or Y-based inputs, but also dependence on refined product quality, stable supply agreements, and exposure to geopolitical volatility. In boron-based systems, by contrast, logistics, supply uncertainty, and strategic dependence may be lower in contexts where domestic resource and processing advantages exist. This distinction becomes particularly important in publicly supported scale-up programs, since the objective is not merely to demonstrate scientific feasibility, but also to establish a sustainable and reproducible production pathway.

The environmental process dimension also requires careful comparison. Rare-earth mining, separation, and purification are frequently associated with high chemical-processing burdens, complex waste streams, and significant environmental externalities. Although recovery, recycling, and circular economy approaches for rare-earth elements are developing, these routes still face technical, economic, and logistical barriers. Boron-based systems are not exempt from environmental impacts, particularly when mining, chemical conversion, and high-temperature processing are considered. However, in the context of a producer country, a shorter and more domestically governed supply chain may make environmental oversight, regulatory control, and process optimization more visible and manageable. Therefore, environmental sustainability should be incorporated into the selection of advanced oxide dopant strategies in the same way as supply security and technical performance [72,74,75].

Taken together, these scalability- and process-related considerations reinforce the central argument of this study: laboratory performance alone does not necessarily imply strategic rationality. When scalability, supply security, process manageability, and environmental governability are evaluated together, boron-based strategies appear more defensible in many broad platform applications. Rare-earth-based dopants, by contrast, should be treated as high-value and high-specificity solutions for functions that clearly justify their higher processing sensitivity and supply-chain vulnerability. This distinction provides a more realistic framework for material prioritization from both scientific and industrial-policy perspectives.

### 12.3. Reporting Standard, Reproducibility, and Comparability

One of the fundamental problems in the existing literature is that structural and morphological findings are reported at highly uneven levels of detail. In some studies, only XRD and SEM data are presented, whereas others include XPS, Raman spectroscopy, FT-IR, and detailed elemental mapping. Even when advanced characterization is provided, critical synthesis parameters such as precursor purity, solution pH, hydrolysis conditions, aging time, calcination atmosphere, heating rate, substrate type, and dopant-introduction route may not be reported with sufficient clarity. Yet, for reliable comparison among dopant strategies, both the synthesis history and the characterization evidence chain must be reported together. The ZnO sensor and surface-defect literature clearly demonstrates that performance frequently originates not only from dopant chemistry, but also from processing history, surface state, and defect evolution [15,26,27].

For future directly comparative experimental studies, a minimum reporting framework should include absolute dopant loading, precursor purity, synthesis pH, solvent system, aging duration, calcination or annealing window, heating and cooling rates, atmosphere, substrate type where relevant, and the exact dopant-introduction route. On the characterization side, it should include the crystallite-size calculation method, XRD peak-fitting approach, quantitative SEM image analysis, representative particle/grain-size distributions, EDX mapping conditions, XPS calibration procedure, and XPS deconvolution protocol. For boron-containing systems, because of the low atomic number and weak analytical contrast of boron, strong claims regarding incorporation mode should not be made without support from complementary techniques such as XPS, Raman spectroscopy, and FT-IR. For rare-earth-modified systems, true lattice incorporation and surface segregation should likewise not be treated as equivalent without an integrated interpretation based on XRD, XPS, elemental mapping, and morphology analysis. Such reporting discipline would reduce overinterpretation in both boron- and rare-earth-based systems while substantially improving interlaboratory comparability [25,33,48,65].

## 13. Research Gaps and Direct Experimental Roadmap

The strongest methodological conclusion emerging from the existing literature is that a sufficiently direct and controlled head-to-head comparison between boron-based and rare-earth-based ZnO strategies has not yet been systematically established. Most studies on boron- and rare-earth-modified ZnO systems have been conducted by different research groups using different precursor chemistries, solvent systems, pH conditions, annealing programs, dopant-loading ranges, characterization depths, and final application targets. This heterogeneity limits the possibility of drawing definitive comparative conclusions, even when clear trends can be identified across the literature. Therefore, the natural continuation of the present study is a direct experimental comparison designed under the same host matrix and the same processing conditions. Such a design would not only address a scientific gap, but also provide a methodological standard that could be adopted in future public funding calls and national advanced materials programs.

In the proposed experimental framework, ZnO should again be selected as the common matrix because it provides one of the most suitable platforms in terms of active defect chemistry, processability in both powder and thin-film forms, and comparability with the existing literature. The study could be organized into two complementary branches. In the first branch, a direct lattice-doping approach would be adopted, in which B, Ce, La, and Y atomic dopants are introduced into ZnO at predefined molar percentages. In the second branch, a composite or interface-based approach would be used, in which B_4_C, h-BN, CeO_2_, La_2_O_3_, and Y_2_O_3_ additives are incorporated into the same ZnO matrix at predefined weight percentages. In this way, lattice doping and secondary-phase/interface strategies could be evaluated within the same broader experimental program, but under separate hypothesis sets. This distinction is essential for eliminating the conceptual ambiguity frequently encountered in the literature, where lattice incorporation, composite formation, and heterointerface engineering are often discussed as if they were equivalent forms of doping.

From the perspective of experimental control, the most critical requirement is to keep the synthesis route as common as possible across all dopant families. For example, a sol–gel route followed by controlled annealing, or a hydrothermal route followed by an equivalent thermal-treatment regime, could be selected. Regardless of the preferred route, solution concentration, precursor ratio, solvent system, pH, aging time, drying conditions, calcination temperature, heating rate, annealing duration, and atmosphere should be kept constant among the dopant systems. Only under such conditions can the observed structural and morphological differences be attributed primarily to dopant chemistry rather than to processing history. Considering that pH is particularly influential in boron-based ZnO systems, while temperature, oxide formation, and segregation tendency are especially critical in rare-earth-based systems, process standardization would constitute the methodological backbone of a reliable comparative study [34,44,47].

Dopant levels should also be selected according to a stepwise and comparable logic. For direct atomic doping, a series such as 0.5, 1, 3, and 5 mol% may be appropriate, whereas for composite or interface-based additives, a series such as 1, 3, and 5 wt.% could be used. The aim should not be to randomly identify the “best dopant ratio,” but to monitor how the structural, morphological, and chemical responses evolve across low, intermediate, and relatively high loading regions. Such a compositional design would make it possible to determine, within the same experimental framework, the concentration at which boron doping begins to introduce excessive trap states or structural disorder, as well as the loading level at which Ce, La, and Y systems begin to show stronger surface segregation, secondary-phase formation, or morphological heterogeneity. This approach would also clarify the discussion of optimum dopant ranges, which remains fragmented across the current literature.

The characterization set should be built on the core techniques identified throughout this study: XRD, FE-SEM, EDX, XPS, Raman spectroscopy, and FT-IR spectroscopy. For XRD, the analysis should not be limited to phase assignment. Where possible, Rietveld refinement or quantitative phase analysis should be included, together with crystallite-size trends, lattice-parameter evaluation, peak broadening analysis, and phase-fraction estimation. FE-SEM images should not remain at the level of qualitative description. Grain size, particle-size distribution, surface coverage, and agglomeration tendency should be quantified through image-analysis metrics. EDX should be supported by elemental mapping and line-scan analysis, particularly for rare-earth dopants, where spatial segregation and oxide-rich regions are central concerns. For boron-containing systems, EDX findings should always be accompanied by an explicit limitation statement because of the low atomic number and weak elemental contrast of boron. In XPS, B 1s, Ce^3+^/Ce^4+^, La^3+^, Y^3+^, Zn 2p, and O 1s deconvolutions should be interpreted according to a standardized scheme, while Raman and FT-IR analyses should be used as complementary evidence for phase distinction, defect-related disorder, B–O/B–N bonding, rare-earth oxide signatures, and interfacial interactions.

The experimental design should also explicitly acknowledge that relying solely on FE-SEM to evaluate pore structure and surface accessibility is limited. Although it may not be part of the minimum core characterization set, gas adsorption analysis should ideally be incorporated as an additional technique to obtain robust information on specific surface area, pore-size distribution, and pore accessibility. If the study is limited to the core techniques, conclusions related to porosity should be formulated cautiously, and more accurate expressions such as “morphological accessibility,” “surface openness,” or “agglomeration-limited accessibility” should be preferred. This type of methodological transparency would strengthen the reliability of the study by clearly defining the evidential limits of each claim.

Under this experimental framework, four main hypotheses could be tested. H1: Direct boron doping will provide greater preservation of the ZnO phase and lower agglomeration tendency than Ce, La, and Y doping at equivalent molar loadings. H2: Ce-based dopants will generate the most pronounced changes in oxygen-vacancy-related and redox-active surface behavior, but will also show the highest risk of secondary-phase formation and surface heterogeneity. H3: h-BN/ZnO interfaces will exhibit the lowest agglomeration tendency and the most favorable surface-homogeneity profile among the composite/interface strategies. H4: When the technical results are evaluated together with the supply-security matrix, public funding priority will shift toward boron-based systems across broad platform applications, whereas rare-earth-based strategies will be justified mainly in functionally indispensable niche areas. Testing these hypotheses within a single controlled study would strengthen the contribution of the work for both materials science and materials policy.

From a publication-strategy perspective, such a research design would also be highly valuable. The first publication could be structured as an experimental materials science article focused on synthesis, structure, morphology, defect chemistry, and property relationships. The second publication could be designed as a materials-policy interface article that interprets the same dataset in terms of critical raw materials, supply security, public R&D prioritization, and national value-chain development. In this way, the present study would not function merely as a conceptual synthesis, but as an academic infrastructure that can be directly transformed into a coherent research program.

The proposed comparative research design that can be directly transformed into an experimental article is summarized in Table 8.

## 14. Transferability of ZnO Findings to SnO_2_, Fe_2_O_3_, and NiO Systems

### 14.1. Limits of Cross-Matrix Transferability and Its Importance as a Separate Field of Investigation

Although ZnO constitutes the main comparison matrix of this study, a question of considerable academic importance for advanced oxide materials is the extent to which the conclusions derived from ZnO can be transferred to other oxide systems. Methodologically, a strong comparative materials study should not merely reproduce morphological trends observed within a single host matrix. It should also examine how ionic-radius mismatch, host crystal symmetry, dominant defect mechanisms, carrier type, surface chemistry, and interfacial reactivity reshape dopant behavior across different oxide families. Extending the boron- and rare-earth-based dopant logic developed for ZnO to technologically important oxides such as SnO_2_, Fe_2_O_3_, and NiO therefore enables the study to generate mechanism-based value rather than remaining a descriptive comparison limited to one matrix [1,2,3,15,23,24].

In conducting such a transferability analysis, two distinct levels must not be conflated. The first level concerns whether a given dopant produces a similar directional effect in different matrices. For example, the tendency of boron-based modification to suppress grain growth, regulate surface morphology, or reduce agglomeration may also be observed in certain n-type oxide systems. The second level concerns the assumption that the same dopant produces an effect of identical magnitude in all matrices, which is generally incorrect. This distinction is particularly important for rare-earth-modified systems, where increasing ionic-radius mismatch, limited solubility, and segregation tendency may confine the beneficial doping regime to an extremely narrow compositional window. Therefore, the aim is not to claim that all observations made for ZnO are directly reproducible in SnO_2_, Fe_2_O_3_, or NiO. Rather, the objective is to distinguish which effects are mechanistically transferable and which are strongly matrix-specific [2,25,31,32,76,77].

Accordingly, the assessment presented below should be understood as a framework of mechanistic transferability. It does not claim direct quantitative equivalence among oxide matrices, but systematically integrates crystallographic, defect-chemical, and interfacial knowledge from the literature. This approach helps identify which hypotheses should be prioritized in future experimental studies. It should also be emphasized that comparisons made without converting dopant content into atomic percentage, preserving equivalent thermal-treatment windows, and applying the same characterization chain may generate misleading equivalences across matrices [13,15,23].

The transferability discussion is therefore hypothesis-generating, not confirmatory. It identifies which mechanisms observed in ZnO may plausibly be tested in SnO_2_, Fe_2_O_3_, and NiO, but it does not claim that ZnO-derived trends have already been experimentally verified across all matrices under identical processing conditions. Any future extension should use matrix-specific control samples, equivalent cation loading, and the same characterization chain before drawing quantitative conclusions.

### 14.2. Transferability to the SnO_2_ Matrix: Dopant Behavior in a Rutile-Like n-Type Oxide

SnO_2_ is a rutile-structured and chemically stable n-type semiconductor that plays an important role in transparent conducting oxides, gas sensors, catalytic systems, and optoelectronic devices. In this matrix, boron doping presents a more complex crystal-chemical problem than in ZnO because B^3+^ does not match the Sn^4+^ site well in terms of either ionic size or valence. The literature suggests that a fraction of boron may be incorporated into the SnO_2_ lattice under limited conditions, whereas at higher concentrations it may tend toward segregation across surface, grain-boundary, or bulk-related regions. Nevertheless, in thin-film configurations, boron can improve carrier concentration and transparent conducting behavior. This indicates that boron-based strategies should not be dismissed in SnO_2_, but they should be interpreted not only through direct lattice substitution, but also through grain-boundary modification, defect compensation, and interface engineering [76,77,78].

The effect of rare-earth dopants in SnO_2_ proceeds more clearly through defect compensation, oxygen-vacancy regulation, and surface reactivity modification. CeO_2_, in particular, can influence surface redox behavior, adsorption activity, and gas-sensing sensitivity. However, the limited solubility of large ions such as Ce, La, or Y in the SnO_2_ lattice increases the risk of phase separation, surface segregation, and secondary rare-earth oxide formation. Therefore, although rare-earth-based strategies can generate functional advantages in selected SnO_2_ applications, the dopant concentration must be carefully controlled in order to preserve phase purity and microstructural homogeneity. The conclusion emphasized for ZnO, namely high modification capacity accompanied by a narrow processing window, is therefore largely transferable to SnO_2_ [31,32,77,78,79,80].

From a strategic perspective, the SnO_2_ case reinforces the central thesis of this study. Rare-earth dopants can improve selected functional parameters, but as the processing window becomes narrower, chemical heterogeneity and supply vulnerability increase simultaneously. By contrast, boron doping may not always deliver the highest functional leap, but it can offer a more manageable morphological response and a more defensible resource base, particularly in countries with domestic boron advantages. For a methodologically robust comparative study, these two outcomes must be demonstrated within the same experimental design. Otherwise, as frequently observed in the SnO_2_ literature, performance enhancement and structural cost remain disconnected within the same discussion [4,6,9,76,77,78,79,80].

### 14.3. Transferability to the Fe_2_O_3_ Matrix: Short Carrier Diffusion Length and Surface-State Problem

Fe_2_O_3_, particularly hematite, is one of the most extensively investigated oxides for photoelectrochemical water splitting, heterogeneous catalysis, and redox-based energy conversion. However, its performance is strongly governed by intrinsic electronic limitations of the matrix. Low electron and hole mobility, short carrier diffusion length, high probability of bulk and surface recombination, and kinetically active but electronically problematic surface states make hematite highly dependent on dopant engineering. Therefore, dopant selection in hematite cannot be explained merely by preservation of the primary phase or by small variations in lattice parameters. The central issue is the reorganization of energy barriers, trap states, and reaction-active surface sites encountered during carrier transport from the bulk to the surface. Within this context, boron doping emerges as a notable low-intensity intervention strategy for hematite because it can tune surface electronic structure at a finer scale, modulate the local bonding environment, and, under suitable conditions, limit disorder within the oxygen sublattice [81,82,83,84].

A common feature of boron-doped hematite studies is that low dopant levels are often associated with improved photoanodic current, facilitated charge-carrier separation, and reduced surface recombination. However, the origin of this improvement has not always been sufficiently distinguished. In many cases, it remains unclear how much of the observed performance enhancement originates from true intralattice B incorporation, how much arises from boron enrichment near the surface, and how much results from indirect redistribution of defect density. This uncertainty reflects a broader methodological problem frequently encountered in light-element doping studies. A boron signal detected by EDX cannot be defensibly interpreted as direct evidence of homogeneous lattice doping. In surface-dominated systems such as hematite, this limitation becomes even more critical because the active region is often not the bulk lattice, but the defect-rich near-surface region. Therefore, unless XPS depth profiling, Raman band shifts, complementary surface-sensitive chemical validation, and structure–performance correlations are evaluated together, definitive claims regarding the mechanism of boron doping carry a significant risk of overinterpretation [13,30,83,84].

Rare-earth dopants such as La and Ce generate a stronger and higher-impact, but also more fragile, intervention regime in hematite. In most cases, these ions do not exhibit complete and homogeneous solubility within the hematite lattice. Instead, they tend to accumulate at the surface, grain boundaries, or adjacent phase regions, thereby modifying redox behavior, oxygen-vacancy balance, and surface reactivity. This feature can create functional advantages, particularly in chemical looping, oxygen-carrier systems, and selected catalytic processes. However, the same mechanism may also generate adverse outcomes in photoanode architectures or fine-grained structures with high phase sensitivity, because local segregation, secondary-phase formation, and heterogeneous surface chemistry may introduce new recombination pathways even while improving selected charge-separation mechanisms. Therefore, the value of rare-earth dopants in hematite should be sought not simply in their stronger defect- and redox-engineering potential, but in the controllability of this potential. The conclusion is clear: for hematite, no universal proposition such as “rare-earth doping produces superior performance” can be established. The decision must depend on target application, dopant distribution, surface-state control, and the balance between phase stability and functional gain [81,82,85].

### 14.4. Transferability to the NiO Matrix: p-Type Defect Chemistry and Direction-Reversing Effects of Dopant Selection

Unlike ZnO and SnO_2_, NiO generally exhibits p-type behavior and possesses a defect chemistry strongly associated with hole conduction. Therefore, directly transferring dopant intuition developed for n-type oxides to NiO may be misleading. Nickel vacancies, excess oxygen, local valence ordering, and defect-induced acceptor states govern its electrical transport, optical response, and interfacial behavior. This means that dopants affect not only crystallographic parameters, but also the dominant carrier type, energy-level alignment, and charge-transport mechanism. Comprehensive evaluations of NiO-based sensors, electrochromic devices, and hole-transport layers indicate that morphological uniformity and defect chemistry must be optimized simultaneously [86,87,88].

The use of Y and related rare-earth dopants in NiO has produced promising results, particularly in terms of energy-level tuning, carrier concentration control, optical behavior, and thin-film quality. Nevertheless, the risks of ionic-size mismatch, segregation, and secondary-phase formation remain relevant in this matrix as well. The successful use of Y-doped NiO in selected optoelectronic applications indicates that rare-earth dopants cannot be categorically excluded from NiO-based systems. However, their beneficial effects often emerge within a narrow stoichiometric, oxygen-partial-pressure, and annealing window. In other words, in NiO, performance enhancement through rare-earth doping may be achieved, but often at the expense of increased process sensitivity [16,89].

The boron-based NiO literature is more fragmented than the corresponding ZnO and SnO_2_ literature. Nevertheless, existing studies show that boron doping can meaningfully modify the optical properties, resistive behavior, crystal parameters, and surface morphology of NiO thin films. Particularly at low and intermediate dopant levels, controllable changes in transmittance, grain size, surface roughness, and electrical response have been reported. This finding is important because it demonstrates that boron-based strategies are not limited to n-type oxides. When an appropriate processing window is available, they can also function as morphological, optical, and transport-tuning tools in p-type oxide systems [90,91,92].

The NiO system further sharpens the central conclusion of this study. Dopant selection cannot be considered independently of the electron- or hole-dominant carrier character of the host matrix. Rare-earth doping may be valuable in NiO for energy-level engineering and carrier modulation, whereas boron doping may be particularly useful for balancing surface morphology, transparency, and conductivity. However, the question of which strategy is “better” cannot be answered through a single numerical performance metric. Instead, it must be addressed by considering target application, processing tolerance, defect chemistry, and strategic supply conditions together. Direct comparative studies on NiO therefore represent an important research gap [16,86,87,88,89,90,91,92].

### 14.5. Synthesis of the Transferability Analysis

When the SnO_2_, Fe_2_O_3_, and NiO cases are considered together, a common pattern emerges. Boron-based dopants generally produce a more limited but more predictable structural intervention, whereas rare-earth dopants offer stronger potential for electronic, redox, and surface modification while carrying a higher risk of segregation, phase heterogeneity, and secondary-phase formation. This result shows that the ZnO-centered conclusion is not specific to a single material system, although it is not reproduced with the same magnitude in every oxide matrix. Therefore, the policy recommendation defended in this study is based not on a single host matrix, but on a broader strategic principle: in high-volume and scalable applications, boron-based dopants should serve as the default starting solution, whereas rare-earth dopants should be reserved for narrower target areas where a strong and non-substitutable functional justification can be established [4,6,9,10,11,12,13,16,77,78,79,80,81,82,83,84,85,86,87,88,89,90,91,92].

The transferability analysis also points to the need for a new reporting standard. In future studies, it should no longer be considered sufficient to state only that “dopant X improved the performance of matrix Y.” Instead, authors should explicitly discuss the mechanisms through which the observed improvement may or may not be transferable to other oxide families, as well as the conditions under which such transferability fails. This type of framework moves materials research beyond fragmented case reports and toward the production of generalizable knowledge. Scientific originality begins precisely at this point: the ability to make different islands of literature communicate through a common language of defect chemistry, morphology, interface formation, and resource policy [76,77,78,79,81,86].

The mechanistic comparison framework for the transferability of ZnO-centered findings to other oxide matrices is summarized in Table 9.

## 15. Deepened Interpretation of the Dopant Mechanism at Atomic, Crystallographic, and Interfacial Scales

### 15.1. Ionic Radius, Valence, and Defect Compensation

The most fundamental distinction between boron-based and rare-earth-based dopant strategies emerges from the scale and nature of their intervention within the crystal lattice. The B^3+^ ion acts as a much smaller and more strongly polarizing center than most transition-metal, post-transition-metal, or rare-earth cations. Therefore, in many oxide systems, boron modification proceeds either through limited substitution, interstitial positioning, local bonding rearrangement, or defect-associated complexes rather than through extensive lattice expansion. Its effect is often expressed through local bond-length adjustment, local coordination changes, and modulation of defect density, rather than through a coarse and spatially extended distortion of the entire lattice. By contrast, rare-earth ions such as Ce^3+^/Ce^4+^, La^3+^, and Y^3+^ impose larger crystallographic perturbations because of their ionic size, coordination preference, and oxide-forming tendency. Once the solubility limit is exceeded, these ions are more readily driven toward surface segregation, grain-boundary accumulation, or secondary-phase formation. For this reason, although both dopant families are often discussed under the general heading of “doping,” their crystallographic effects differ fundamentally in magnitude, spatial distribution, and structural consequence [2,25,30,31,32,76,77].

The issue of defect compensation further clarifies this distinction. When a dopant with a different valence state, ionic size, or coordination preference is introduced into a host lattice, the system attempts to preserve local charge neutrality and structural stability through the formation of vacancies, interstitial species, compensating valence changes, or rearranged bonding environments around neighboring atoms. In rare-earth-modified systems, oxygen-vacancy generation is frequently presented as a desirable outcome because it can increase surface-active sites and improve sensing, catalytic, photocatalytic, or redox-related functions. However, oxygen vacancies are not universally beneficial. At excessive concentrations or under poorly controlled distribution conditions, they may increase carrier scattering, form deep trap levels, accelerate recombination, and destabilize surface chemistry, especially when phase boundaries are irregular and dopant heterogeneity is high. In boron-modified systems, by contrast, changes in local bond stiffness, electron-density distribution, and short-range coordination may become more prominent than large-scale vacancy formation. This can produce more modest, but often more controllable, structural and electronic responses [2,25,30,33,34,76].

### 15.2. Crystal Strain, Grain Growth, and Sintering Kinetics

The core of morphological performance in doped oxide systems is closely linked to grain-growth kinetics. If an additive modifies grain-boundary mobility, alters interfacial energy, changes diffusion pathways, or promotes the formation of transient intermediate phases, the final grain size, grain-size distribution, pore connectivity, and surface morphology may change substantially. In boron-based systems, a commonly reported trend is that low additive levels can promote grain-boundary pinning, thereby leading to finer, more homogeneous, and more controlled microstructures. This effect becomes particularly evident in sol–gel, hydrothermal, solution-growth, and post-sputtering annealing routes, provided that the boron source or boron-containing component is well dispersed in the precursor environment. However, this trend should not be assumed to continue indefinitely. As boron content increases, intermediate species derived from B_4_C, B_2_O_3_, borate-like structures, or boron-rich interfacial domains may form, and the same system may begin to exhibit agglomeration, localized second-phase formation, or irregular microstructural evolution [33,34,47,48,49,50,51,52,53,54].

In rare-earth-modified systems, grain-growth dynamics are generally more dual and concentration-sensitive. At low dopant levels, rare-earth elements may facilitate nucleation, modify preferred orientation, suppress abnormal grain growth, or generate beneficial surface reactivity. However, as the dopant concentration increases, accumulation of large ions at grain boundaries, local chemical heterogeneity, modified sintering behavior, and coarse cluster formation through heterogeneous nucleation become more likely. Therefore, it is not sufficient to state from FE-SEM images that “the particles became larger” or “the particles became smaller.” Grain-size distribution, circularity, pore connectivity, surface roughness, agglomeration index, and clustering degree should be reported quantitatively. Methodologically robust studies should discuss morphology not on the basis of a single selected micrograph, but through statistical image analysis, multi-area sampling, and, where possible, complementary surface-area or porosity measurements [33,34,35,36,43,44,45,55,56,57,58,59,60,61,62,63,64,65,76].

### 15.3. Surface Chemistry, Adsorption Centers, and Electronic Structure

In advanced oxide materials, the link between structural modification and application performance is often established through surface chemistry. In gas sensing, photocatalysis, photoelectrochemistry, and electrochemical energy storage, the active region is not an ideal infinite crystal, but a real surface containing defects, hydroxyl groups, chemisorbed oxygen species, local coordination irregularities, and adsorbate interactions. Rare-earth dopants can generate strong advantages at this surface through redox couples, variable valence states, oxygen storage/release capability, and modified adsorption–desorption kinetics. This is why Ce-based dopants are particularly important in systems where surface oxygen exchange and redox cycling govern the functional response. However, the same mechanism can also increase chemical heterogeneity, promote non-uniform reactive domains, and create long-term stability problems if the dopant distribution is not controlled [35,38,43,55,56,57,58,59,68,77,80].

The surface-chemical role of boron-based dopants is generally more subtle. In most cases, boron does not function as a strong redox center. Instead, it modifies local bonding character, acid–base surface properties, charge-density distribution, and, in some matrices, the balance between surface defects and structural order. When h-BN or other boron-containing interfacial supports are used, the strategy extends beyond chemical doping and enters the domain of heterointerface construction. Such interfaces may promote charge separation, improve particle dispersion, guide nucleation, stabilize surface morphology, and support phonon or heat dissipation. In this context, boron-based dopant strategies represent a spectrum extending from single-atom-level defect tuning to secondary interfacial architecture. Correctly interpreting this spectrum requires Raman and XPS data to be analyzed not only in terms of peak presence or absence, but also through peak shifts, band broadening, shoulder formation, chemical-state ratios, and their consistency with structural and morphological evidence [29,37,40,41,42].

### 15.4. Integrated Interpretation of XRD, XPS, Raman, and FT-IR Data

A robust understanding of dopant mechanisms cannot be established by relying on a single characterization technique. XRD can reveal preservation of the primary phase, secondary crystalline phases, lattice-parameter changes, crystallite-size trends, peak broadening, and microstrain. However, low-level dopants, amorphous secondary phases, surface-enriched species, and nanometer-scale segregated domains may remain below the detection limit of XRD. XPS provides information on chemical states, surface atomic percentages, bonding environments, and oxygen-related surface components, but its information depth is limited to the near-surface region. Raman spectroscopy can detect lattice distortions, defect-related band broadening, phonon-mode shifts, and minor phase contributions that may not be clearly resolved in XRD. FT-IR offers complementary evidence regarding surface hydroxylation, borate-like groups, B–N or B–O bonding, metal–oxygen vibrations, and interfacial bonding interactions in composite systems. A scientifically convincing interpretation therefore emerges only when the overlapping and divergent information provided by these techniques is evaluated together [13,25,29,30,76,77].

For example, if only a slight XRD peak shift is observed after doping, whereas XPS reveals a clear change in dopant chemical state or surface composition, the result may indicate surface enrichment, grain-boundary accumulation, or near-surface defect modification rather than homogeneous lattice substitution. Similarly, if Raman spectra show increased defect-related band intensity or phonon broadening while XRD indicates increased crystallite size, the dopant may be simultaneously influencing nucleation, strain relaxation, and surface disorder. Therefore, the methodologically expected approach is not to present isolated conclusions for each technique, but to develop a cross-validation logic among phase analysis, chemical-state analysis, vibrational spectroscopy, and morphology. This integrative interpretation is indispensable for light elements such as boron and for multivalent dopants such as Ce, where misleading conclusions can easily arise from single-technique evidence [30,35,43,68,77,80,93].

### 15.5. Threshold Behavior of Beneficial and Detrimental Mechanisms

A common mistake in interpreting dopant strategies is to assume that a beneficial effect observed at low concentration will continue to increase without limit. In practice, both boron-based and rare-earth-based dopants usually exhibit threshold behavior. At low and intermediate dopant levels, beneficial mechanisms may dominate, including enhanced nucleation control, defect tuning, optimized surface-active centers, improved charge separation, energy-level adjustment, grain-boundary stabilization, or interfacial charge transfer. Beyond a critical concentration, however, detrimental mechanisms may become dominant. These may include phase separation, surface segregation, carrier scattering, excessive trap-state formation, pore blockage, optical scattering, structural disorder, agglomeration, or loss of surface homogeneity. The true value of a dopant strategy is therefore not determined only by its maximum reported performance, but by the width, reproducibility, and process tolerance of its beneficial operating window [24,25,26,27,28,29,37,61,83].

This perspective is fully consistent with the strategic conclusion of the study. Rare-earth dopants may sometimes deliver higher peak performance because of their strong redox activity, oxygen-vacancy modulation, and electronic-state engineering. However, they become more fragile options from both industrial and policy perspectives when their beneficial window is narrow, their process sensitivity is high, and their supply profile is vulnerable. Boron-based dopants, by contrast, may generate higher overall technological value if they provide broader processing tolerance and more reproducible structural behavior, even when their absolute peak performance is slightly lower. Therefore, future direct comparative studies should report not only the optimum dopant ratio, but also the width of the optimization window, the onset of degradation mechanisms, and the reproducibility of the beneficial response. The distinctive methodological strength of dopant studies emerges precisely at this point [4,9,13,70,71,72,73,74,75,76,77].

The dominant mechanisms, expected characterization signatures, and primary risks associated with boron- and rare-earth-based dopants are summarized in Table 10.

## 16. Statistical and Methodological Strengthening of the Direct Comparative Experimental Design

### 16.1. Principle of True Comparability Within the Same Matrix

Although boron-based and rare-earth-based dopants are frequently compared in the literature, a considerable portion of these comparisons is not methodologically equivalent. In one study, boron doping may be carried out through a sol–gel route followed by annealing at 450 °C, whereas in another study, CeO_2_ incorporation may be achieved through a hydrothermal route followed by annealing at 600 °C. When the resulting differences in crystallinity, phase formation, grain size, or surface morphology are attributed solely to dopant chemistry, the interpretation becomes methodologically fragile. Parameters such as precursor chemistry, precursor purity, solution pH, solvent system, aging time, drying kinetics, annealing rate, annealing atmosphere, and substrate type may influence morphology and defect chemistry as strongly as the dopant itself. Therefore, a robust comparative study should attempt to isolate dopant chemistry as the principal independent variable while keeping the synthesis regime as constant as possible [13,15,23,33,34].

Accordingly, three principles are proposed for future direct comparative studies. First, dopant content should, wherever possible, be reported on the basis of atomic percentage, molar percentage, or cation molar ratio, because these descriptors provide a more chemically meaningful basis for comparing light elements such as boron with heavier rare-earth elements. Weight percentage may also be reported in the main text for practical reproducibility, but it should not be used as the only concentration descriptor in comparative dopant studies. Second, the undoped reference sample, boron-based modified samples, and rare-earth-modified samples should be prepared using identical precursor purities, equivalent precursor concentrations, the same solvent chemistry, and the same heat-treatment window. Third, the characterization chain should be applied symmetrically to all samples. For instance, performing XPS only on the sample that exhibits the highest apparent performance disrupts the evidential balance of a comparative study and weakens causal interpretation. Although these principles may appear straightforward, this level of methodological consistency is precisely what determines the reliability, comparability, and publication quality of direct dopant-comparison studies [13,30,31,76,77].

### 16.2. Verification of Dopant Distribution: Avoiding Assumptions Beyond Imaging Evidence

Verification of dopant distribution remains one of the weakest points in many doped oxide studies, particularly for light elements and low dopant concentrations. Although EDX maps may appear visually convincing, their sensitivity to boron is limited, and their spatial resolution in fine-grained systems is insufficient to prove true compositional homogeneity or lattice incorporation. For rare-earth elements, elemental signals are easier to detect; however, a different interpretive risk emerges. Dopant enrichment at the surface, grain boundaries, or within coarse clusters may be mistakenly interpreted as homogeneous distribution throughout the entire structure. Therefore, EDX mapping, point analysis, line scanning, XPS-derived surface atomic percentages, and multi-area FE-SEM observations at different magnifications should be evaluated together rather than independently [13,30,31,76,77].

A methodologically defensible approach requires that every claim regarding dopant distribution be supported by at least two independent lines of evidence. For example, a statement such as “boron is homogeneously distributed” should not be based on a single EDX map. It should be supported by a reliable B-related XPS signal, XRD evidence indicating the absence of detectable phase segregation, and FE-SEM observations showing limited clustering or morphological discontinuity. Similarly, in rare-earth-modified systems, a claim of homogeneous distribution should be verified not merely by the presence of a strong Ce, La, or Y signal, but also by the absence of secondary-phase peaks, consistent elemental ratios across multiple regions, and the lack of localized rare-earth-rich clusters in mapping data. Without such cross-validation, the study may appear visually persuasive while remaining analytically weak [13,35,38,43,44,45,64].

### 16.3. Quantification of Morphology and Statistical Power

A substantial part of the morphological discussion in doped oxide studies still relies on qualitative descriptors such as “more homogeneous,” “less agglomerated,” “smoother,” or “more porous.” When such statements are not supported by quantitative criteria, their evidential strength remains limited. Current publication standards increasingly require, at minimum, reporting particle or grain-size distribution, mean and median values, standard deviation or variance, the number of analyzed image fields, the measurement protocol, and, where relevant, image-processing parameters. For agglomeration, metrics such as clustering percentage, particle contact density, nearest-neighbor distance, or agglomerate area fraction may be used. For porosity-related morphology, area fraction, pore connectivity, and apparent pore-size distribution may be reported, while gray-level texture metrics can provide additional information on surface homogeneity [13,26,27,31,32].

Statistical power does not simply mean increasing the number of measured particles. It requires a scientifically appropriate sampling design. Treating multiple particles measured from the same selected micrograph as fully independent samples may lead to pseudoreplication and artificial inflation of statistical confidence. Instead, sampling should include different regions of the same specimen, multiple independently prepared samples where possible, and a blinded or semi-automated measurement protocol to reduce selection bias. A convincing comparative study should clearly report the number of independent synthesis replicates, the number of image fields analyzed for each replicate, the total number of particles or grains measured, the criteria used for excluding ambiguous features, and the statistical tests applied for comparison. This approach is essential for distinguishing whether the observed differences between boron- and rare-earth-modified systems genuinely originate from dopant chemistry or merely reflect random morphological variability [13,26,27].

### 16.4. Causality Chain Across Characterization Techniques

One of the most common problems in structural and functional materials research is the conflation of correlation with causality. For example, if a Ce-modified ZnO sample exhibits a higher gas-sensing response, the improvement may be directly attributed to “increased oxygen vacancies.” However, grain size, porosity, surface hydroxylation, phase heterogeneity, and adsorption-site distribution may also have changed simultaneously. Similarly, if photocatalytic activity increases after boron doping, the cause may not be limited to band-gap modification; it may also involve improved dispersion, higher accessible surface area, reduced recombination, modified surface chemistry, or suppressed agglomeration. Therefore, the experimental design should establish complementary measurement sets rather than deriving a single causal explanation from a single measurement [25,26,30,38,43,57,68,80,93].

The proposed causality chain can be organized as follows: first, phase integrity, crystallite size, lattice parameters, and microstrain should be evaluated by XRD; second, morphology, grain distribution, surface continuity, and agglomeration should be examined by FE-SEM; third, dopant distribution and local compositional variation should be assessed by EDX mapping and line scanning; fourth, chemical state, surface composition, defect-related oxygen components, and dopant bonding environments should be evaluated by XPS; fifth, vibrational, bonding, and defect signatures should be examined by Raman and FT-IR spectroscopy; and finally, application performance should be measured and interpreted in relation to the preceding structural and chemical evidence. This sequence makes it possible to ground performance enhancement in upstream structural, morphological, and chemical changes rather than treating performance as an isolated outcome [13,30,31,76,77,80].

### 16.5. Reporting Standard, Reproducibility, and Methodological Robustness

Under current scientific standards, not only the results themselves but also the organization and transparency of the evidence package determine the strength of a study. Therefore, a minimum reporting standard can be proposed for comparative dopant investigations. The synthesis recipe should be reported at the molar level; dopant loading should be provided as both nominal and measured values where possible; precursor purity, solvent system, pH, aging time, drying conditions, annealing temperature, heating rate, dwell time, and atmosphere should be explicitly stated; the operating conditions of all characterization instruments should be specified; the morphological quantification protocol should be described; and negative or unexpected results should not be omitted from the manuscript. This final point is particularly important because the true dopant window is often defined not only by successful samples, but also by the onset of deterioration, phase segregation, agglomeration, or performance decline [13,15,23].

The methodologically strongest study is not necessarily the one that presents the largest number of measurements, but the one that establishes a coherent internal logic among synthesis conditions, characterization evidence, statistical treatment, and functional performance. Only through this logic can the broader thesis constructed between boron-based and rare-earth-based dopant strategies be defended convincingly. The experimental standardization proposed here would ensure that future original datasets are not only more reliable, but also more readily connectable to public policy discussions. Non-reproducible performance results provide a weak basis for defining national R&D priorities; strategic materials governance can only be built on methodologically robust materials science [4,13,70,71,72,73,74,75].

The proposed minimum experimental and reporting standard for the direct comparison of boron- and rare-earth-based dopants is summarized in Table 11.

## 17. Multi-Criteria Decision-Making Framework: Technical Performance, Supply Security, and Policy Alignment

### 17.1. Insufficiency of a Single Performance Criterion

Material dopant selection is frequently reduced in the literature to a single performance indicator, such as higher gas sensitivity, lower band-gap energy, improved photocatalytic rate constant, enhanced photoluminescence intensity, or increased capacitance. Although such metrics are valuable for evaluating application-specific functionality, they are insufficient for strategic materials research. A dopant that provides a superior laboratory-scale response may reduce the overall technological value of a material system if it is associated with high supply risk, narrow processing tolerance, limited scalability, difficult recycling, high price volatility, or weak compatibility with the domestic value chain. For this reason, the framework proposed in this study does not evaluate dopant selection solely through material performance, but through the integrated assessment of technical, economic, process-related, and geostrategic variables [4,5,6,7,8,9,10,11,12,13,14,71,72,73,74,75].

The multi-criteria decision-making approach shifts the comparison between boron- and rare-earth-based dopants from an absolute ranking logic to a context-sensitive evaluation framework. This is necessary because the two dopant families generate different advantages and different strategic costs. Boron-based dopants generally offer broader processing tolerance, stronger compatibility with domestic resource availability, lower supply-chain vulnerability, and, in many cases, a more predictable structural and morphological response. Rare-earth dopants, in contrast, can provide stronger optoelectronic, redox, luminescent, or surface-reactive modification in selected application windows. Therefore, dopant selection should not be based on a context-independent statement of superiority. It should instead be evaluated through scenario-based rationality, in which the preferred dopant depends on the relative importance of technical performance, manufacturability, and strategic alignment [4,6,9,13,71,72,73,74,75].

### 17.2. Proposed Evaluation Criteria and Weighting Logic

The decision model proposed in this study is built on three main pillars. The first is the technical pillar, which includes phase purity, risk of secondary-phase formation, agglomeration tendency, grain-size control, surface homogeneity, defect-management capacity, interfacial functionality, and the target application output. The second is the manufacturability pillar, which includes synthesis complexity, processing-window width, reproducibility, scalability, precursor availability, process tolerance, and quality-control difficulty. The third is the strategic pillar, which includes supply security, import dependence, price volatility, refined-product concentration, recycling potential, substitution potential, and alignment with the national resource base. The relative weights of these pillars may vary depending on the research context; however, an evaluation system that scores only the technical pillar is not compatible with the materials-governance perspective advanced in this article [10,11,12,13,14,71,72,73,74,75].

In an academic discovery-oriented scenario, the technical pillar may be assigned the highest weight. For example, 55% of the total score may be derived from technical performance, 20% from manufacturability, and 25% from strategic considerations. In an industrial process-development scenario, however, manufacturability and reproducibility become more decisive, and the weight of the manufacturability pillar should increase. In national R&D prioritization, the strategic pillar should receive a stronger coefficient because supply security, domestic resource advantage, scalability, and value-chain development become central decision variables. This flexible weighting logic explains why boron- and rare-earth-based dopants may lead to different “best choices” for different actors. A high-impact Ce-modified ZnO result may be attractive for an academic laboratory focused on demonstrating a new mechanism, whereas a boron-based platform may be more rational for a public technology program seeking scalable, reproducible, and strategically secure material solutions [10,11,12,13,14,71,72,73,74,75].

### 17.3. Scenario-Based Outcomes: Academic, Industrial, and Public Perspectives

In the academic discovery scenario, the primary objective is often to demonstrate a new mechanism, reveal a strong structure–property relationship, or achieve a pronounced increase in a selected functional metric. Under these conditions, rare-earth dopants may receive high scores, particularly in subfields where photoluminescence, gas-sensor selectivity, oxygen-vacancy modulation, or surface redox capability is critical. Ce-, La-, or Y-based dopants can provide distinct property modification within specific application windows. However, even in this scenario, rare-earth dopants do not automatically become the preferred option. If phase purity, dopant distribution, and morphological homogeneity are not adequately preserved or verified, the generalizability and mechanistic reliability of the results decrease. Therefore, strong characterization evidence and a clear causal link between dopant chemistry and functional output remain essential [31,32,33,34,35,36,43,44,45,55,56,57,58,59,60,61,62,63,64,65,66,67,68,77].

In the industrial process-development scenario, the evaluation logic changes substantially. Production reproducibility, low batch-to-batch variation, continuous raw-material access, broad processing tolerance, scalable synthesis conditions, and manageable quality-control requirements become decisive. Under these conditions, boron-based dopants often achieve a higher overall score because they can offer lower strategic vulnerability, more manageable microstructural evolution, and better compatibility with high-volume processing. Rare-earth-modified systems may remain valuable in specialized niche products where their functionality is indispensable, but their process sensitivity, phase-complexity risk, and supply volatility may be less acceptable in large-scale production lines. This distinction demonstrates that “high peak performance” and “high technological maturity” are not equivalent concepts in material selection [4,6,9,10,11,12,13].

From the public-policy perspective, the dominant question is whether scientific evidence can be transformed into a technology portfolio that reduces strategic dependence and strengthens domestic value creation. For a country such as Türkiye, which possesses a strong boron-resource advantage, this does not mean that boron-based dopant strategies should be treated as the only legitimate option for all applications. However, it does justify their default prioritization in broad public funding programs when they provide sufficient or comparable technical performance. Rare-earth-based pathways should be supported more selectively, particularly in high-value-added applications where their functional contribution is clearly indispensable and cannot be replicated by boron-based or more accessible alternatives. In this way, public policy can establish a more balanced portfolio between functional ambition and strategic resilience [10,11,12,13,14,71,72,73,74,75].

### 17.4. Sensitivity Analysis and Interpretation of Decision Thresholds

One of the most important advantages of multi-criteria models is that they reveal which assumptions drive the final decision. For example, when the weight of the strategic pillar is increased from 20% to 35%, boron-based dopants are expected to move upward in the overall ranking because of their stronger supply-security and domestic value-chain profile. Conversely, if the technical pillar is weighted excessively and only the highest laboratory-scale performance metric is rewarded, rare-earth-modified systems may become more favorable in selected applications. This shift demonstrates that the materials debate is not purely technical; it is also normative. The value assigned to peak performance, reproducibility, scalability, supply security, or domestic industrial relevance determines which dopant is regarded as “superior.” A methodologically strong study should therefore make this normative layer explicit rather than hiding it behind a single performance ranking [13,71,72,73,74,75].

For this reason, the decision approach proposed in this article advocates the reporting of decision thresholds and scenario shifts rather than the declaration of a single absolute score. A rare-earth dopant should be considered rational when its technical superiority over a boron-based alternative exceeds a defined threshold and when this superiority is not accompanied by unacceptable losses in reproducibility, process tolerance, or supply security. Similarly, the default prioritization of boron-based dopants should not rely solely on the domestic-resource argument. It should also be conditional on achieving a sufficient technical-performance threshold for the intended application. This structure enables the decision model to incorporate strategic reasoning without compromising scientific quality [4,9,10,11,12,13,14].

### 17.5. Outcomes of the Decision Model in This Article

The evidence package integrated in this study produces three main outcomes. First, from the perspective of structural and morphological stability, boron-based dopants constitute a more reliable baseline strategy, particularly at low and intermediate dopant levels where phase preservation, lower agglomeration tendency, and more predictable microstructural evolution are frequently observed. Second, although rare-earth dopants may offer higher functional-gain potential in selected metrics such as redox activity, luminescence, oxygen-vacancy modulation, or surface reactivity, this advantage often comes with a narrower processing window, greater phase-complexity risk, and higher supply dependence. Third, within the logic of public funding, it is not rational for the technical score alone to determine prioritization. Total technological value becomes meaningful only when functional performance, manufacturability, reproducibility, scalability, and strategic vulnerability are evaluated together [4,5,6,7,8,9,10,11,12,13,14,31,32,71,72,73,74,75].

Accordingly, the multi-criteria framework developed here does not produce a simplistic conclusion that one dopant family is universally superior to the other. Instead, it defines the conditions under which each family becomes rational. Boron-based dopants are positioned as broad-platform candidates for scalable and strategically secure material development, whereas rare-earth dopants are positioned as high-functionality candidates for selected applications in which their unique electronic, optical, or redox roles are demonstrably necessary. This distinction provides a more realistic and policy-relevant basis for dopant selection than a conventional comparison based only on peak laboratory performance.

**Figure 3:**
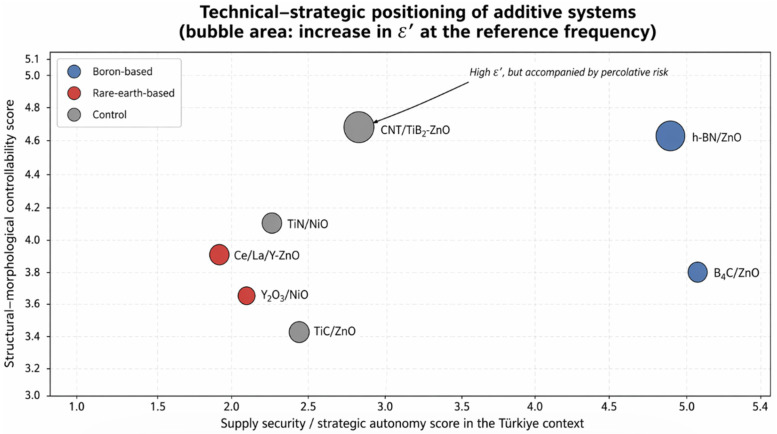
Technical–strategic positioning of selected additive systems; the bubble area represents relative dielectric enhancement at the reference frequency. The map is scenario-based and should be interpreted together with the measurement anchors and synthesis limits reported in the comparative tables, rather than as a fully normalized cross-matrix ranking.

The technical and strategic positioning map, in which technical controllability, supply security, and dielectric enhancement potential are considered together, is presented in Figure 3 [5,10,11,12,13,14,15,16,17,18,19,20,21,22,33,34,35,36,37,38,39,40,41,42,43,44,45].

This approach is also academically productive, because it allows for research programs to be differentiated more consciously. If the aim is to address high-impact fundamental science questions, mechanism-oriented studies on selected rare-earth-doped systems can be supported. If the aim is to develop a national materials platform, a standardized, reproducible, and scalable research pathway should be established through boron-based dopants. The strongest publication strategy is not to position these two pathways in opposition, but to analytically demonstrate the decision threshold at which one should be preferred over the other [10,11,12,13,14,71,72,73,74,75].

The scenario-based multi-criteria decision-making framework and the relative positions of the dopant families are summarized in Table 12.

## 18. Global Critical Raw Material Regimes and Strategic Materials Governance

### 18.1. Transition from the Critical Raw Material Concept to Materials Design

The critical raw material debate has long been addressed as a macroeconomic and geopolitical issue involving supply concentration, foreign trade restrictions, price volatility, and vulnerability in strategic sectors. However, from the perspective of advanced materials research, the more difficult and productive question is how these macro-level risks should be translated into laboratory-scale material design decisions. If a dopant family is associated with geopolitically concentrated supply, export restrictions, limited refining capacity, and strong dependence on external processing infrastructure, then using that dopant as a default option in laboratory research is not merely an economic choice. It also represents an institutional decision that may render the future research and technology portfolio vulnerable to external shocks. Therefore, a direct conceptual bridge must be established between critical raw material studies and dopant engineering [4,5,6,7,8,9,13,14,71,72,73,74,75].

Rare-earth elements constitute the most visible example of this connection. The international literature shows that rare-earth supply chains possess a risk profile distinct from many conventional metals because of geographical concentration, the clustering of separation and processing capacity in specific countries, and sensitivity to trade policies. By contrast, although boron is not a universally abundant raw material available to all economies on equal terms, it occupies a different geoeconomic position for Türkiye because of the country’s substantial reserves, export capacity, and institutionalized production infrastructure. This situation makes it possible to regard boron-based dopants not only as technical alternatives, but also as potential pathways for strategic capacity building in advanced materials [10,11,12,71,72,75].

### 18.2. European Union, OECD, and Global Policy Framework

In recent years, the European Union has developed a more explicit institutional framework for critical raw materials, focusing on supply diversification, recycling, protection of strategic technology sectors, domestic processing capacity, and reduction in external dependence. This framework treats not only mining, but also refining, processing, intermediate products, and secondary raw material cycles as strategically important components of material security. OECD-oriented analyses and related international policy studies likewise emphasize that export restrictions, concentrated production networks, and limited processing capacity create significant vulnerabilities for technologies associated with the green transition, digital transformation, and advanced manufacturing. This perspective conveys a clear message for materials research: dopant selection cannot be made with supply-chain blindness. Otherwise, laboratory-scale success may fail to become a sustainable and scalable technology at the industrial level [5,13,14,72,73,74,75].

This policy context shifts the comparison between boron- and rare-earth-based dopants onto a new evaluation plane. Rare-earth-based dopant strategies may be scientifically rational in applications such as highly selective gas sensors, specialized optical functions, advanced redox-active photocatalysts, or luminescent systems. At the policy level, however, these strategies should be justified primarily when they provide a function that is difficult to substitute. By contrast, once boron-based dopants become technically “sufficiently” successful within a given application window, they move rapidly upward in the strategic preference ranking. Within the same or a comparable performance band, an option that offers lower external dependence, stronger domestic alignment, and greater value-chain integration has a more robust justification within the logic of public R&D funding [10,11,12,13,14,71,72,73,74,75].

### 18.3. Strategic Value and Limitations of Boron in the Context of Türkiye

From Türkiye’s perspective, the strategic value of boron should be understood on three levels beyond the existence of raw reserves. The first is the geological and commercial level, where Türkiye possesses a strong resource base and a significant position in boron mineral production and export. The second is the industrial level, where accumulated institutional know-how extending from boron mining to processed boron products provides a different starting point compared with the external processing dependence observed in rare-earth supply chains. The third is the science-policy level, where domestic resource advantage can only be transformed into strategic advantage if R&D programs are established to connect boron resources with high-value-added advanced materials technologies. Therefore, boron’s strategic advantage is not automatic; it is a potential that must be activated through policy, research infrastructure, and industrial translation [10,11,12]. At the same time, the strategic position of boron should not be overstated. The fact that boron is domestically accessible and abundant does not mean that every boron derivative will automatically be the optimum dopant for every application. Moreover, import dependence may still exist for certain high-purity precursors, specialized thin-film chemicals, processing additives, or intermediate products required for composite and interface architectures. Strategic maturity is achieved not by romanticizing domestic resources, but by transforming resource advantage into reproducible, high-value-added, and application-relevant materials design. This is precisely the priority advanced in this study: the existing boron advantage should be transferred into the advanced oxide ecosystem through selective, evidence-based, and scalable research programs [10,11,12,71].

### 18.4. Strategic Vulnerability and Justification Threshold for Rare-Earth Dopants

For rare-earth dopants, the fundamental issue is not the absence of scientific value, but the existence of a higher strategic justification threshold. These dopants can and should be used where they provide unique functionality. However, their use becomes strategically defensible only when the technical benefit is clear, reproducible, difficult to substitute, and sufficiently strong to compensate for supply-chain vulnerability. Placing an input characterized by external dependence, price volatility, and uncertain access at the center of a research portfolio is not merely a laboratory cost; it also anchors future industrialization pathways to externally controlled material flows. Therefore, for rare-earth-modified studies to receive priority in public funding, the expected benefit should go beyond a routine incremental improvement and should instead correspond to a function that is difficult to reproduce using more accessible alternatives [4,5,6,7,8,9,71,72,73,74,75].

This threshold-based approach does not restrict scientific research; rather, it makes the rationale for dopant selection more transparent. If a Ce-, La-, or Y-modified system provides unique photoluminescence behavior, gas selectivity, charge-separation efficiency, redox architecture, or energy-level tuning, its use can be strongly justified from both academic and technological perspectives. By contrast, if the same or a comparable function can be achieved with a boron-based, undoped, or more accessible material system, the strategic justification for selecting a rare-earth dopant becomes weaker. A methodologically robust discussion therefore requires testing dopant indispensability through counterfactual substitution scenarios: what would be lost technically if the rare-earth dopant were replaced by boron-based or lower-risk alternatives? [4,6,9,13,31,32,77].

### 18.5. Policy Implications for Public R&D Prioritization

In this formulation, policy preference is activated only after the material system satisfies a technical-performance threshold. Domestic resource availability does not substitute for phase stability, reproducible morphology, validated incorporation mode, or application-level function; rather, it becomes decisive when two technically credible routes occupy a comparable performance window. A technically credible route is defined here by a minimum evidence package: phase and crystallite response from XRD, morphology and agglomeration assessed by multi-field imaging, dopant/secondary-phase assignment supported by EDX together with XPS/Raman/FT-IR where required, and functional data reported with frequency, loading, or application-test anchors. This explicitly prevents strategic-resource reasoning from being detached from the material evidence on which it must depend.

From the perspective of public R&D incentives, the most direct recommendation of this study is to structure the research portfolio in two complementary layers. The first layer should consist of high-volume, scalable, and platform-oriented research based on boron-containing dopant strategies. The objective should be to establish standardized synthesis–structure–property relationships, quality protocols, characterization standards, and processing windows in matrices such as ZnO, SnO_2_, Fe_2_O_3_, and NiO. Through this layer, Türkiye’s domestic boron-resource advantage could be transformed into reproducible technological infrastructure rather than remaining limited to raw material export or isolated laboratory studies.

The second layer should be reserved for selected rare-earth-modified projects. These projects should be supported when they demonstrate a clear mechanistic advantage, a non-substitutable functional output, and, where possible, a recovery, recycling, substitution, or reduced-loading strategy. This structure would allow rare-earth dopants to remain within the national research portfolio without allowing them to become default choices in areas where boron-based or other accessible alternatives can provide sufficient performance [10,11,12,13,14,71,72,73,74,75].

This two-tier model makes it possible to preserve scientific diversity while reducing strategic dependence. It also enables laboratory-scale data to be connected more explicitly with industrial policy, value-chain planning, and technology-economy pathways. Consequently, dopant selection should be evaluated not only as a matter of materials chemistry, but also as part of the national innovation system, strategic resource governance, and long-term technological sovereignty [4,5,6,7,8,9,10,11,12,13,14,71,72,73,74,75].

The policy profiles of boron-based and rare-earth-based dopant families in terms of critical raw material governance are summarized in Table 13.

## 19. Industrial Scale-Up, Technology Readiness Level, and Circularity Perspective

### 19.1. Reframing Dopant Strategy During the Laboratory-to-Pilot-Scale Transition

A dopant strategy that appears successful at the laboratory scale should not be assumed to retain the same level of performance, homogeneity, or reproducibility when transferred to pilot or industrial scale. This issue is particularly important in solution-based synthesis routes, where precursor homogeneity, mixing intensity, drying kinetics, local concentration gradients, and temperature distribution within the annealing furnace become increasingly difficult to control as batch volume increases. Under such conditions, dopant distribution, phase evolution, agglomeration behavior, and surface morphology may deviate substantially from those observed in small laboratory specimens. Therefore, the true value of a dopant strategy should not be measured solely by the peak performance obtained in a small-scale sample, but also by the stability of that performance under scale-up conditions. In this regard, boron-based dopants may often provide an advantage because of their broader processing tolerance and comparatively lower tendency toward severe segregation, whereas even minor process deviations in rare-earth-modified systems may lead to pronounced chemical and morphological heterogeneity [4,6,9,13].

As the technology readiness level increases, the required evidence standard also changes. At the level of fundamental research, a proof-of-concept result supported by XRD, FE-SEM, and selected spectroscopic evidence may be sufficient to demonstrate scientific feasibility. For pilot-scale implementation, however, additional requirements emerge, including batch-to-batch consistency, raw material specifications, processing-window mapping, quality-control thresholds, accelerated aging stability, environmental durability, and reproducible performance under realistic operating conditions. From this perspective, the industrial maturity of a dopant strategy is not a direct extension of laboratory performance; it constitutes a separate research and validation problem. This distinction is also important for the policy dimension of the present study. Public funding mechanisms should not conflate dopant studies that provide only laboratory-scale characterization with claims of technological readiness or industrial applicability [13,71,72,73,74,75].

### 19.2. Precursor Chemistry, Purity Requirements, and Quality Control

The industrial significance of dopant-family selection becomes particularly concrete in the purity, reactivity, and quality-control requirements of the precursors used. Boron-based strategies may involve boric acid, borate salts, B_4_C, h-BN, boron nitride quantum dots, or other boron-containing intermediates, each of which possesses distinct purity levels, particle-size distributions, surface chemistry, and reactivity profiles. On the rare-earth side, the hydration state, carbonate contamination, actual oxide equivalent, dissolution behavior, precursor aging, and conversion efficiency of Ce-, La-, and Y-based salts or oxides can produce stronger batch-to-batch variability. These differences may cause substantial fluctuations in the final microstructure, even when the nominal synthesis parameters appear identical in laboratory records. Therefore, during scale-up, precursor standardization becomes as critical as dopant chemistry itself [4,6,9,10,11,12]. From a quality-control perspective, the analytical difficulty of detecting light elements in boron-based systems creates a distinct challenge. However, this challenge is primarily methodological and can be managed through an appropriately designed measurement strategy involving XPS, Raman spectroscopy, FT-IR, and carefully interpreted elemental analysis. In rare-earth-modified systems, elemental verification is generally easier because Ce, La, and Y can be detected more readily by EDX or XPS. Yet the main difficulty shifts toward maintaining phase integrity, suppressing segregation, and ensuring homogeneous dopant distribution across the scaled-up batch. In other words, analytical verification is the dominant issue for boron-based systems, whereas process stability and phase homogeneity are the dominant issues for rare-earth-based systems. A strong technology-development program should distinguish between these two challenges and allocate its characterization, process-control, and quality-assurance resources accordingly [13,31,32,76,77].

### 19.3. Circularity, Material Recovery, and Waste-Oriented Design

In an era in which critical raw material policies are becoming increasingly sophisticated, evaluating a dopant strategy only on the basis of first-use performance is no longer sufficient. The recoverability of the material throughout its life cycle, its behavior in waste streams, its compatibility with recycling infrastructure, and its potential transformation into secondary raw materials must also be considered part of its strategic value. For rare-earth elements, recovery and recycling have long been central topics in circular economy discussions. However, the literature indicates that rare-earth recovery remains technically complex, economically selective, and strongly dependent on product architecture. In low-concentration, highly dispersed, and multicomponent matrices, rare-earth recovery can be costly, energy-intensive, and chemically demanding. Therefore, the overall sustainability profile of rare-earth-doped systems cannot be considered favorable solely because they deliver enhanced functional performance [72,73,74].

Boron-based dopants may offer a more favorable circularity profile in certain applications, although excessive optimism should also be avoided. Depending on the host matrix, dopant concentration, final product form, and end-of-life route, boron-containing systems may still create specific challenges for recovery, separation, or reuse. Nevertheless, the strategically important distinction is that, for boron-based systems in a producer country such as Türkiye, the institutional distance between renewed access to primary domestic resources and the development of recovery capacity is comparatively shorter. In rare-earth-based systems, by contrast, both primary supply and secondary recovery may simultaneously involve complex international dependencies, specialized separation technologies, and uncertain economic feasibility. Thus, the circularity perspective adds another layer of justification for prioritizing boron-based research pathways in broad public R&D programs, while positioning rare-earth-containing systems within more selective and functionally indispensable application windows [10,11,12,72,73,74].

### 19.4. Application-Based Scaling Map

The scale-up value of a dopant strategy changes significantly according to the target application. Transparent conducting thin films, selective gas sensors, photocatalytic powders, photoanodes, electrochromic layers, and hole-transport materials do not operate under the same quality regime. In thin-film applications, thickness uniformity, surface roughness, adhesion, crystallographic texture, electrical conductivity, optical transmittance, and low scattering are critical. In powder and composite applications, by contrast, agglomeration control, specific surface area, phase stability, dispersibility, pore accessibility, and long-term suspension or coating stability may become more decisive. Therefore, the comparison between boron- and rare-earth-based dopants should not be made in an application-neutral manner. It should instead be positioned within an application-specific scale-up map. The same dopant family may be optimal for one application while remaining a secondary or high-risk option for another [16,26,27,38,39,57,62,67,80,86,87,88,89,90,91,92,93,94].

The general trend can be summarized as follows. In high-volume, cost-sensitive, and process-tolerant platforms where phase stability, morphological uniformity, and reproducible surface quality are dominant requirements, boron-based dopants appear more scalable and strategically manageable. In contrast, rare-earth dopants may offer selected advantages in applications requiring highly specific functions, such as sensors with exceptional selectivity, luminescent systems, redox-active photocatalysts, or optoelectronic layers requiring precise energy-level tuning. However, even in these cases, the processing window, quality-control burden, raw-material vulnerability, and recovery cost must be included in the evaluation. Therefore, dopant studies that do not provide an application-specific scale-up perspective make an incomplete contribution to the broader discussion of strategic technology development [4,9,13,71,72,73,74,75].

### 19.5. Recommended Evidence Package for Technology Readiness Level

The technology readiness level concept is often used only nominally in advanced oxide dopant studies. Genuine TRL progression requires different types of evidence at different stages of development. At low TRLs, mechanistic evidence, proof of concept, structural validation, and preliminary functional performance are central. At intermediate TRLs, reproducible prototypes, batch consistency, process-window definition, aging tests, environmental stability, and preliminary scale-up trials become necessary. At high TRLs, supply agreements, quality-management systems, standardization protocols, waste-stream analysis, cost models, regulatory compatibility, and life-cycle considerations become increasingly important. The recommendation of this study is that dopant studies should explicitly and honestly state the TRL level supported by the generated data. Presenting laboratory-scale structural or functional results as industry-ready technology is misleading from both scientific and policy perspectives [13,71,72,73,74,75].

Within this framework, boron-based dopants often provide a more reliable pathway during the transition from low to intermediate TRL levels because they tend to offer broader processing tolerance, lower strategic vulnerability, and more manageable structural evolution. Rare-earth dopants, although capable of delivering strong and sometimes indispensable performance gains, may become more challenging at intermediate and high TRL stages due to supply-chain exposure, stricter process-control requirements, phase-segregation risk, and recovery difficulties. Therefore, public funding calls should evaluate not only the novelty of a dopant strategy, but also the scalability pathway through which that novelty can be transformed into a reproducible technology. The central argument of this study is that dopant engineering should be analyzed not only within a crystallographic and defect-chemical framework, but also within the economics, logistics, and governance of technology readiness [4,9,10,11,12,13,14,71,72,73,74,75].

The comparative risk matrix of dopant strategies in terms of technology readiness level and scale-up is summarized in Table 14.

## 20. Application-Specific Comparative Roadmaps: Sensors, Photocatalysis, Photoelectrochemistry, Optoelectronics, and Energy Storage

### 20.1. Dopant Selection for Gas Sensor Applications

The gas-sensor literature is one of the fields in which the performance consequences of dopant strategies are most directly visible, since sensitivity, selectivity, response/recovery time, baseline resistance, and operating temperature are strongly influenced by dopant chemistry, surface defect structure, and grain-boundary behavior. However, a recurring interpretive limitation in this field is that a high response value toward a single target gas may obscure the accompanying structural, processing, and strategic costs. In ZnO- and SnO_2_-based sensors, rare-earth dopants often become prominent because they can enhance surface adsorption centers, regulate oxygen-vacancy concentration, and modify surface reaction kinetics. Ce-based modifications are particularly important in this respect because of their redox activity and oxygen storage/release capability. By contrast, boron-based dopants may provide a more balanced adjustment of baseline conductivity, grain-size control, and surface accessibility, especially when they preserve a fine-grained and homogeneous morphology [26,27,38,43,55,56,57,58,59,66,68,80,86].

In sensor applications, the strategically decisive question is whether the desired increase in sensitivity or selectivity can be achieved without using rare-earth elements or by using dopants associated with lower supply risk. When functional substitution is possible, a boron-based approach constitutes a more rational starting point because gas sensors can evolve into high-volume, cost-sensitive, and batch-dependent products, where raw-material availability, reproducibility, and process tolerance become critical. Rare-earth-doped platforms can be more strongly justified in niche sensors requiring exceptional selectivity, low operating temperature, or specific redox-mediated surface chemistry. This distinction defines the key threshold that connects laboratory-scale sensing performance to product strategy: rare-earth dopants are most defensible when they provide a clearly non-substitutable sensing function, whereas boron-based systems are stronger candidates for scalable and cost-sensitive sensor platforms [4,9,26,27,43,55,56,57,58,59,66,68,80,86].

### 20.2. Photocatalysis and Adsorption–Photocatalysis Hybrid Systems

In photocatalysis, dopant selection is commonly discussed through band-gap modification, charge-carrier separation, reactive oxygen species generation, and surface-active-site density. Rare-earth dopants such as Ce, La, and Y can provide advantages in ZnO- and SnO_2_-based systems by enhancing charge separation, extending visible-light response, regulating oxygen vacancies, or accelerating surface redox cycles. However, many composite or heterophase structures also introduce particle growth, agglomeration, local surface heterogeneity, and secondary-phase formation. Therefore, photocatalytic performance should not be interpreted only through degradation percentage, apparent rate constant, or first-cycle activity. It must also be evaluated together with structural integrity, reusability, phase stability, surface accessibility, and resistance to photocorrosion or deactivation [37,38,39,40,41,42,43,44,57,58,59,60,61,62,65,68].

Boron-based strategies can operate through two distinct but complementary pathways in photocatalytic systems: direct boron doping and h-BN/B_4_C-based interface engineering. In h-BN-supported architectures, charge separation, adsorption behavior, particle dispersion, thermal dissipation, and interfacial stability may change simultaneously. Similarly, B_4_C/ZnO systems may contribute through interfacial charge transfer and composite-level surface activation rather than through conventional lattice doping. This indicates that boron should not be regarded merely as a light-element dopant; it can also function as a tool for heterostructure design and adsorption–photocatalysis coupling. From a public-policy perspective, photocatalytic systems are particularly relevant because of their potential use in water treatment, pollutant degradation, and environmental remediation. For these applications, reuse stability, low-cost supply, and process scalability are at least as important as first-cycle degradation efficiency. Under this broader criterion, boron-based platforms often generate stronger total value, especially when their performance falls within a comparable range to rare-earth-based alternatives [29,37,40,41,42,52,53,54].

### 20.3. Photoelectrochemistry and Solar-Energy-Based Conversion Processes

In photoelectrochemical systems, dopant selection affects not only surface reaction kinetics, but also bulk carrier transport, surface-to-bulk potential gradients, recombination dynamics, and interfacial charge-transfer barriers. Hematite is an instructive example in this regard because its short hole diffusion length, low carrier mobility, and high recombination rate make dopant engineering especially critical. The potential of boron doping to regulate surface states, improve charge separation, and modify local bonding environments has therefore become a notable trend in the hematite literature. Rare-earth dopants may also be beneficial in specific redox architectures or surface-reactive systems; however, the balance between bulk phase stability, dopant segregation, and surface enrichment is delicate. For this reason, in photoelectrochemistry, the dopant should not only be considered as a bulk lattice modifier, but also as part of a surface coating, gradient structure, heterointerface, or interfacial passivation strategy [67,81,82,83,84,85,93].

The strategic implication for this field is clear. If the objective is to develop long-lasting, large-area, and low-cost photoactive electrodes, supply security and process reproducibility are as important as the intrinsic dopant effect. Rare-earth dopants can generate specific functionalities such as enhanced redox cycling, modified band alignment, or improved interfacial reaction kinetics. However, these functions must be evaluated together with electrode lifetime, scale-up feasibility, precursor cost, and phase stability under operating conditions. Boron-based strategies, particularly when combined with surface engineering or interface-controlled architectures, may offer broader scale-up potential and lower strategic vulnerability. Therefore, photoelectrochemical studies should routinely report long-term stability, within-batch variation, post-operation structural changes, and impedance evolution in addition to conventional photocurrent or conversion metrics [4,9,13,67,81,82,83,84,85,93].

### 20.4. Optoelectronic and Transparent Conducting Structures

In optoelectronic applications, dopant selection is centered on carrier concentration, carrier mobility, band alignment, optical transmittance, defect-related emission, and interfacial compatibility with device architectures. ZnO, SnO_2_, and NiO represent complementary platforms in this context. ZnO and SnO_2_ can function as transparent conducting oxides, electron-transport layers, or active semiconducting materials, whereas NiO is widely considered as a p-type oxide and hole-transport layer. Boron doping is particularly important in transparent conducting thin films because of its potential to tune the balance between electrical conductivity and optical transmittance while preserving relatively smooth and homogeneous film morphology. Rare-earth dopants, by contrast, can provide specific advantages in photoluminescence control, UV emission, energy-level engineering, and defect-state modulation [31,36,45,48,49,50,51,64,65,66,76,77,78,79,80]. The central decision problem in optoelectronic systems is the balance between high optical/electronic functionality and high process robustness. For example, Y-doped NiO or Ce-doped ZnO structures may provide superior parameters in certain devices by modifying energy levels, emission behavior, or carrier transport. However, if a comparable performance window can be approached through simpler boron-based thin-film chemistry, the strategically more resilient pathway remains on the boron side. Therefore, optoelectronic publications should not discuss dopant success only through device efficiency, photoluminescence intensity, band-gap shift, or carrier concentration. They should also evaluate large-area uniformity, thickness control, wafer- or substrate-scale reproducibility, environmental stability, processing compatibility, and raw-material sustainability. This type of evaluation is essential for distinguishing laboratory-level optical enhancement from industrially meaningful optoelectronic material development [4,9,16,31,36,48,49,50,51,64,65,79,87,89,94].

### 20.5. Energy Storage and Electrochemical Applications

Energy storage applications constitute another important testing ground for dopant strategies, particularly in composite, porous, and high-surface-area oxide systems. In this context, electrical conductivity, ion diffusion, active surface area, surface redox centers, electrolyte accessibility, mechanical stability, and cycling durability become jointly important. Studies showing that B-doped NiO and boron-containing composite structures can significantly enhance electrochemical capacitance demonstrate that boron-based strategies are not merely secondary or low-impact tools in energy storage. Instead, they can contribute through improved morphology, controlled defect chemistry, enhanced surface accessibility, and interfacial stabilization. Rare-earth modifications may also improve specific electrochemical behaviors, especially when redox activity or defect-mediated transport is required. However, cost, recovery, and supply risk become even more critical in this field because product-volume expansion can rapidly transform raw-material vulnerability into a scale-dependent economic and strategic limitation [16,77,85,86,89,90,91,92,93,94].

From the perspective of energy storage, the general recommendation is to evaluate rare-earth dopants in exceptional and high-value-added functions, while considering boron-based approaches across a broader application base. Particularly when interfacial durability, cycle life, impedance evolution, cost, and end-of-life material recovery are interpreted together, boron-based strategies appear to offer a stronger starting portfolio for both public and industrial stakeholders. The highest-value direction for future research in this field would be standardized experiments that directly compare B- and rare-earth-based dopants within the same electrode architecture, electrolyte system, mass loading range, and cycling protocol. Such studies should report not only capacitance or capacity, but also rate capability, coulombic efficiency, impedance evolution, structural degradation after cycling, and reproducibility across independent electrodes [16,72,73,74,77,85,86,89,90,91,92,93,94].

The comparative decision map for dopant-family selection across major application areas is summarized in Table 15.

## 21. Expanded Research Agenda, Testable Hypotheses, and Standardized Evidence Package

### 21.1. First-Generation Hypotheses: Structural and Morphological Level

This study is designed not only to synthesize previous studies, but also to generate testable hypotheses for future research. The first-generation hypotheses are positioned at the structural and morphological level. Hypothesis H1 can be formulated as follows: at the same atomic dopant level and within the same thermal-treatment window, boron-based dopants will produce lower agglomeration and a narrower grain-size distribution than rare-earth dopants in n-type oxides such as ZnO and SnO_2_. Hypothesis H2 can be formulated as follows: rare-earth dopants may generate higher surface-defect density and more pronounced application-specific performance enhancement within a low-doping window; however, once this window is exceeded, secondary-phase formation, dopant segregation, and morphological heterogeneity will increase more rapidly. These two hypotheses can be tested through direct comparative experiments and may, for the first time, integrate the fragmented observations reported in the literature within a unified experimental framework [31,32,33,34,35,36,47,68,76,77,78,79,80].

The second set of structural hypotheses concerns cross-matrix transferability. Hypothesis H3 can be formulated as follows: the boron-favorable morphological stability observed in ZnO will also be observed in SnO_2_ and, to some extent, in Fe_2_O_3_; however, in NiO, this stability may emerge together with optical or electrical trade-offs depending on the target application. Hypothesis H4 can be formulated as follows: the functional superiority provided by rare-earth dopants will be more pronounced in n-type oxides through surface-redox and oxygen-vacancy-mediated mechanisms, whereas in p-type NiO it will emerge more clearly through energy-level alignment, carrier-density modulation, and hole-transport-related effects. These hypotheses define a research program that is more valuable than isolated article designs, because they develop a transferable mechanistic language regardless of whether the final results confirm or reject the initial assumptions [16,76,77,78,79,80,81,82,83,84,85,86,87,88,89,90,91,92,93,94].

### 21.2. Second-Generation Hypotheses: Policy, Scalability, and Decision Thresholds

Second-generation hypotheses are directly linked to science policy, technology readiness, and strategic materials governance. Hypothesis H5 can be formulated as follows: when the technical performance difference remains below a defined threshold, boron-based dopants will surpass rare-earth dopants in total technological value because of their stronger supply security, broader manufacturability, lower strategic vulnerability, and higher compatibility with domestic value-chain development. Hypothesis H6 can be formulated as follows: for rare-earth dopants to be considered rational within public R&D prioritization, they must demonstrate not only a statistically significant technical advantage, but also a strategically non-substitutable superiority. These hypotheses can be evaluated through quantitative multi-criteria decision-making models, scenario-based analysis, cost sensitivity, supply-risk sensitivity, and technology-readiness assessment. In this way, the materials-focused study establishes a genuine analytical dialogue with public policy rather than treating critical raw materials as a peripheral discussion [4,5,6,7,8,9,10,11,12,13,14,71,72,73,74,75]. An important implication of this approach is the need to redesign research funding mechanisms. If project calls reward only the highest instantaneous performance value, researchers may be directed toward strategically vulnerable systems with high publication potential but limited industrial resilience. By contrast, if funding calls assign explicit scores to manufacturability, process reproducibility, supply security, circularity, and alignment with domestic resource capacity, the research portfolio will naturally evolve toward a more resilient and strategically coherent structure. Therefore, the research agenda emerging from this article is addressed not only to laboratory researchers, but also to institutions responsible for designing funding calls and national technology programs. Unless a bidirectional interaction is established between science policy and materials chemistry, the critical raw material discussion risks remaining only a “final section” appended to a conventional materials manuscript [10,11,12,13,14,71,72,73,74,75].

### 21.3. Advanced Characterization and Validation Roadmap

For future studies, the highest-value methodological step is to construct the characterization package in a more integrated and hypothesis-driven manner. Although XRD, FE-SEM, EDX, XPS, Raman spectroscopy, and FT-IR spectroscopy constitute the core characterization framework of this study, additional techniques such as Rietveld refinement, depth-profiled XPS, high-resolution TEM/SAED, BET surface-area analysis, and electrical or electrochemical impedance spectroscopy should be incorporated where possible. However, the sequence and logical function of the evidence are as important as the number of techniques used. The aim is not to maximize instrument use, but to transform the proposed dopant mechanism into a falsifiable model. For example, the claim that “boron is incorporated into the lattice” should be tested not only by detecting a B-related XPS signal, but also by evaluating whether XRD, Raman, FT-IR, and morphological observations consistently support lattice-level incorporation rather than surface complexation or grain-boundary enrichment [13,25,30,76,77].

Similarly, the claim that “rare-earth doping increased oxygen vacancies” should not rely solely on O 1s deconvolution. It should also be supported by surface-redox behavior, application testing, possible secondary-phase signatures, dopant chemical-state analysis, and, where possible, additional evidence sensitive to valence-state variation. The value of advanced characterization lies not merely in generating complex datasets, but in systematically eliminating alternative explanations. A methodologically strong study is not the one that produces the largest amount of data, but the one that establishes the strongest falsification framework. Therefore, the research agenda proposed here emphasizes not only the scope of the evidence package, but also the logical sequencing and cross-validation of evidence [13,30,31,76,77,78,79,80,93].

### 21.4. Data Integration, Open Science, and Comparability

Another factor that would substantially increase the impact of future studies comparing boron- and rare-earth-based dopants is the reporting of data in machine-readable, standardized, and comparable formats. In the current literature, dopant levels are reported inconsistently as wt.%, at.%, mol.%, or precursor molar ratios; annealing duration, heating rate, and atmosphere are not always clearly specified; and morphological quantification is often absent. This fragmented reporting practice weakens the reliability of critical studies and makes quantitative meta-analysis difficult. Standardized data templates would allow results obtained by different laboratories to be compared meaningfully within the same matrix and across different oxide systems [13,15,23].

The open science approach is therefore not merely an ethical preference, but a strategic necessity. In materials research linked to critical raw material policy, sharing raw characterization data, XRD fitting files, XPS deconvolution parameters, representative SEM image sets, image-analysis codes, and negative or non-optimized results is important for both scientific transparency and the effective use of public resources. High-visibility results that cannot be reproduced may direct the research system toward misleading incentives. In contrast, standardized and curated datasets can more rapidly reveal the processing windows in which boron- and rare-earth-based dopant strategies are genuinely superior, conditionally equivalent, or strategically unjustified [13,71,72,73,74,75].

### 21.5. Programmatic Research Sequence: From a Single Article to a Research Platform

The research sequence derived from this study can be structured in three stages. The first stage should involve a fully controlled comparative series on a single matrix, for example by testing B, B_4_C, h-BN, CeO_2_, La_2_O_3_, and Y_2_O_3_ modifications in ZnO under identical precursor chemistry, loading logic, and thermal-treatment conditions. The second stage should transfer the most effective and structurally stable dopant families identified in the first stage to SnO_2_, Fe_2_O_3_, and NiO systems. The third stage should involve application-specific selective deepening through sensor, photocatalytic, photoelectrochemical, optoelectronic, or electrochemical validation. Such a sequence would move beyond the production of a single publication and establish a comparative materials-governance platform [16,76,77,78,79,80,81,82,83,84,85,86,87,88,89,90,91,92,93,94].

The main value of this programmatic sequence is that it can generate complementary articles proceeding along the same analytical backbone. The first publication may address the structural and morphological foundation of boron- and rare-earth-based dopant strategies. The second may examine the mechanism, transferability, and matrix-specific limits. The third may focus on application performance, scale-up potential, supply-risk thresholds, and public R&D prioritization. In this way, each publication can preserve its own originality, while the overall research pathway can evolve into a national strategic materials platform. This design moves the topic beyond a narrow discussion of “dopant performance” and transforms it into a broader research axis that systematically connects laboratory-scale materials data with resource policy, technology readiness, and public prioritization [4,9,10,11,12,13,14,71,72,73,74,75].

The testable hypotheses, validation methods, and expected strategic outcomes for future research are summarized in Table 16.

## 22. Common Interpretive Errors in the Literature and Methodological Warning Signs

### 22.1. Conflating Doping, Composite Formation, and Surface Modification

The most common conceptual error in the dopant literature is the tendency to group intralattice doping, composite formation, surface enrichment, and interfacial modification under a single umbrella and to interpret them using the same mechanistic language. However, a system in which B atoms are genuinely incorporated into the host lattice is not equivalent to an h-BN-supported ZnO heterointerface. Similarly, a sample in which Ce is assumed to be homogeneously dissolved in the ZnO lattice cannot be evaluated in the same category as a nanocomposite containing CeO_2_ clusters or surface-segregated Ce-rich domains. When this distinction is not made, many effects described in the literature as “dopant effects” are, in fact, consequences of interface engineering, surface modification, or secondary-phase formation. Such ambiguity directly weakens the mechanistic validity of the conclusions [29,30,38,39,40,41,42,58,76,80].

Therefore, future publications should clearly define the nature of the adopted modification strategy from the outset: substitutional lattice doping, interstitial doping, surface enrichment, grain-boundary segregation, heterostructure formation, or composite engineering. This distinction is not a minor terminological issue. It determines the appropriate characterization methods, the validity of the performance interpretation, the expected structural signatures, and the transferability of the results to other oxide matrices. Academic rigor in this context requires a clear conceptual vocabulary and a coherent evidence architecture that prevents structurally distinct mechanisms from being evaluated as if they were identical [29,30,77,80].

### 22.2. Treating Weight Percentage and Atomic Percentage as Equivalent

The second major error is the intuitive but methodologically incorrect equivalence often established between wt.% and at.%. When a light element such as boron and heavier elements such as La, Ce, or Y are introduced at the same weight percentage, the actual number of dopant atoms, the cation ratio, and the chemical loading can differ markedly. Nevertheless, the literature may present statements such as “3 wt.% B-doped ZnO and 3 wt.% La-doped ZnO were compared,” and then interpret the two systems as if they contained equivalent dopant levels. Such comparisons can lead to misleading conclusions, particularly in discussions of defect compensation, lattice strain, oxygen-vacancy formation, and grain-boundary segregation. A methodologically robust comparative study should therefore be designed, wherever possible, on the basis of chemical equivalence rather than nominal mass equivalence [13,33,34,44,45,46,47,48,49,50,51,60,61,62,63,64,65].

This issue is not merely a technical detail; it can undermine the fundamental logic of comparative interpretation. If the actual number of dopant atoms introduced into the system differs substantially, it becomes unclear whether the observed structural or functional difference originates from the dopant family itself or from unequal chemical loading. Therefore, the standard proposed here is to retain wt.% information for practical reproducibility, while establishing the primary comparison on the basis of at.%, mol.%, or cation-to-metal ratio. Similarly, introducing different oxide precursors such as CeO_2_, La_2_O_3_, and Y_2_O_3_ at the same nominal percentage does not by itself imply chemical equivalence. The oxide form, valence state, molecular weight, active cation fraction, and actual number of incorporated or surface-accessible ions must also be considered [13,31,77].

### 22.3. Selected Image and Peak Performance Bias

The third methodological warning sign is selected-image and peak-performance bias. In FE-SEM or TEM-based studies, it is common to select the most regular, homogeneous, or visually appealing region and present it as representative of the entire sample. Similarly, reporting only the best-performing sample or the most successful replicate may conceal the true distribution of the material response. This type of selectivity creates a particularly serious problem in rare-earth-modified systems, where batch-to-batch variability may be high due to narrow processing windows, phase-segregation sensitivity, and strong dependence on thermal history. However, boron-based systems are not immune to this issue; abrupt deterioration may also occur outside the optimal low- or intermediate-dopant range. Therefore, peak values should not form the sole basis of interpretation. Distribution, reproducibility, and failure thresholds should be prioritized [13,19,20,24,25].

From a methodological perspective, the most convincing study is not the one that reports the highest individual value, but the one that transparently presents the distribution of structural and functional outcomes. Multiple synthesis replicates at the same dopant level, multi-area imaging, quantitative image analysis, statistical dispersion values, and error bars should be treated as quality criteria rather than optional additions. If one dopant type produces peak performance within a narrow and poorly reproducible window, whereas another provides slightly lower but more stable performance across a broader processing range, the latter may be more valuable in strategic technology assessment. Publications that fail to make this distinction visible risk confusing laboratory excitement with scientific robustness [4,9,13,71,72,73,74,75].

### 22.4. Non-Normalized Performance Comparisons

The fourth error is the comparison of performance data without adequate normalization. In photocatalysis, different light sources, light intensities, catalyst loadings, solution pH values, irradiation distances, initial pollutant concentrations, or adsorption-equilibration times may be used. In gas-sensor studies, different operating temperatures, target gas concentrations, gas-flow regimes, humidity levels, baseline resistances, and electrode geometries may be involved. Despite these differences, results are often presented as if they directly reflect the intrinsic superiority of the dopant type. This is particularly misleading in boron- and rare-earth-modified systems because these dopant families generate advantages through different mechanisms, and their apparent performance can strongly depend on the experimental window. Unless performance metrics are normalized or at least reported with sufficient contextual detail, the structural and morphological interpretation loses reliability [26,27,38,57,62,66,67,68,80,86,93].

Therefore, a minimum normalization set should be adopted in application-oriented studies. In gas-sensor studies, target gas concentration, operating temperature, relative humidity, carrier-gas flow rate, baseline resistance, response/recovery definition, and repeatability conditions should be reported. In photocatalysis, the light-source spectrum, light flux or irradiance, catalyst loading, solution volume, pollutant concentration, pH, dark adsorption duration, kinetic model, and reusability protocol should be specified. In photoelectrochemical studies, electrolyte composition, light intensity, illumination area, scan rate, reference electrode, stability duration, and post-test structural analysis should be reported in a standardized manner. Only under such conditions can a reliable link be established between the dopant family and the application output. Otherwise, high performance values function more as showcase data than as evidence capable of supporting structural, mechanistic, or strategic conclusions [13,26,27,38,57,62,80,93].

### 22.5. Overextension of Policy Implications

The fifth and perhaps least discussed error is the derivation of overly broad policy implications from limited experimental evidence. It is not scientifically defensible to claim, on the basis of a single host matrix, a single application, or a narrow dopant-loading series, that a country should focus exclusively on one dopant family. Even in the policy-oriented sections of this study, the guiding principle is to formulate recommendations in a context-sensitive manner and in proportion to the strength of the evidence. The domestic resource advantage of boron is a strong strategic reality, but it does not justify the conclusion that rare-earth-based research is unnecessary. Similarly, the superiority of rare-earth dopants in certain applications does not justify extending them indiscriminately across the entire public research portfolio [10,11,12,13,14,71,72,73,74,75].

Therefore, policy recommendations should be formulated not as definitive prescriptions, but as conditional strategic inferences. Within this framework, boron-based dopants should be interpreted as the default priority in high-volume, scalable, and strategically sensitive platforms, provided that they meet the required technical-performance threshold. Rare-earth dopants, by contrast, should be selectively supported when they demonstrate non-substitutable functionality, reproducible superiority, and clear value in applications where boron-based or other accessible alternatives cannot provide an equivalent response. This formulation respects the uncertainties inherent in materials science while still providing policymakers with an actionable decision logic. Scientific responsibility lies precisely in maintaining this balance between technical evidence and strategic inference [10,11,12,13,14,71,72,73,74,75].

The methodological warning signs frequently observed in comparative dopant studies are summarized in Table 17.

## 23. Expanded Characterization Synthesis with Primary Experimental Studies

### 23.1. Structure of the Primary Study Set and Level of Comparability

The seven primary studies incorporated into the analysis constitute a validation-oriented study set that strengthens the characterization framework of this study: ZnO–TiC [16], ZnO–B_4_C [17], ZnO–h-BN [18], ZnO–CNT/TiB_2_ [19], NiO–Y_2_O_3_ [20], NiO–V_2_O_5_ [21], and NiO–TiN [22]. These studies were not selected as random or methodologically unrelated examples. Rather, most of them were developed using a sol–gel or sol–gel-assisted precipitation rationale and share a broadly comparable research architecture based on XRD for structural analysis, FE-SEM/EDX for morphology and elemental distribution, FT-IR for bonding and functional group evaluation, Raman and/or XPS in selected cases for defect and chemical-state analysis, and impedance-based dielectric measurements for functional validation. In this respect, the primary study set provides not only experimental results, but also a shared analytical and measurement language.

Nevertheless, the study set should not be interpreted as fully symmetrical. The denser experimental evidence for boron-based modification is mainly concentrated in the ZnO matrix, whereas the most detailed rare-earth-related characterization is represented by the NiO–Y_2_O_3_ pathway [16,88,89,90]. Therefore, direct numerical comparison cannot be treated as perfectly equivalent in terms of the same matrix, same dopant ratio, same frequency window, and same compositional scale. The shared value of the set is methodological rather than metrological: it enables a disciplined comparison of phase response, morphology, interface formation, and low-frequency polarization using a similar characterization logic.

Accordingly, the expanded synthesis below evaluates the selected primary studies across two complementary analytical planes. The first is the direct characterization plane, in which XRD, FE-SEM/EDX, FT-IR, Raman, and XPS evidence is interpreted in relation to phase formation, lattice behavior, crystallite/grain structure, elemental distribution, and surface chemistry. The second is the functional response plane, in which the observed structural and chemical differences are examined in terms of low-frequency dielectric constant, dielectric loss, and AC conductivity. The evidence chain established through this dual-plane approach provides a stronger analytical framework by linking characterization findings with functional outcomes through a more explicit cause-and-effect logic.

The methodological backbone of the primary study set and its comparative function within the study are summarized in Table 18.

The synthesis and testing conditions used in the primary study set are summarized in Table 19. This table clarifies the level of equivalence: the studies share a broadly related powder-synthesis and characterization philosophy, but they are not used as a fully normalized cross-matrix dataset.

Table 19 is not presented as proof of strict synthetic identity among the seven primary pathways. Instead, it functions as a synthesis/testing-anchor table that makes the limits of equivalence explicit. The compared studies differ in host matrix, additive geometry, loading descriptor, thermal history, and impedance-frequency window; therefore, exact head-to-head normalization is not claimed. Parameters such as precursor molarity, pH, annealing temperature, dwell time, atmosphere, drying conditions, pellet geometry, electrode configuration, and impedance-acquisition protocol are decisive for strict reproducibility, but they are not uniformly harmonized across ZnO–TiC, ZnO–B_4_C, ZnO–h-BN, ZnO–CNT/TiB_2_, NiO–Y_2_O_3_, NiO–V_2_O_5_, and NiO–TiN. For this reason, the primary study set is used only for mechanism-level triangulation of phase response, interfacial morphology, defect chemistry, and low-frequency polarization behavior, rather than for a direct numerical ranking of intrinsic material superiority.

### 23.2. Phase, Lattice Parameter, and Crystallite-Size Trends Based on XRD

XRD evidence from the primary study set demonstrates that the crystallographic imprint of dopant strategies is governed not only by chemical family, but also by the specific structural role assumed by the additive within the host matrix and at its interfaces. In the ZnO–B_4_C system, the ZnO (101) reflection shifts from 36.382° to 36.265°, while the lattice parameters *a* and *c*, as well as the unit-cell volume, increase from 3.225 to 3.235 Å, from 5.266 to 5.282 Å, and from 47.435 to 47.880 Å^3^, respectively. In the same series, the crystallite size increases from 18.953 to 28.865 nm. This combined lattice expansion and crystallite-growth trend suggests that B_4_C does not behave merely as an inert surface additive, but rather as an active secondary phase that modifies ZnO crystal growth and the local strain field around the lattice [17]. A similar tendency is observed in the ZnO–TiC study, where *a* increases from 3.225 to 3.238 Å, *c* from 5.266 to 5.289 Å, and the unit-cell volume from 47.435 to 48.049 Å^3^, while the crystallite size increases from 18.953 to 24.475 nm [16]. In both systems, lattice expansion and enlarged crystalline domains occur simultaneously, indicating that carbide-based additives can influence both crystallographic relaxation and growth kinetics.

The ZnO–h-BN pathway represents a distinct crystallographic response within the broader boron-containing family. The characteristic (002) reflection of h-BN appears at 26.754° and shifts toward the 26.804–26.890° range with increasing additive content. In contrast, the average ZnO cell parameters remain almost unchanged, and the unit-cell volume decreases only slightly from 47.435 to 47.409 Å^3^. More importantly, the average crystallite size decreases from 14.720 to 11.620 nm [18]. This pattern indicates that h-BN does not expand the ZnO lattice in the same manner as B_4_C. Instead, it behaves as a two-dimensional barrier additive that restricts crystal growth, increases the density of nanoscale heterointerfaces, and modifies crystallization through sheet-mediated spatial confinement. Therefore, there is no single XRD signature that can be generalized under the broad label of “boron doping”; different boron-containing architectures may generate fundamentally different crystallographic responses.

On the rare-earth side, the NiO–Y_2_O_3_ study reveals a crystallographic mechanism that differs markedly from the ZnO–B_4_C and ZnO–TiC pathways. Although the cubic NiO structure is preserved, the diffraction peaks shift toward higher 2θ values, and the lattice parameter decreases from 4.194 to 4.164 Å, indicating lattice contraction rather than expansion [20]. Williamson–Hall analysis further shows that microstrain increases, reaching 5.71 × 10^−3^ particularly around 5 wt.% Y_2_O_3_. Rietveld refinement indicates that the material should not be interpreted as a single-phase solid solution, but rather as a two-phase nanocomposite consisting of a strained NiO phase and a finely distributed Y_2_O_3_ secondary phase [20]. This evidence is important because it demonstrates that the lattice response in rare-earth-modified systems cannot be reduced to a simplistic assumption such as “larger ion produces larger lattice.” Defect compensation, interfacial strain, secondary-phase formation, and local stress fields can reverse the direction of the crystallographic response.

When these pathways are considered together, the conclusion is clear: the decisive variable is not only the chemical identity of the additive, but also the crystallographic role it plays within the matrix. B_4_C and TiC mainly operate through lattice expansion and enlarged crystalline domains; h-BN functions through growth suppression, sheet-mediated confinement, and interface multiplication; and Y_2_O_3_ acts through lattice contraction, compressive microstrain, and two-phase interfacial strain. Therefore, dopant families should be reclassified not only according to their chemical identity or position in the periodic table, but also according to the type of crystallographic response they induce.

### 23.3. Morphological and Interfacial Organization Based on FE-SEM/EDX

The morphological evidence provides the spatial counterpart of the XRD findings. In the ZnO–B_4_C study, pristine ZnO exhibited agglomerated, nearly spherical particles, whereas B_4_C displayed a plate-like morphology. In the composite samples, ZnO particles were observed to cluster, anchor, and grow on these plate-like surfaces [17]. A similar interface-mediated organization is observed in the ZnO–h-BN study, where FE-SEM images revealed a distinct platelet or nanoplate h-BN architecture, with ZnO particles forming on the surfaces of these plates. As the additive content increased, the presence of the plate-like phase and the extent of surface coverage became progressively more dominant [17]. The ZnO–TiC system likewise generated an interfacial architecture in which ZnO grew on TiC surfaces [16]. Taken together, these three observations suggest that the common feature of boron- and carbide-based additives is not merely chemical modification, but the creation of new interfacial planes that can act as charge-accumulation, nucleation, and polarization sites.

The EDX data are also consistent with this spatial interpretation. In the ZnO–B_4_C series, pristine ZnO contained 87.32 wt.% Zn and 12.68 wt.% O, whereas the Zn/O/C/B distribution evolved to 28.28/48.56/4.77/18.39 wt.% in the 30 wt.% B_4_C-containing sample [17]. In the h-BN series, local EDX measurements showed that B and N increased to approximately 25.13 and 15.40 wt.%, respectively, in the 10 wt.% composite [18]. Although such values should be interpreted with caution because EDX has limited quantitative reliability for low-atomic-number elements, they nevertheless confirm that the additive is not merely nominally present, but exists as an interfacial component within the composite architecture. At the same time, the difficulty of quantitatively tracking boron by EDX reinforces the methodological need to cross-validate boron-containing systems using FT-IR, Raman, XPS, and XRD evidence.

In the NiO–Y_2_O_3_ pathway, the morphological response is substantially different. Instead of sheet-based interface multiplication, grain coalescence and grain-boundary reorganization become dominant. FE-SEM-based grain-size evaluation shows an increase from approximately 70–90 nm to 180–220 nm, while EDX mapping indicates that Y is distributed within the NiO matrix without macroscopic phase segregation [20]. Therefore, unlike the h-BN/B_4_C-based ZnO systems, where the interface is constructed through plate-like or sheet-like additive surfaces, Y_2_O_3_ acts as an interface-engineering agent that reshapes grain-boundary chemistry and intergranular topology within NiO.

The dielectric implication of this morphological distinction is significant. Sheet-based boron-containing additives generate high interfacial area, geometrically anisotropic charge-accumulation planes, and surface-mediated polarization sites. By contrast, the Y_2_O_3_/NiO system produces denser grains, thicker boundary regions, and strain-rich heterointerfaces. Both pathways may contribute to Maxwell–Wagner–Sillars polarization; however, their morphological causalities differ. In one case, geometric anisotropy and interface multiplication dominate, whereas in the other, boundary resistance, defect enrichment, and chemically modified intergranular regions become more prominent. Therefore, the same functional outcome, namely dielectric enhancement, may originate from different structural and interfacial mechanisms.

It should also be emphasized that the primary morphological comparison is not converted into a universal agglomeration index or statistically normalized particle-size distribution because the SEM datasets were not generated under an identical image-acquisition and image-analysis protocol. Morphology-related inference is therefore limited to reported grain-size ranges, observable interfacial topology, additive distribution, and EDX-supported spatial trends. Quantitative morphology is reported only where the original primary data provide compatible metrics, such as the approximate grain-size evolution in the NiO–Y_2_O_3_ pathway. A fully normalized morphological ranking would require identical magnification windows, multi-area image sampling, automated segmentation criteria, particle-count statistics, independent synthesis replicates, and replicate-level error analysis for all seven systems.

### 23.4. Integrated Interpretation of FT-IR, Raman, and XPS: Revealing Defect and Interface Chemistry

The spectroscopic triad of FT-IR, Raman, and XPS constitutes the strongest common denominator of the primary study set. In the ZnO–B_4_C system, the B–B vibration around 1072 cm^−1^ and the linear C–B chain signature around 1500 cm^−1^, interpreted together with the ZnO-related bands at 570 and 645 cm^−1^, indicate that boron carbide is preserved within the composite and modifies the bonding environment around ZnO [17]. In the ZnO–TiC study, the Ti–C bond appears around 501 cm^−1^, while the ZnO bands are preserved in the 570–645 cm^−1^ range. This confirms that TiC behaves as a chemically active interfacial phase in contact with ZnO, while not fully disrupting the main oxide framework [16]. In the ZnO–h-BN pathway, the coexistence of B–N vibrations around 786 and 1342 cm^−1^ with Zn–O bands in the 383–582 cm^−1^ region strongly supports the surface-based composite character of the h-BN/ZnO architecture [18].

On the rare-earth side, the NiO–Y_2_O_3_ study provides a more detailed chemical-state interpretation. XPS data show that Y^3+^ species establish chemical contact with the NiO matrix, while the Ni 2p region largely preserves NiO-like chemistry and defect-related O 1s components increase [20]. The increase in the surface Y/(Y + Ni) ratio from 0.0718 to 0.3951 demonstrates that Y is not merely a phase added to the bulk, but a chemically active species operating at the interface and in near-surface regions [20]. The phonon softening and band broadening reported by Raman and FT-IR provide the vibrational and mechanical counterpart of this chemical change, indicating that bond force constants and local symmetry distributions are reorganized by Y_2_O_3_ incorporation.

The NiO–V_2_O_5_ control study further strengthens the generalizability of this interpretation. The emergence of V=O and V–O–V bands, the preservation of NiO-like Ni 2p chemistry, and the increase in defect-related O 1s components show that dielectric enhancement cannot be attributed solely to the presence of a rare-earth element. Instead, defect chemistry and interface chemistry constitute a decisive axis in their own right [21]. The NiO–TiN pathway further demonstrates that a conductive nitride phase, together with TiO_x_/TiO_x_N_y_-like sublayers formed near the surface, can transform the interfacial electronic structure and the associated polarization mechanisms [22]. Therefore, in dopant and composite studies where FT-IR, Raman, and XPS are not interpreted together, there is a high risk that the actual defect and interface chemistry will remain insufficiently represented.

In this context, the most reliable inference chain is as follows: lattice response is identified by XRD; interface geometry and elemental distribution are observed by FE-SEM/EDX; bonding state and defect chemistry are verified by FT-IR, Raman, and XPS; and only after this sequence are dielectric data interpreted mechanistically. The selected primary studies become scientifically more persuasive to the extent that they employ this complete evidence chain rather than relying on isolated characterization outputs.

### 23.5. Dielectric and AC Conduction Meta-Synthesis at the Low-Frequency Anchor

Because the frequency ranges used in the selected primary studies are not fully equivalent, the most reliable comparative strategy is to explicitly define the reported low-frequency anchor. Accordingly, the synthesis below uses 1 kHz wherever possible and the nearest reported low-frequency value when 1 kHz is not available, such as 10 kHz for the ZnO–B_4_C system. This comparison is not intended to produce a direct “winner ranking” among the materials. Rather, it aims to clarify which system generates which type of dielectric gain, how this gain is structurally supported, and whether it is accompanied by a conductivity penalty or a more favorable AC conduction response.

Within this framework, the ZnO–B_4_C, ZnO–h-BN, and ZnO–TiC pathways can be interpreted primarily through interface-assisted polarization and morphology-mediated charge accumulation. The dielectric response is enhanced not simply because a secondary phase is added, but because the secondary phase modifies the spatial distribution of interfaces, local charge-trapping regions, and grain-boundary-related polarization centers. In the NiO–Y_2_O_3_ pathway, by contrast, dielectric enhancement is more strongly associated with defect-enriched grain boundaries, interfacial strain, and chemically modified near-surface regions. The NiO–V_2_O_5_ and NiO–TiN systems confirm that this response can also be generated through non-rare-earth pathways when the additive produces sufficient defect modulation, conductive interfacial sublayers, or redox-active bonding environments [20,21,22].

The broader implication is that dielectric enhancement should not be interpreted solely as a direct function of dopant identity. It is more accurately understood as the combined result of crystallographic response, interfacial geometry, defect chemistry, and low-frequency polarization mechanisms. Systems with sheet-like additives may enhance dielectric behavior by increasing interfacial area and charge-accumulation sites, whereas systems with oxide or nitride secondary phases may enhance dielectric behavior by modifying boundary resistance, local defect density, or electronic heterogeneity. Therefore, the low-frequency dielectric anchor provides a useful common comparison point, but its interpretation must remain structurally and chemically grounded.

**Figure 4:**
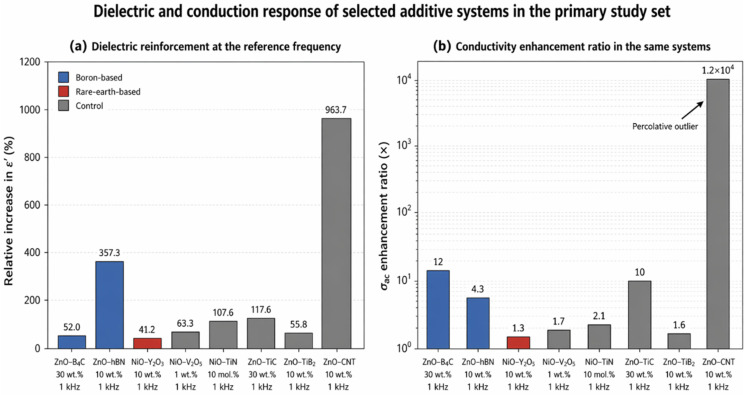
Dielectric enhancement and AC-conduction response of selected additive systems in the primary study set at the stated reference frequency. The values are extracted from refs. [16,17,18,19,20,21,22] and should be interpreted together with the matrix, frequency anchor, loading basis, and comparability limits reported in Table 20; the figure is not intended as a fully normalized cross-matrix ranking.

The dielectric and conduction responses at the low-frequency anchor derived from the primary study set are quantitatively compared in Figure 4 [16,17,18,19,20,21,22].

Three major behavioral clusters can be distinguished from the comparative assessment presented in Table 20. The first cluster comprises systems that exhibit controlled dielectric enhancement with a relatively limited leakage tendency; NiO–Y_2_O_3_ and ZnO–TiB_2_ are positioned closest to this category [19,20]. The second cluster includes systems that generate pronounced interfacial polarization enhancement while simultaneously increasing the dielectric loss component; ZnO–h-BN and ZnO–TiC can be evaluated within this group [16,17,18]. The third cluster corresponds to systems approaching a percolative conduction network. ZnO–CNT represents the clearest example of this behavior and should therefore be interpreted from the perspective of conductive or semiconductive network formation rather than through a conventional dielectric-material comparison [19]. ZnO–B_4_C occupies a transitional position among these three clusters: it does not exhibit as abrupt a polarization–loss trade-off as ZnO–h-BN, but it is also not as conservative as the NiO–Y_2_O_3_ pathway. Nevertheless, its absolute increase in dielectric loss remains substantially more limited than that observed for the h-BN-containing system [17,18,19,20].

## 24. Reinterpretation of Boron and Rare-Earth Dopants Based on the Primary Study Set

### 24.1. Internal Heterogeneity of the Boron Side: B_4_C and h-BN Are Not in the Same Category

Perhaps the most important lesson obtained from the primary study set is that boron-based dopants and additives cannot be treated as a single homogeneous behavioral class. In the ZnO–B_4_C study, the shift of the ZnO (101) reflection from 36.382° to 36.265°, the increase in the a and c lattice parameters from 3.225 to 3.235 Å and from 5.266 to 5.282 Å, respectively, and the increase in crystallite size from 18.953 to 28.865 nm suggest that B_4_C produces an expansive secondary-phase and interfacial effect that promotes crystal growth within the ZnO-based structure [17]. By contrast, in the ZnO–h-BN study, although the h-BN (002) reflection is clearly observed, the unit-cell volume of ZnO remains almost unchanged and even decreases slightly, while the average crystallite size decreases from 14.720 to 11.620 nm [18]. Thus, even within the boron-containing family, two markedly different crystallographic behaviors are observed: at one end, a rigid carbide-based interface that supports crystal growth, and at the other, a two-dimensional interfacial barrier that restricts crystallite development.

This distinction is also directly reflected in the morphological topology. In B_4_C-modified ZnO, spherical and agglomerated ZnO clusters are observed on plate-like B_4_C particles, whereas in h-BN-modified ZnO, a more widespread ZnO distribution is formed over platelet-type structures [17,18]. Therefore, two additive types that may be grouped together under the broader policy advantage of boron-based domestic resources actually generate two different microstructural strategies. B_4_C provides a more mechanically rigid and balanced composite-interface architecture, whereas h-BN constructs a stronger interfacial polarization domain through its high specific interfacial area and oriented sheet geometry. For this reason, any policy-level recommendation in favor of boron-based materials cannot be defended without technical subclassification. The boron family should be interpreted not as a single dopant category, but as a differentiated platform containing distinct lattice, composite, and interface-engineering mechanisms.

### 24.2. Specific Mechanism Revealed by the Y_2_O_3_/NiO Pathway

On the rare-earth side, the highest-resolution primary data series is represented by the NiO–Y_2_O_3_ pathway. In this system, the cubic structure of NiO is preserved, while the lattice parameter decreases from 4.194 to 4.164 Å, and pronounced compressive microstrain accumulates particularly in the 3–5 wt.% Y_2_O_3_ range [20]. When XPS, Raman spectroscopy, and Rietveld refinement are interpreted together, Y_2_O_3_ addition cannot be understood merely as the physical presence of a secondary phase. Instead, it should be interpreted as an active interfacial and defect-modifying component that reorganizes the NiO lattice, near-surface chemistry, and grain-boundary environment.

This mechanism differs from the strong sheet-mediated interfacial enhancement observed on the boron side, which may lead to higher dielectric response but also to increased dielectric loss. In the NiO–Y_2_O_3_ series, the dielectric constant at 1 kHz increases from 6.607 to 9.327, whereas AC conductivity increases only from 1.602 × 10^−8^ to 2.027 × 10^−8^ S/cm [20]. The absolute increase in permittivity is not as striking as that observed in h-BN/ZnO systems; however, the limited leakage tendency and more controlled dielectric-loss behavior make this pathway particularly valuable for functional ceramic layers requiring a low-loss operating window. Therefore, the value of rare-earth-based dopants should not be evaluated only by their ability to maximize ε′. It should also be assessed according to their capacity to generate a controlled defect/interface window without excessive leakage, phase instability, or loss amplification.

### 24.3. Necessity of Control Systems: Interpretation Through TiC, V_2_O_5_, TiN, CNT, and TiB_2_

The TiC/ZnO, V_2_O_5_/NiO, TiN/NiO, and CNT/TiB_2_/ZnO studies serve as necessary control systems for interpreting the boron versus rare-earth distinction. In the ZnO–TiC pathway, the ZnO lattice expands, the crystallite size increases from 18.953 to 24.475 nm, and ε′ rises from 12.07 to 26.26 at 1 kHz [16]. In the NiO–V_2_O_5_ pathway, ε′ increases from 8.04 to 13.12 and σac from 3.30 × 10^−8^ to 5.71 × 10^−8^ S/cm, accompanied by V=O/V–O–V signatures and an increase in defect-related O 1s components [21]. The NiO–TiN system further demonstrates that a conductive nitride phase can increase ε′ from 7.155 to 14.854 through interfacial resistance contrast and microcapacitor-type network formation, clearly showing that dielectric response is governed not only by chemical doping, but also by electrical contrast and interfacial architecture [22]. The most extreme example is the ZnO–CNT system. As CNT content increases, ε′ at 1 kHz rises from 19.260 to 204.885, while σac increases sharply from 1.07 × 10^−8^ to 1.32 × 10^−4^ S/cm [19]. This evidence demonstrates that high permittivity does not always correspond to the desired type of dielectric enhancement. In some cases, it emerges together with the formation of a percolative conductive network, where the increase in polarization is accompanied by a substantial conductivity penalty. Therefore, a fair comparison of boron-based and rare-earth-based strategies cannot be made without first identifying the mechanism generated by the additive. Intralattice defect engineering, sheet-based interface multiplication, boundary-resistance modulation, conductive contrast, and percolation-driven polarization must be methodologically distinguished from one another.

## 25. Reevaluation of the Main Thesis in Light of Primary Studies

The primary study set enables the central thesis of this article to be reevaluated in a sharper and more defensible manner. First, “boron doping” should not be treated as a single and homogeneous performance family. Substantial differences exist between B_4_C/ZnO and h-BN/ZnO in terms of crystallographic response, morphology, interface geometry, and dielectric behavior. B_4_C/ZnO represents a more growth-supporting and mechanically rigid composite-interface route, whereas h-BN/ZnO represents a sheet-mediated interface-multiplication route with stronger interfacial polarization but a higher risk of loss increase. Second, “rare-earth doping” does not necessarily have to produce the highest absolute permittivity to be valuable. The Y_2_O_3_/NiO pathway offers a more limited but more controlled dielectric enhancement pattern, pointing to a low-leakage and well-regulated interface-engineering regime. Third, control systems such as V_2_O_5_/NiO and TiN/NiO clearly demonstrate that high dielectric response arises not only from dopant chemistry, but also from microstructural variables such as defect density, interfacial resistance contrast, conductive sublayers, and proximity to the percolation threshold [20,21,22]. Therefore, public R&D prioritization should not be built on a binary and reductionist opposition between boron-based and rare-earth-based dopant families. Instead, it should be based on identifying which dopant class provides the strongest scientific and strategic combination for a given host matrix, target application, interface geometry, and acceptable loss window. In light of the primary study set, boron-based platforms such as B_4_C and h-BN should be regarded as first-line candidates in broad application programs sensitive to supply risk, scalability, and domestic value-chain development. By contrast, Y_2_O_3_-based rare-earth strategies should be interpreted as conditionally prioritized options in niche applications requiring more controlled defect/interface tuning, low leakage, and disciplined functional-layer design [17,18,19,20].

When these findings are evaluated together, the study presents a multilayered critical synthesis that deepens the conceptual study framework through primary characterization evidence. The distinguishing contribution is not merely to state which dopant produces the higher numerical output, but to show through which structural, chemical, interfacial, and strategic chain that output becomes meaningful. In this way, boron and rare-earth dopants can be interpreted not only as material families, but also as distinct governance regimes in terms of defect architecture, interface design, process tolerance, and supply-policy alignment.

## 26. Limitations

Since a substantial part of the primary study set originates from a similar research pathway and related synthesis tradition, two opposing implications should be considered. On the one hand, the measurement language and characterization framework are relatively consistent, which strengthens the internal coherence of the mechanistic comparison. On the other hand, the limited interlaboratory diversity means that some findings should be further tested through multicenter reproducibility studies. In other words, the selected primary study set provides a validation layer with high internal consistency, but this layer remains open to expansion in terms of external plurality, independent replication, and broader methodological diversity.

A further limitation is that the transferability discussion is not supported by a single unified experimental series across ZnO, SnO_2_, Fe_2_O_3_, and NiO. The cross-matrix sections should therefore be read as a mechanistic research agenda and not as completed experimental validation of all matrix-dependent claims. Direct extension of ZnO-derived trends to SnO_2_, Fe_2_O_3_, or NiO requires equivalent cation loading, identical thermal-treatment windows, matrix-specific undoped controls, and the same XRD/FE-SEM/EDX/XPS/Raman/FT-IR evidence chain before quantitative conclusions can be drawn.

The main limitation of this study is that the broader literature comparison is inherently heterogeneous. Some studies are based on thin films, some on nanoparticles, and others on nanorods, hybrid structures, or composite powders. The synthesis routes also cover a broad methodological spectrum, including sol–gel processing, hydrothermal synthesis, sputtering, spray pyrolysis, and precipitation-based routes. This heterogeneity makes it difficult to completely separate the effect of dopant chemistry from the influence of processing conditions. Therefore, the conclusions presented in this study should not be interpreted as absolute quantitative superiorities. Rather, they should be understood as recurring structural, morphological, interfacial, and strategic trends. In this sense, the study does not establish reductionist judgments such as “boron is superior to rare-earth elements under all conditions” or “rare-earth elements are always more functional.” Instead, it identifies the conditions under which each dopant family offers a more balanced, more scalable, or more functionally powerful profile.

The second limitation arises from the characterization difficulty associated with boron. Because boron is a low-atomic-number element, its direct detection and quantitative localization are more challenging than those of rare-earth elements. In many studies, the presence and role of boron are inferred through indirect signatures rather than through direct evidence of its atomic position. This does not weaken the relevance of boron-based studies, but it does require greater caution in mechanism assignment. Similarly, although demonstrating the presence of rare-earth elements is relatively easier, distinguishing true lattice incorporation from surface segregation, grain-boundary enrichment, or nanoscale oxide formation is not always straightforward. Therefore, both dopant families have specific analytical blind spots, but the nature of these blind spots differs. Clearly articulating this distinction is essential for maintaining the integrity of the comparison.

The third limitation concerns the relationship between structural characterization and application performance. Application results cannot always be matched with structural data in a direct one-to-one manner. Two samples with similar crystallinity or morphology may exhibit different performance under different test configurations. In photocatalysis, the light source, pollutant type, initial concentration, solution pH, and catalyst loading can substantially alter the result. In gas-sensor studies, target gas concentration, operating temperature, humidity, flow regime, and baseline resistance strongly affect performance. In optoelectronic applications, substrate type, film thickness, device architecture, and contact quality can determine the measured output. For this reason, the present study uses application performance as a complementary layer rather than as the central axis of comparison. Nevertheless, if future studies adopt standardized testing protocols, the trends discussed here can be transformed into sharper quantitative and statistically comparable conclusions.

A related methodological limitation is that the morphological part of the primary synthesis is not statistically normalized across all systems. The study therefore avoids a universal particle-size or agglomeration ranking and treats FE-SEM/EDX evidence as a morphology-supporting layer unless the original study reports compatible numerical descriptors.

Finally, the policy-oriented interpretation is context dependent. The public-priority recommendations presented in this article are developed particularly with reference to Türkiye’s advantageous position in boron resources and related industrial infrastructure. For a different country, region, or economic bloc, the same recommendation may require reformulation depending on local production capacity, import dependence, trade agreements, refining infrastructure, technological specialization, and industrial structure. Nevertheless, the fundamental principle remains generalizable: in material selection, critical raw material risk and strategic dependence are not external variables separate from technical performance. They are internal components of the decision matrix. This understanding constitutes a broader contribution that extends beyond the specific case of Türkiye.

## 27. Conclusions

The integration of the primary experimental study set, particularly the ZnO–B_4_C, ZnO–h-BN, ZnO–TiC, and NiO–Y_2_O_3_ series, provides a more empirically grounded basis for the central thesis of this study. Accordingly, boron- and rare-earth-based additive strategies can be reconsidered not only through secondary literature synthesis, but also through direct characterization evidence involving crystallographic response, morphology, interface formation, defect chemistry, and low-frequency dielectric behavior.

This study proposed an integrated framework for evaluating boron-based and rare-earth-based additive strategies by combining materials-science evidence with critical raw material policy. Using ZnO as the common comparative matrix, this study demonstrates that direct lattice doping, secondary-phase composite formation, and interface engineering should not be interpreted as equivalent material-design routes. The main conclusion is that boron-based and rare-earth-based systems operate through fundamentally different mechanisms. Boron-based strategies, particularly direct B doping and h-BN-mediated interfacial architectures, generally provide stronger phase preservation, lower agglomeration tendency, more regular grain development, and more homogeneous surface organization. Rare-earth-based strategies, especially those involving Ce, La, and Y, can enable oxygen-vacancy engineering, redox activity, emission tuning, and pronounced functional improvements in selected applications; however, these advantages are usually accompanied by narrower processing windows, higher risks of secondary-phase formation or segregation, and greater supply-chain vulnerability.

The primary study set further refines this conclusion by showing that neither dopant family should be treated as internally homogeneous. On the boron side, B_4_C/ZnO and h-BN/ZnO represent different interfacial regimes: B_4_C acts as a mechanically rigid composite component that can support lattice expansion and crystallite growth, whereas h-BN behaves as a two-dimensional interfacial barrier that restricts crystallite growth and strengthens interface-mediated polarization. On the rare-earth side, the NiO–Y_2_O_3_ pathway shows that rare-earth modification does not necessarily have to maximize permittivity to be valuable; rather, it can generate a controlled defect/interface window with limited leakage and a more disciplined dielectric response. Control systems such as ZnO–TiC, ZnO–CNT/TiB_2_, NiO–V_2_O_5_, and NiO–TiN also demonstrate that dielectric enhancement is governed not only by dopant identity, but also by interfacial resistance contrast, defect enrichment, conductive network formation, and proximity to percolative conduction. Therefore, high functional output must always be interpreted together with dielectric loss, AC conductivity, phase stability, and morphology.

From both scientific and policy perspectives, the most defensible approach is to evaluate dopant strategies not along a single “performance” axis, but at the intersection of structural stability, morphological controllability, application-specific requirements, processing tolerance, technology-readiness potential, and critical raw material security. Within this multidimensional framework, boron-based strategies emerge as strong priority candidates for broad, scalable, and application-oriented research platforms, particularly in countries such as Türkiye, where domestic boron resources create a strategic value-chain advantage. Rare-earth-based systems, however, should not be excluded. Instead, they should be supported selectively in niche applications where their functional contribution is clearly demonstrated, reproducible, and difficult to substitute with boron-based or more accessible alternatives.

The final contribution of this study is that it makes the relationship between laboratory evidence and public prioritization visible. In the era of critical raw materials, materials science does not only generate crystal structures, defect landscapes, and surface morphologies, but also shapes supply-chain choices, industrial pathways, and technological sovereignty scenarios. Therefore, future advanced materials programs should prioritize not merely the systems that perform best under isolated laboratory conditions, but those that combine technical performance with reproducibility, scalability, supply security, circularity, and strategic defensibility.

## Figures and Tables

**Table 1:** Scope, inclusion criteria, and comparison logic.

Scale	Inclusion Criteria	Exclusion Criteria	Role in This Study
Common matrix	ZnO-based systems	Studies with different host matrices	To enable comparison within the same crystal and defect chemistry framework
Boron family	B doping, B_4_C/ZnO, hBN/ZnO	Boron derivatives unrelated to these systems	To evaluate direct doping + composite formation + interface strategies together
Rare-earth family	Ce/CeO_2_, La/La_2_O_3_, Y/Y_2_O_3_	Different REE groups	To examine ionic-size and functional differences within a limited but representative set
Characterization	XRD, FE-SEM, EDX, XPS, Raman or FT-IR	Studies reporting only performance data	To improve the quality of structural and morphological evidence
Source type	DOI indexed peer reviewed articles and official reports	Sources without DOI, unverified records, or secondary summary texts	To maintain citation reliability

**Table 2:** Main scientific reasons supporting the selection of ZnO as the common comparison matrix.

Criteria	Advantage of ZnO	Significance for Comparison
Crystal structure	Wurtzite structure and distinct phase signature	Peak shifts and phase segregation induced by dopants become more visible
Defect chemistry	Sensitivity to oxygen vacancies and surface defects	Structural and functional effects of dopants can be interpreted together
Synthetic diversity	Sol–gel, hydrothermal synthesis, sputtering, CVD, etc.	Different dopants can be compared on the same platform
Application range	Sensors, photocatalysis, films, photonics, and energy	The application specific significance of the dopant can be identified
Literature density	Strong publication base for both boron and rare-earth systems	Provides a sufficient database for comparative review

**Table 3:** Selected representative studies and structural and morphological inferences for boron-based ZnO strategies.

Representative Strategy/Study	Main Structural Effect	Main Morphological Effect	Comparative Interpretation
B-doped ZnO films [33]	Crystal structure and electrical behavior change in a concentration dependent manner	More regular film morphology at appropriate dopant levels	Strong example of direct lattice and defect tuning
B-doped transparent conducting films [48]	Conductivity is tuned while preserving the main ZnO phase	Continuous and technologically transferable film structure	Demonstrates the scalable thin film advantage of boron
Sol–gel B-doped films [52]	Surface properties change together with phase preservation	Homogeneity and photocatalytic surface behavior can be improved	Makes the structure and surface relationship visible
B_4_C/ZnO composite [37]	The secondary phase is part of the design	Interface controlled composite topology	Composite engineering, not conventional doping
ZnO/BN quantum dot hybrid [42]	Interface driven charge separation	Improved dispersion and surface utilization	Supports the agglomeration resistance of h-BN/BN-based strategies
ZnO growth on h-BN [29]	Controlled growth via van der Waals epitaxy	Architectured and oriented nanostructure	One of the strongest interface focused examples of the boron family

**Table 4:** Selected representative studies and structural and morphological inferences for rare-earth-based ZnO strategies.

Studies	Main Structural Effect	Main Morphological Effect	Comparative Interpretation
Ce-doped ZnO nanorods [43,55]	Ce mediated tuning of defect and optical states	Morphology strongly depends on synthesis conditions	High functionality, narrow processing window
Ce-doped photocatalytic ZnO [68]	Enhanced surface redox activity	Performance enhancement accompanied by segregation risk	Demonstrates the powerful but fragile nature of Ce
ZnO/CeO_2_ heterostructures [38,59]	Heterojunction formation and oxygen storage effects	Multiphase active surface	Functional intensification based on secondary phases
La-doped ZnO [44,60]	Modification of lattice strain and surface chemistry	Grain boundary effects and potential heterogenization	Provides conditional performance enhancement
ZnO/La_2_O_3_ composite [39]	Active heterophase design	Topology highly sensitive to dispersion quality	Can provide strong photocatalytic performance when the interface is well controlled
Y-doped ZnO [36,45]	UV emission and electronic property tuning	Thin film texture can be controllably modified	Relatively balanced subgroup within the REE family

**Table 5:** Interpretive strengths and limitations of XRD, FE-SEM, EDX, XPS, Raman, and FT IR data.

Technique	Strongest Output	Main Limitation	Usage Principle in This Study
XRD	Phase identification, peak shifts, and crystallite size trends	Does not independently prove the actual dopant incorporation site	Interpreted in conjunction with XPS and morphological evidence
FE-SEM	Agglomeration, grain size, and surface homogeneity	The representativeness of the imaged area may be limited	Used for qualitative observation and comparative morphology
EDX	Elemental mapping and surface distribution	Sensitivity is low for boron	Strong for REE systems; cautious interpretation for boron
XPS	Chemical state and surface localization	Peak deconvolution is interpretation dependent	One of the key pieces of evidence for the dopant mechanism
Raman	Sensitivity to defect modes and weak secondary phases	Assignments may vary from system to system	Provides complementary validation to XRD
FT-IR	Surface bonds, B-O/B-N, and residual species	Not independently specific in all cases	Supporting evidence for interface and bonding interpretation

**Table 6:** Structural and morphological comparison matrix of boron-based and rare-earth-based strategies.

Criteria	Boron-Based Strategies	Rare-Earth-Based Strategies	General Assessment
Phase preservation	Often higher in direct B doping	More frequent segregation or secondary phase formation	In favor of boron
Secondary phase behavior	Designed and predictable in B_4_C/h-BN systems	Frequently synthesis dependent and more variable	In favor of boron in terms of predictability
Agglomeration	Lower; especially suppressed in h-BN systems	More pronounced clustering at high dopant loadings	In favor of boron
Grain size control	More uniform and balanced refinement	Grain refinement may occur together with heterogeneity	In favor of boron
Redox and defect activity	More limited but better controlled	Especially strong for Ce	In favor of rare earth
Optical and luminescence tuning	Indirect and limited	More pronounced for Ce and Y	In favor of rare earth
Processing window	Broader and closer to scalability	Narrower and more process sensitive	In favor of boron

**Table 7:** Decision matrix for the joint interpretation of technical performance and critical raw material policy.

Policy Criterion	Boron-Based Strategies	Rare-Earth-Based Strategies	Public R&D Outcome
Security of supply	More manageable from Türkiye’s perspective	Imported, concentrated, and vulnerable	Priority for boron
Strategic dependence	Relatively low	High	Selective use of rare-earth elements
Value chain deepening	Can extend from mining to advanced materials	Refined product dependency is significant	Boron focused platform programs
Functional indispensability	Sufficient for many broad applications	Strong superiority in some niche applications	Use REE-based strategies only in justified application areas
Scalability	Higher process manageability	Higher challenges in homogeneity and supply security	Industrial transfer in favor of boron
Logic of funding support	Can be positioned as the default research platform	Requires targeted and well justified research programs	Two stage incentive model

**Table 8:** Proposed comparative research design that can be directly transformed into an experimental article.

Experimental Element	Boron Branch	Rare-Earth Branch	Common Output
Common matrix	ZnO	ZnO	Directly comparable structure
Direct dopant series	B: 0.5–5 mol%	Ce/La/Y: 0.5–5 mol%	Comparison of phase preservation and defect effects
Composite series	B4C, h-BN: 1–5 wt.%	CeO_2_, La_2_O_3_, Y_2_O_3_: 1–5 wt.%	Comparison of interface and secondary phase behavior
Synthesis route	Single selected common method	Same method	To reduce process induced deviations
Characterization	XRD, FE-SEM, EDX, XPS, Raman, FT-IR	Same set	To establish an evidence standard
Main response variables	Phase purity, agglomeration, grain size, and homogeneity	Same variables	Direct comparison matrix
Policy interpretation	Advantage of domestic resource availability	Supply vulnerability	Technical data and strategic decision model

**Table 9:** Mechanistic comparison framework for the transferability of ZnO-centered findings to other oxide matrices.

Matrix	Dominant Crystal and Electronic Property	Expected Primary Effect of Boron-Based Dopants	Expected Main Effect of Rare-Earth Dopants	Main Risk	Strategic Interpretation
ZnO	Wurtzite structure; predominantly n type; oxygen vacancies and interstitial defects are important	Suppression of grain growth, control of agglomeration, and tuning of the transparency and conductivity balance	Modifying oxygen vacancies and surface-active sites, while modulating photoluminescence and photocatalysis	Secondary phase formation and heterogeneity at high dopant loadings	Boron as the default low-risk starting point; RE as a selective tool for specific functions
SnO_2_	Rutile structure; n type behavior; surface adsorption and redox processes are critical	Limited lattice substitution plus grain boundary and surface modification	Tuning redox and sensing behavior through Ce/La/Y incorporation	Solubility limit, segregation, and secondary oxide phases	RE-based dopants can offer performance gains, but within a narrow processing window
Fe_2_O_3_	Hematite structure; short carrier diffusion length; high recombination	Potential to improve surface states and charge separation	Reorganization of oxygen vacancies and surface redox centers	Misinterpretation of dopant distribution and uncertainty in the surface and bulk distinction	Boron-based dopants for high-volume applications; RE-based dopants for niche redox architectures
NiO	Cubic structure; predominantly p type behavior; Ni vacancies are critical	Optical and morphological tuning, with control over surface roughness and transmittance	Energy level alignment and carrier concentration tuning	Carrier type reversal, segregation, and high process sensitivity	A dual strategy is required depending on the application; no single dopant family is absolutely superior

**Table 10:** Dominant mechanisms, expected characterization signatures, and primary risks associated with boron and rare-earth dopants.

Mechanistic Dimension	Typical Response of Boron-Based Dopants	Typical Response of Rare-Earth-Based Dopants	Expected Characterization Signature	Risk of Misinterpretation
Lattice substitution	Limited; often local bonding rearrangement at low dopant levels	Stronger but constrained by the solubility limit	XRD peak shift and XPS binding energy change	Mistaking surface enrichment for homogeneous doping
Defect compensation	Local bond stiffness and interstitial/vacancy balance in some systems	Oxygen vacancies and valence changes are more pronounced	XPS O 1s deconvolution and Raman defect bands	Overinterpreting the O 1s shoulder alone
Grain boundary effect	Pinning and tendency toward a fine-grained structure	Segregation or selective nucleation	FE-SEM, XRD crystallite size, and EDX mapping	Generalization based on a single micrograph
Surface chemistry	Subtle modulation of acid base and interfacial bonding character	Strong redox and adsorption center effect	XPS, FT IR, and application tests	Inability to link performance enhancement to a structural origin
Secondary phase formation	Possibility of borate, B_2_O_3_, or B_4_C related intermediate phases at high dopant loadings	RE oxide clusters or adjacent phases are more likely	Weak peaks in XRD and additional bands in Raman	Assuming the absence of secondary phases because they are not visible in XRD

**Table 11:** Proposed minimum experimental and reporting standard for the direct comparison of boron and rare-earth dopants.

Component	Minimum Requirement	Why Is It Necessary?	Problem Arising When Absent
Dopant amount	Atomic percentage or cation molar ratio alongside nominal wt.%	To establish true chemical equivalence	Chemically non-equivalent loading at the same wt.%
Synthesis conditions	Precursor chemistry, pH, solvent system, aging, drying, annealing rate, and atmosphere	To distinguish dopant effects from processing effects	Incorrect attribution of morphological differences to the dopant
Phase analysis	XRD with peak broadening and strain analysis where possible	Primary phase preservation and secondary phase control	Structural cost of performance enhancement remains invisible
Morphology	Multi-area FE-SEM coupled with quantitative image analysis	To statistically assess homogeneity and agglomeration	Biased conclusion based on a single selected image
Chemical state	EDX mapping and XPS	To verify dopant distribution and surface chemistry	Homogeneous doping assumption
Defect and bonding analysis	Raman and/or FT IR	To identify lattice distortion and interfacial bonding	Weak mechanistic interpretation
Reproducibility	Independent synthesis replicates and statistical reporting	Reliability and peer-review strength	Generalization of a random result

**Table 12:** Scenario-based multi-criteria decision-making framework and relative positioning of dopant families.

Criterion Group	Typical Subcriteria	Academic Discovery Weight	Industrial Scale-Up Weight	Public Strategy Weight	General Trend
Technical performance	Phase purity, defect management, target function, and surface homogeneity	High	Medium to high	Medium	Rare-earth dopants may outperform in certain niche applications
Manufacturability	Processing window, reproducibility, quality control, and synthesis complexity	Medium	High	High	Boron-based strategies are often more advantageous
Supply security	Supply concentration, geopolitical risk, and price volatility	Medium	High	Very high	Boron-based strategies have a clear advantage
Circularity	Recovery, waste management, and secondary raw material potential	Low to medium	Medium	Medium to high	Recovery is critical but challenging for REE systems
National alignment	Domestic resources, industrial integration, and alignment with public programs	Low	Medium	Very high	In favor of boron in the context of Türkiye

**Table 13:** Policy profile of boron-based and rare-earth-based dopant families in terms of critical raw material governance.

Policy Dimension	Boron-Based Dopants	Rare-Earth-Based Dopants	Implication for Research Management
Supply concentration	Relatively advantageous and more manageable in the context of Türkiye	Global supply is more concentrated and geopolitically more sensitive	Boron-based pathways should play a core role in the portfolio
Dependence on processing and refining capacity	Can be configured more flexibly depending on the application context	More dependent on high purity materials and specialized processing steps	Rare-earth studies should be supported with selective justification
Price volatility	Comparatively more manageable	Higher uncertainty and sensitivity to external shocks	RE risk is higher in long term project planning
Recycling and secondary flows	Can be developed, but application specific design is required	Recovery is critical but technically and economically challenging	Circularity design should be planned from the outset
National resource alignment	High in the context of Türkiye	Low to moderate; often import dependent	Public funding priority shifts in favor of boron
Scientific justification threshold	Sufficient technical performance and scalability	Requires non-substitutable technical superiority	The evidence standard should be higher for RE projects

**Table 14:** Comparative risk matrix of dopant strategies in terms of technology readiness level and scale-up.

Evaluation Dimension	Boron-Based Dopants	Rare-Earth-Based Dopants	Main Reason for High Risk	Recommended Management Strategy
Laboratory synthesis	Generally manageable	Manageable but sensitive	Precursor reactivity and solubility differences	Atomic percentage-based experimental series
Pilot scale homogeneity	Medium risk	Medium to high risk	Segregation and within batch distribution differences	Multi-point quality control and process-window mapping
Raw material access	Low–medium risk	High risk	Supply concentration and external dependence	Supply diversification and substitution plan
Material recovery	Medium risk	Medium to high risk	Low concentration complex matrices	Circularity criterion in product design
Cost volatility	Relatively lower	Higher	Market and geopolitical volatility	Long term supply scenario
Technology readiness level progression	More linear	More selective and more vulnerable	Failure to preserve technical superiority during processing	Application-based threshold decisions

**Table 15:** Comparative decision map for dopant family selection across major application areas.

Application Area	Priority Technical Criterion	Typical Advantage of Boron-Based Dopants	Typical Advantage of Rare-Earth-Based Dopants	Default Strategic Preference
Gas sensor	Surface active sites and morphological stability	More homogeneous microstructure and process robustness	Stronger adsorption and redox modulation	Boron for high volume applications; RE may be considered for selective niche sensors
Photocatalysis	Charge separation, surface area, and reusability	Interface engineering with h BN/B_4_C and lower supply risk	Strengthening of redox centers	In favor of boron when sufficient performance is achieved
Photoelectrochemistry	Charge transport and stability	Surface states and structural stability	Specific redox and energy level tuning	In favor of boron under long lifetime and cost constraints
Optoelectronics	Transmittance and conductivity balance or energy alignment	Simple and scalable thin film chemistry	Advantages in PL and energy level engineering	Context dependent according to the device target
Energy storage	Impedance, cycle life, and capacitance	Cost advantage and broad application base	Enhanced electrochemical activity in selected cases	In favor of boron for high volume products, with selective use of RE in niche designs

**Table 16:** Testable hypotheses, validation methods, and expected strategic outcomes for future research.

Hypothesis Code	Hypothesis	Minimum Validation Set	Expected Scientific Outcome	Expected Strategic Outcome
H1	Boron-based dopants produce a narrower grain size distribution within the same matrix	XRD, multi field FE SEM, and quantitative image analysis	Quantitative evidence of morphological stability	Scalable platform selection
H2	REE dopants produce higher functional enhancement within a low loading window, but their processing window is narrow	XRD, XPS, application testing, and dopant loading series	Demonstration of threshold behavior	Criterion for selective RE use
H3	ZnO findings are partially transferable to SnO_2_ and Fe_2_O_3_, but become context dependent in NiO	Multi matrix series using the same protocol	Cross matrix transferability model	Generalizable research platform
H4	Boron and RE dopants act through different defect mechanisms	XPS, Raman, FT IR, and performance correlation	Mechanism differentiation	Targeted dopant selection
H5	Boron-based systems achieve higher total technological value when the strategic weight increases	MCDA with supply and data sensitivity analysis	Decision model	Design of public funding priorities
H6	REE use is rational only when it provides a non-substitutable technical advantage	Counterfactual comparison and substitution analysis	Justification threshold	Reduction in dependence in the research portfolio

**Table 17:** Methodological warning signs frequently observed in comparative dopant studies.

Methodological Red Flag	Typical Appearance	Reason for Methodological Concern	Correction Recommendation
Doping and composite formation confusion	Surface enrichment is presented as intralattice doping.	The mechanism is incorrectly constructed.	Clearly classify the strategy type from the outset.
Non-equivalent dopant level	B and RE are compared on the basis of the same wt.%.	Chemical equivalence is disrupted.	Use at.% or cation ratio.
Selected image	A single favorable micrograph is presented as representative of the entire sample.	Morphology cannot be generalized.	Perform multi area sampling and quantitative analysis.
Peak performance bias	Only the best replicate is reported.	Reproducibility remains invisible.	Report error bars and the number of replicates.
Non-normalized testing	Measurements obtained under different experimental conditions are compared.	The dopant effect is overstated.	Apply an application specific normalization set.
Overgeneralized policy implication	Broad strategic conclusions are drawn from a narrow dataset.	The level of evidence is exceeded.	Formulate conditional and scenario-based recommendations.

**Table 18:** Methodological backbone of the primary study set and its comparative function within the study.

Ref.	System	Methodological Backbone	Depth	Role in This Study
[16]	ZnO–TiC	XRD, FE-SEM/EDX, FT-IR, impedance	Medium	Control case for carbide interface induced lattice expansion and dielectric enhancement
[17]	ZnO–B_4_C	XRD, FE-SEM/EDX, FT-IR, impedance	Medium	Primary example of balanced structural and dielectric enhancement on the boron carbide side
[18]	ZnO–h-BN	XRD, FE-SEM/EDX, FT-IR, Repeated impedance measurements	Medium to high	Primary example of two-dimensional sheet interface architecture and strong interfacial polarization
[19]	ZnO–CNT/TiB_2_	XRD, FE-SEM/EDX, FT-IR, Raman, impedance	High	ZnO control case distinguishing percolation and MWS polarization
[20]	NiO–Y_2_O_3_	XRD, W–H, Rietveld, FE-SEM/EDX, FT-IR, Raman, XPS, dielectric	Higher	Controlled reinforcement model driven by defect and interface effects on the rare earth side
[21]	NiO–V_2_O_5_	XRD, W–H, FE-SEM/EDX, FT-IR, Raman, XPS, dielectric	Higher	Explanatory control pathway for non-REE defect and interface engineering
[22]	NiO–TiN	XRD, W–H, FE-SEM/EDX, FT-IR, Raman, XPS, broadband dielectric	Higher	An interfacial model illustrating conductivity contrast and the microcapacitor network effect

**Table 19:** Reported synthesis and testing anchors of the primary experimental study set and level of methodological equivalence.

Ref.	System/Matrix	Additive Loading Used in the Primary Study	Reported Synthesis/Testing Anchor	Comparability Implication
[16]	ZnO–TiC	TiC-reinforced ZnO: 10, 20, and 30 wt.%	Sol–gel nanoparticle synthesis; solvents dissolved under magnetic stirring at 90 °C for 4 h; filtration, drying, pestle grinding, and annealing; XRD/FE-SEM/EDX/FT-IR plus impedance; dielectric anchor used here: 1 kHz.	ZnO carbide-interface control. Comparable to boron systems only at the interface/polarization-mechanism level, not as a rare-earth or lattice-doping equivalent.
[17]	ZnO–B_4_C	B_4_C/ZnO nanocomposite series; highest dielectric value discussed at 30 wt.% B_4_C.	Sol–gel preparation of pure ZnO and B_4_C-doped ZnO nanocomposite particles; XRD, FE-SEM/EDX, FT-IR, and impedance; dielectric anchor used here: 10 kHz.	Boron-carbide heterophase pathway. Interpreted as composite/interface engineering rather than direct lattice B substitution.
[18]	ZnO–h-BN	h-BN-reinforced ZnO: 1, 5, and 10 wt.%	Sol–gel route using zinc acetate dihydrate, NaOH, and h-BN; magnetic stirring at 90 °C for 4 h; XRD/FE-SEM/EDX/FT-IR and impedance; dielectric anchor used here: 1 kHz.	Two-dimensional sheet-interface pathway. Comparable to ZnO–B_4_C only mechanistically, because the additive geometry and polarization pathway differ.
[19]	ZnO–CNT/TiB_2_	Graphene-hybridized CNT and TiB_2_ nanomaterial additions in the 1, 5, and 10 wt.% range.	Sol–gel synthesis; FT-IR, XRD, FE-SEM/EDX, Raman, and impedance analysis; impedance anchor used here: 1 kHz.	Control for conductive-network/percolation and boride-interface effects. Not used as a simple dielectric benchmark because CNT strongly alters σac.
[20]	NiO–Y_2_O_3_	Y_2_O_3_-reinforced NiO: 1–10 wt.%; highest dielectric value discussed at 10 wt.% Y_2_O_3_.	Controlled nonaqueous sol–gel route; XRD with Williamson–Hall/Rietveld analyses, FE-SEM/EDX, FT-IR, Raman, XPS, and dielectric testing; anchor used here: 1 kHz.	Rare-earth pathway in a different p-type matrix. Used to identify controlled defect/interface behavior, not to rank directly against ZnO composites.
[21]	NiO–V_2_O_5_	V_2_O_5_-modified NiO: 1, 2, 3, 5, and 10 wt.%	CTAB-assisted sol–gel route; XRD/Williamson–Hall, FE-SEM/EDX, FT-IR, Raman, XPS, and dielectric/AC transport; anchor used here: 1 kHz.	Non-REE defect/interface control for NiO. Separates oxygen-vacancy/interface mechanisms from rare-earth identity.
[22]	NiO–TiN	TiN-containing NiO–TiN nanopowders: 1–10 mol.% TiN.	Sol–gel-assisted controlled precipitation; XRD/Williamson–Hall, FE-SEM/EDX, FT-IR, Raman, XPS, and broadband dielectric/AC transport; dielectric anchor discussed here: 1 kHz.	Control for conductive contrast and microcapacitor-network effects. Used to distinguish interface-driven permittivity from dopant-family ranking.

**Table 20:** Comparative dielectric and AC conduction indicators of selected systems at the low-frequency anchor; values are extracted from refs. [16,17,18,19,20,21,22] and interpreted with the frequency column.

System	f (kHz)	ε′ Matrix	Highest ε′	σ_ac_ Matrix (S/cm)	Highest σ_ac_ (S/cm)	Brief Interpretation
ZnO–B_4_C	10	12.077	18.340	8.35 × 10^−9^	1.02 × 10^−7^	Moderate ε′ enhancement with a balanced composite interface on B_4_C sheets.
ZnO–h-BN	1	12.070	55.219	8.35 × 10^−9^	3.59 × 10^−8^	Very strong interfacial polarization with high ε′ accompanied by increased dielectric loss.
ZnO–TiC	1	12.07	26.26	8.35 × 10^−9^	8.65 × 10^−8^	Carbide interface effect, with simultaneous increases in ε′ and ε″
ZnO–TiB_2_	1	19.260	30.004	1.07 × 10^−8^	1.69 × 10^−8^	Moderate and relatively controlled enhancement as a boride control system.
ZnO–CNT	1	19.260	204.885	1.07 × 10^−8^	1.32 × 10^−4^	Percolative conductive network; caution is required in pure dielectric comparisons.
NiO–Y_2_O_3_	1	6.607	9.327	1.602 × 10^−8^	2.027 × 10^−8^	Controlled REE enhancement with a low leakage interface.
NiO–V_2_O_5_	1	8.04	13.12	3.30 × 10^−8^	5.71 × 10^−8^	Non-REE defect and interface pathway as a mechanism distinguishing control.
NiO–TiN	1	7.155	14.854	reported dispersive	reported increasing	Conductive nitride interface; ε′ increases while tanδ remains in a narrow 0.070–0.087 interval, indicating controlled loss.

Note: Because the frequency windows, matrices, loading descriptors, and electrode/testing configurations are not identical, the table reports the low-frequency anchor used for each system and should be read as a structured evidence table rather than as a normalized ranking of intrinsic material superiority. The technically relevant comparison is the coupled change in ε′, dielectric loss or loss tendency, and σ_ac_. A high ε′ value is interpreted as advantageous only when it is not primarily driven by uncontrolled leakage, percolative conduction, or a disproportionate increase in AC conductivity.

## Data Availability

All analytical inferences used in this article were derived from the sources cited in the text. No new experimental dataset was generated within the scope of this review.
